# A Theobromine Derivative with Anticancer Properties Targeting VEGFR‐2: Semisynthesis, *in silico* and *in vitro* Studies

**DOI:** 10.1002/open.202300066

**Published:** 2023-10-06

**Authors:** Ibrahim H. Eissa, Reda G. Yousef, Hazem Elkady, Eslam B. Elkaeed, Aisha A. Alsfouk, Dalal Z. Husein, Ibrahim M. Ibrahim, Mohamed M. Radwan, Ahmed M. Metwaly

**Affiliations:** ^1^ Pharmaceutical Medicinal Chemistry & Drug Design Department Faculty of Pharmacy (Boys) Al-Azhar University Cairo 11884 Egypt; ^2^ Department of Pharmaceutical Sciences College of Pharmacy AlMaarefa University Riyadh 13713 Saudi Arabia; ^3^ Department of Pharmaceutical Sciences College of Pharmacy Princess Nourah bint Abdulrahman University P.O. Box 84428 Riyadh 11671 Saudi Arabia; ^4^ Chemistry Department Faculty of Science New Valley University El-Kharja 72511 Egypt; ^5^ Biophysics Department Faculty of Science Cairo University Giza 12613 Egypt; ^6^ National Center for Natural Products Research University of Mississippi Mississippi MS 38677 USA; ^7^ Department of Pharmacognosy Faculty of Pharmacy Alexandria University Alexandria Egypt; ^8^ Pharmacognosy and Medicinal Plants Department Faculty of Pharmacy (Boys) Al-Azhar University Cairo 11884 Egypt; ^9^ Biopharmaceutical Products Research Department Genetic Engineering and Biotechnology Research Institute City of Scientific Research and Technological Applications (SRTA-City) Alexandria Egypt

**Keywords:** apoptosis, CAAD, DFT, MD simulations, VEGFR-2

## Abstract

A computer‐assisted drug design (CADD) approach was utilized to design a new acetamido‐*N*‐(para‐fluorophenyl)benzamide) derivative of the naturally occurring alkaloid, theobromine, (**T‐1‐APFPB**), following the pharmacophoric features of VEGFR‐2 inhibitors. The stability and reactivity of **T‐1‐AFPB** were assessed through density functional theory (DFT) calculations. Molecular docking assessments showed **T‐1‐AFPB**’s potential to bind with and inhibit VEGFR‐2. The precise binding of **T‐1‐AFPB** against VEGFR‐2 with optimal energy was further confirmed through several molecular dynamics (MD) simulations, PLIP, MM‐GBSA, and PCA studies. Then, **T‐1‐AFPB** (4‐(2‐(3,7‐Dimethyl‐2,6‐dioxo‐2,3,6,7‐tetrahydro‐1*H*‐purin‐1‐yl)acetamido)‐*N*‐(4‐fluorophenyl)benzamide) was semi‐synthesized and the *in vitro* assays showed its potential to inhibit VEGFR‐2 with an IC_50_ value of 69 nM (sorafenib's IC_50_ was 56 nM) and to inhibit the growth of HepG2 and MCF‐7 cancer cell lines with IC_50_ values of 2.24±0.02 and 3.26±0.02 μM, respectively. Moreover, **T‐1‐AFPB** displayed very high selectivity indices against normal Vero cell lines. Furthermore, **T‐1‐AFPB** induced early (from 0.72 to 19.12) and late (from 0.13 to 6.37) apoptosis in HepG2 cell lines. In conclusion, the combined computational and experimental approaches demonstrated the efficacy and safety of **T‐1‐APFPB** providing it as a promising lead VEGFR‐2 inhibitor for further development aiming at cancer therapy.

## Graphical abstract

A new theobromine derivative, (**T‐1‐AFPB**), was designed as a VEGFR‐2 inhibitor. DFT calculations indicated **T‐1‐AFPB**’s stability and reactivity. **T‐1‐AFPB**’s potential binding and inhibition of VEGFR‐2 were indicated by molecular docking, MD simulations, PLIP, MM‐GBSA, and PCA studies. **T‐1‐AFPB**’s drug likeness was indicated several *in silico* ADMET investigations. Subsequently, **T‐1‐AFPB** was semi‐synthesized, and *in vitro* assays confirmed its potential to inhibit VEGFR‐2 and to inhibit the growth of HepG2 and MCF‐7 cancer cell lines, displaying very high selectivity indices and inducing apoptosis.

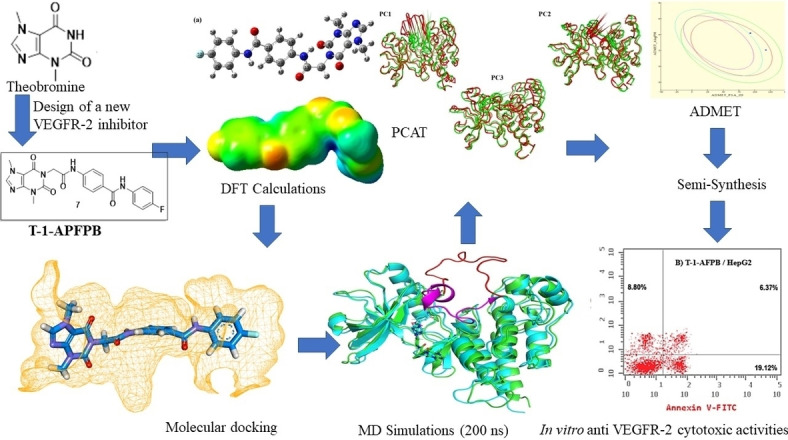

## Introduction

Cancer remains a life‐threatening disease that is a leading cause of death with globally raised incidence and mortality rates.^[1]^ The need for innovative, and safe anti‐tumor drugs is more pressing than ever, as conventional non‐selective chemotherapy drugs are associated with serious side effects and drug resistance.^[2]^ Therefore, the development of novel anticancer drugs that are highly selective, potent, and safe is a critical research area that promises to improve cancer treatment and patient outcomes. Vascular endothelial growth factor receptor‐2 (VEGFR‐2) is a transmembrane tyrosine kinase receptor that is considered a promising target for cancer treatment.^[3]^ VEGFR‐2 plays a vital role in regulating several critical processes in cancer development, including cell proliferation, motility, adhesion, and angiogenesis.^[4]^ Moreover, VEGFR‐2 overexpression has been associated with the metastasis of solid tumors.^[5]^ VEGFR‐2 expression is relatively high in various cancer types, such as prostate, breast, colon, cervical, and ovarian cancer.^[6]^ Therefore, the development of effective VEGFR‐2 inhibitors is crucial for the improvement of cancer treatment and management.

In recent years, there have been significant advancements in software and proteomics, which have led to a major breakthrough in the field of drug discovery.^[7]^ Specifically, computer‐aided drug discovery (CADD) has emerged as a powerful tool in the identification and development of novel anticancer drugs.^[8]^ CADD is a field of research that combines computational and experimental techniques to accelerate the drug discovery process. It allows for the prediction of the physical and chemical properties of molecules, as well as the identification of their interactions with different proteins, which are essential components of the cellular machinery.^[9]^ Moreover, CADD has evolved to become a highly sophisticated and accurate method for predicting the efficacy of different compounds and optimizing their chemical structure to enhance their therapeutic potential.^[10]^ CADD has greatly accelerated the drug discovery process, reducing the time and costs associated with traditional drug discovery methods and leading to the discovery of several novel anticancer drugs with high efficacy and low toxicity, making it a promising approach for future drug development.^[11]^

A range of promising anticancer candidates with VEGFR‐2‐inhibitory activity has been discovered by our laboratory depending on several computational methods. These candidates are derived from various classes and derivatives, such as quinoline,^[12]^ isatin^[12]^ nicotinamide,^[13]^ thiazolidine,^[14]^ pyridine,^[15]^ naphthalene,^[16]^ and indole.^[17]^ These candidates offer a diversified and extensive selection of compounds that may be explored further for their potential in cancer treatment.

Nature has been a reliable source of nourishment and healing for humans throughout history, providing us with vital resources like food, medicine, and cosmetics.^[18]^ The search for natural anti‐cancer drug discovery has led researchers to investigate xanthine derivatives, which have shown antimutagenic activities against prostate,^[19]^ ovarian,^[20]^ leukemia, and breast^[21]^ cancers. One of the xanthine derivatives, theobromine, is a xanthine alkaloid that was first discovered in 1841 and can be found in chocolate and tea leaves.^[22]^ *In vitro* and *in vivo* studies have found that theobromine can inhibit DNA synthesis and impede the growth of glioblastoma multiforme (U87MG cell line).^[23]^ Theobromine has also displayed anti‐angiogenic activities by inhibiting VEGF in both lung^[24]^ and ovarian^[25]^ cancers. These findings offer a potential opportunity for further research and the development of theobromine as an anti‐cancer agent.

The ATP binding pocket of VEGFR‐2 consists of four regions. Firstly, the adenine binding region (hinge region) which plays a crucial role in efficient binding. This region contains two essential amino acids, Cys917 and Glu 915.^[26]^ Secondly, The gatekeeper region which serves as a hydrophobic pocket acting as a spacer region between the hinge region and DFG motif region.^[27]^ Thirdly, the DFG motif region comprising the Asp1046 and Glu885 residues. The formation of hydrogen bonds (H‐bond) with these two amino acids is essential for proper fitting.^[28]^ Lastly, the allosteric pocket which is hydrophobic in nature.^[29]^

**Figure 1:**
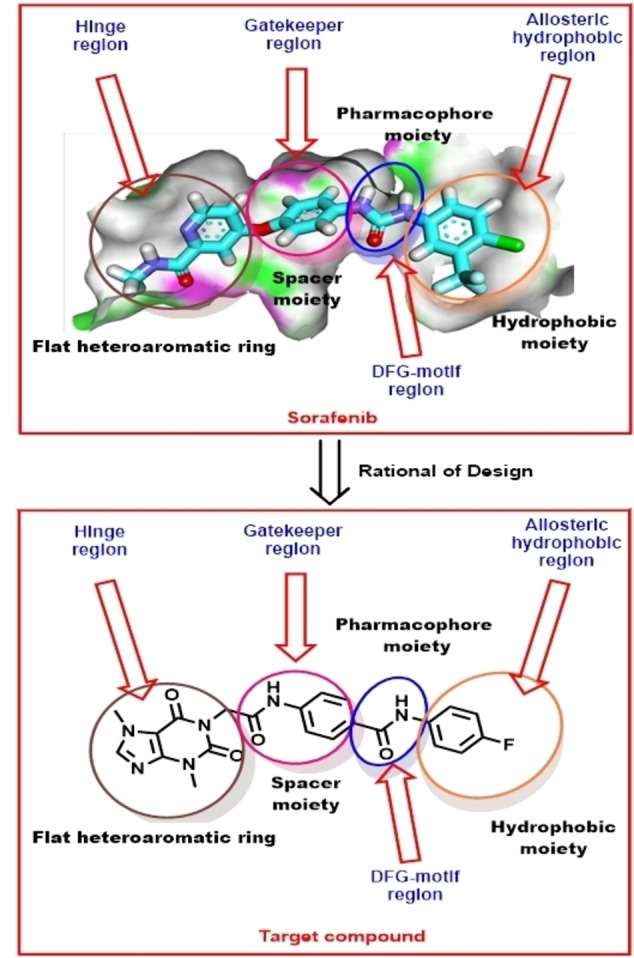
The rationale of **T‐1‐APFPB**’s design.

Depending on the aforementioned pharmacophoric features of VEGFR‐2, a new semisynthetic compound (**T‐1‐APFPB**) has been designed. As illustrated in Figure 1, The chemical structure of **T‐1‐AFPB** comprises a xanthine moiety which serves as a heteroaromatic structure designed to occupy the hinge region. Additionally, the N‐phenylacetamide moiety is intended to occupy the gatekeeper region. To facilitate the H‐bonding interaction with Asp1046 and Glu885 in the DFG motif region, an amide group is incorporated. Lastly, the hydrophobic tail, represented by the 4‐fluorophenyl moiety, will target the allosteric pocket.

## Results and Discussion

### Density Functional Theory (DFT) studies

**Figure 2:**
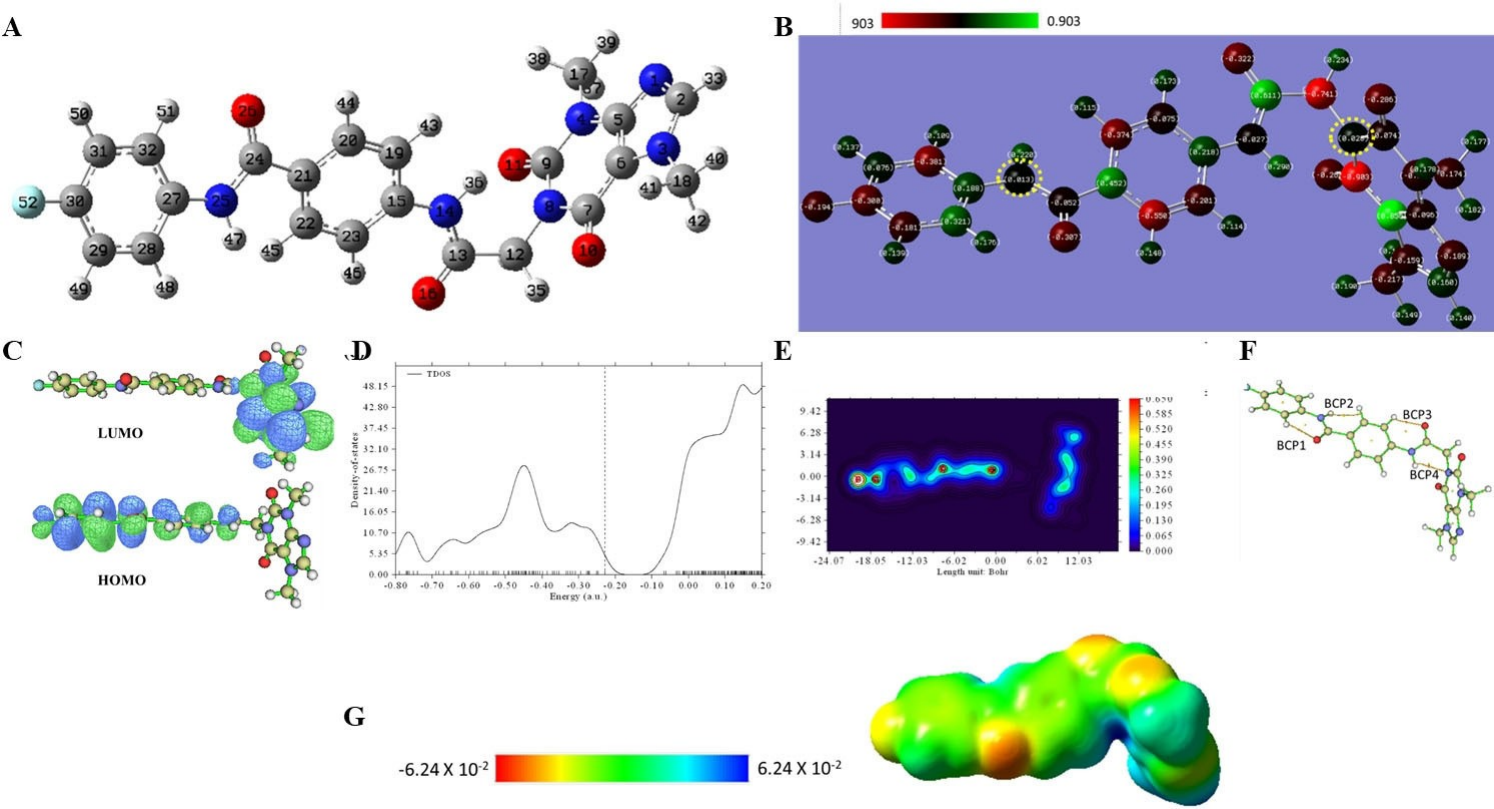
The optimized geometry (A), Mulliken charge distribution (B), the frontier molecular orbitals (C), the total density of state (D), QTAIM maps (E and F), and the electrostatic potential map (G), at B3LYB/6‐311+G(d,p) for **T‐1‐AFPB**.

The electrical structure and topology studies were conducted using the B3LYP/6‐311G+(d,p) level of theory. Quantum mechanics puts a greater focus on calculations of molecular orbital characteristics, dipole moments, total densities of states, atomic partial charges, and molecule electrostatic potentials, as well as an understanding of various forms of interactions. The Gaussian 09 software used density functional theory (DFT) and the B3LYP/6‐311G+(d,p) method to optimize the molecular geometry of **T‐1‐AFPB**. The fully optimized structure of **T‐1‐AFPB** with atom numbering scheme (Figure 2.A) showed that the purine moiety is slanted to the rest of the molecule. The total energy with zero‐point energy correction of the **T‐1‐AFPB** is −42981.8 eV where the calculated dipole moment (Dm) was 8.85 Debye, Table 1. The high Dm value supports a good charge separation and electronegativity of bonded atoms.

**Table 1:** The DFT calculated global reactivity parameters for **T‐1‐AFPB**.

IP	EA	μ (eV)	χ (eV)	η (eV)	σ (eV)	ω (eV)	Dm (Debye)	TE (eV)	ΔNmax	ΔE (eV)
−6.224	−1.815	−4.019	4.019	2.205	0.454	17.808	8.858	−42981.8	1.823	−17.808

Mulliken charges identify the molecule‘s chemically active regions, which are what give rise to the molecule‘s chemical reactivity. According to Figure 2.B, the Mulliken charge population along the geometry conducted that all heteroatoms are negative except for the two‐nitrogen labeled with dashed light blue. Most carbon atoms have a negative charge as well, so they could be reactive. Only a few carbon atoms appear in positive charge due to steric hindrance as represented in Figure 2.B.

The energies of frontiers orbitals (HOMO and LUMO) play a significant effect on chemical reactivity, according to frontier molecular orbital theory. The global chemical reactivity parameters of a molecule are all correlated with the HOMO‐LUMO gap.^[30]^ The HOMO‐LUMO gap (E_gap_) for **T‐1‐AFPB** in the present investigation is 4.409 eV, whereas the softness (0.45 eV), Figure 2.C and Table 1, both of which may be contributing factors to its higher chemical activity and polarizability. The narrow HOMO‐LUMO gap is crucial for poor chemical stability because each possible reaction benefits energetically from the addition of electrons to a high‐lying LUMO and/or the removal of electrons from a low‐lying HOMO.^[31]^ The total density of the state plot in Figure 2.D separated HOMO and LUMO by the same E_gap_ value (4.409 eV) and are found to be over unoccupied orbitals above LUMO.

The quantum theory of atoms in molecules (QTAIM) was explored using Multiwfn and AIMALL programs to address the bond nature within the **T‐1‐AFPB**. The results were presented in Figure 2.E, a colored contour map, which showed the slanted placement of the purine cycle to the rest of the compound.

Figure S1 and Table S1 explain, in detail, all bond critical points (BCP), bond paths, and cage critical points (CCP). In Figure 2.F, four important BCPs are presented and their relevant QTAIM parameters such as the electron density function values (ρ), Laplacian values (∇^2^ρ), Hamiltonian kinetic energy (K), Lagrangian kinetic energy (G), potential energy density (V) and total energy density (H) are listed in Table S1. The values of (ρ) for new BCPs are positive and smaller than 0.1 a.u. while the (∇^2^ρ) values are positive as well. Such findings conducted the non‐covalent (closed shell) bonds. The calculated values of (H(r)) are positive suggesting electrostatic non‐covalent bonds. The four BCPs enhances the geometry stability as they formed four new CCPs.

The molecules’ electrostatic potential (MESP) of **T‐1‐AFPB** was evaluated to predict the reactive regions for electrophilic and nucleophilic attack. MESP aids in the interpretation of biological recognition mechanisms and H‐bonding interactions. The negative zones are shown by red, maximum positive areas are represented by blue, and zero potential areas are represented by green. Red represents the region that is a favorable location for electrophilic activity. ESP shows areas of negative, positive, and neutral electrostatic potential as well as molecule shape, size, and color gradation. According to the mapping of MESP, Figure 2.G, regions with negative potential are located over electronegative elements like oxygen, nitrogen, and fluoride atoms, whereas regions with positive potential are located over hydrogen atoms. Green (neutral) regions are ready for hydrophobic interactions (H.I). In Figure 2.G, the oxygen atoms in the **T‐1‐AFPB** have a maximum negative potentiality of −6.2×10^−2^ eV while the hydrogen atoms have the highest positive potentiality of +6.2×10^−2^ eV.

### Molecular docking

**Figure 3:**
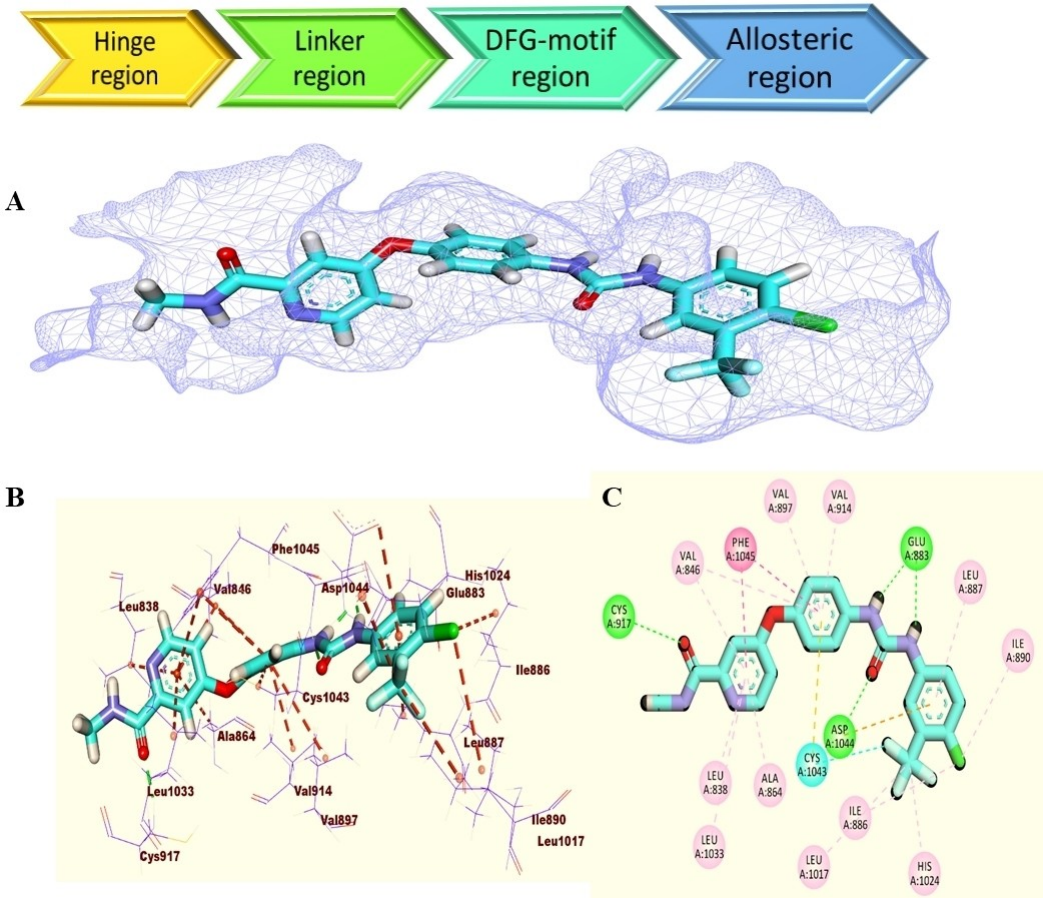
A) Surface mapping representation, B) 3D representation and C) 2D representation of sorafenib inside the VEGFR‐2’s active pocket.

A molecular docking tool can envisage a given molecule‘s ability to bind to a definite molecular target. In this study, **T‐1‐AFPB** was docked against the target enzyme VEGFR‐2. The way that sorafenib, a standard VEGFR‐2 inhibitor, bound to the VEGFR‐2 active site cavity was consistent with what had previously been observed.^[32]^ It resides in the receptor‘s major structural components, including the hinge region (H‐bond with Cys917) and the DFG‐motif region (H‐bond with Glu883 and Asp1044). Additionally, it created two networks of hydrophobic boundaries in the linker region and the terminal hydrophobic regions (Figure 3).

**Figure 4:**
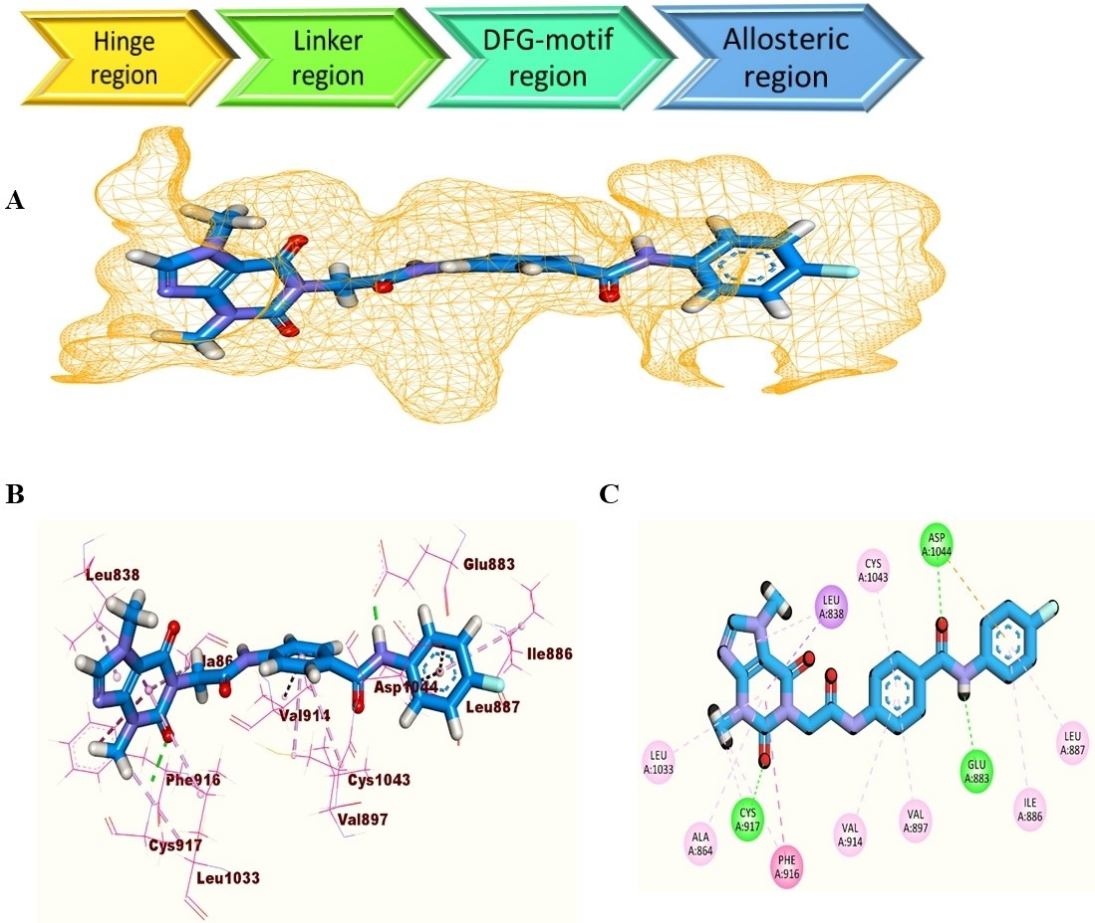
A) Surface mapping representation, B) 3D representation and C) 2D representation of **T‐1‐AFPB** inside the VEGFR‐2’s active pocket.

Likewise, **T‐1‐AFPB** binds to the main four regions of the VEGFR‐2 active pocket. In the hinge region, the purine part interacted *via* its 2‐oxo group and formed a stable H‐bond with the important residue Cys917. As well, three π‐π interactions were observed in the linker region between the phenyl group and Val897, Val914, and Cys1043. The crucial residues Glu883 and Asp1044 in the DFG motif region were connected by two H‐bonds formed by the amide group of **T‐1‐AFPB**. Moreover, **T‐1‐AFPB** successfully occupied the allosteric region and interacted with its hydrophobic backbone (Ile886 and Leu887) *via* the 4‐fluorophenyl part. These findings gave a justification for the powerful VEGFR‐2 inhibitory actions of the **T‐1‐AFPB** (Figure 4).

### Molecular Dynamic (MD) Simulations

**Figure 5:**
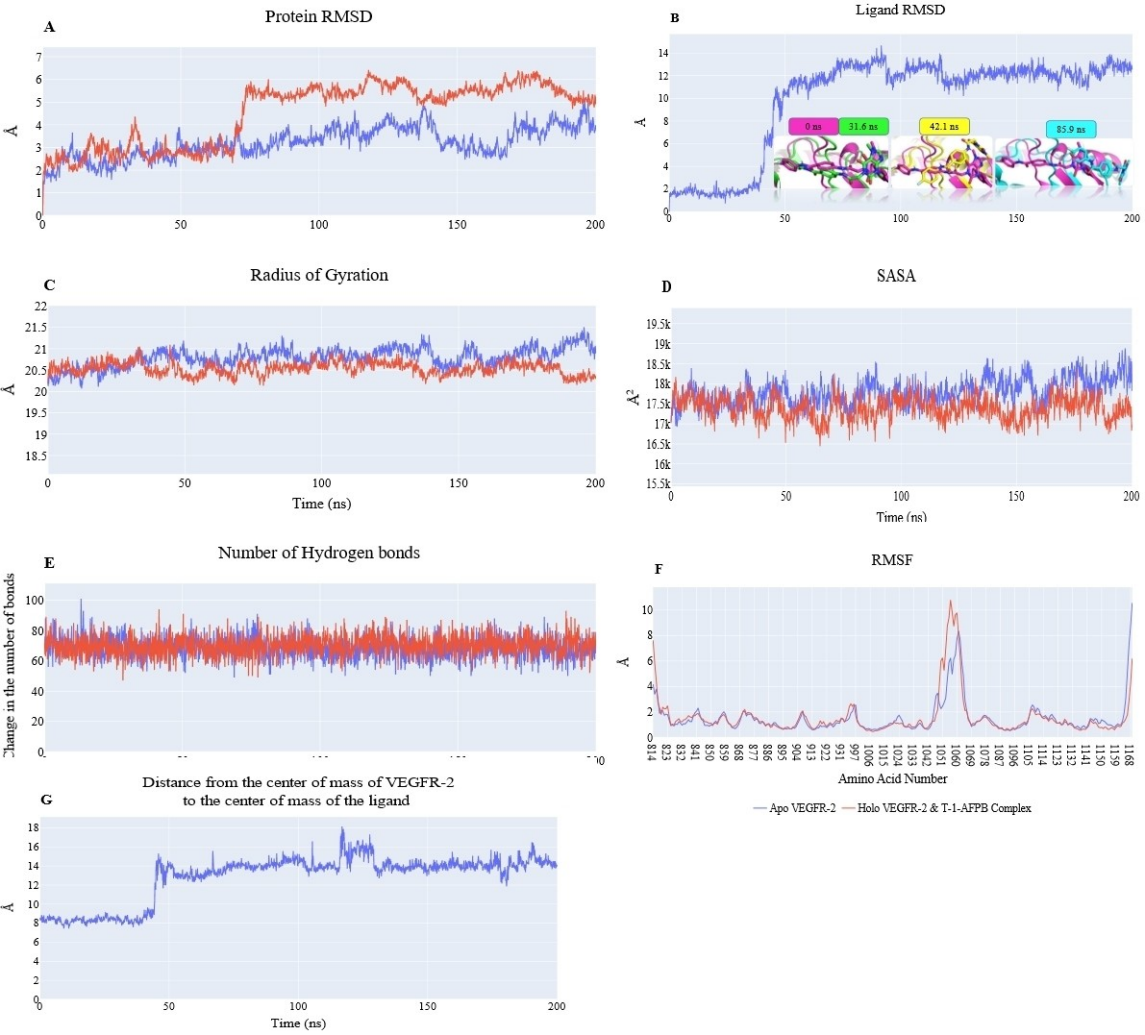
A) RMSD values from the trajectory for the VEGFR‐2, B) **T‐1‐AFPB** RMSD, the insets show the reason for the increase in RMSD. The magenta sticks represent **T‐1‐AFPB** at 0 ns, the green sticks represent **T‐1‐AFPB** at 31.6 ns, the yellow sticks represent **T‐1‐AFPB** at 42.1 ns, and the cyan sticks represent **T‐1‐AFPB** at 85.9 ns, C) RG*, D)* SASA, E) change in the number of H‐bonds G) distance from the center of mass of **T‐1‐AFPB** and VEGFR‐2 protein.

The **T‐1‐AFPB** molecule was shown to exist in two different configurations inside the binding pocket throughout the VEGFR‐2′s production run, with a significant change in the center of the mass distance between **T‐1‐AFPB** and the VEGFR‐2. Two average values could be seen in the Root Mean Square Deviation (RMSD) plots of the holo VEGFR‐2 (red line in Figure 5.A). In the first 70 ns, it has an average value of 2.7 Å, and then it increases to 5.4 Å as the simulation continues. It can be shown by comparing the initial structure to the structure at 80 ns that this is because the Ala1048:Pro1066 loop has opened (Figure 5.H). On the other hand, the apo VEGFR‐2 shows a more stable trend throughout the simulation with an average of 3.2 Å. Similarly, the RMSD of the **T‐1‐AFPB** over the simulation‘s time window shows two states. Figure 5.B shows that during the first 40 ns of the simulation, the RMSD maintains an average of 1.7 Å then it rises to an average of 11.9 Å as a result of conformational changes and the mobility of the ligand. Figure 5.B insets show a comparison of the **T‐1‐AFPB**, at 0 ns (magenta sticks), 31.6 ns (green sticks), 42.1 ns (yellow sticks), and 85.9 ns (cyan sticks). The Radius of Gyration (RG) values exhibit a steady variation, with a mean of 20.7 Å and 20.5 Å, for the apo and holo VEGFR‐2, respectively, as shown in Figure 5.C. Similar trends may be seen in the Solvent Accessible Surface Area (SASA) values, with a mean of 17796 Å^2^ and 17392 Å^2^ for the apo and holo VEGFR‐2, respectively (Figure 5.D). On average, as illustrated in Figure 5.E, there are 70 H‐bonds present between the amino acids for the two proteins. Except for the N‐terminus (4.1 Å and 7.6 Å for the apo and, respectively), the Ala1048:Pro1066 loop (8.3 Å and 10.7 Å for the apo and holo VEGFR‐2, respectively), and the C‐terminus (10.5 Å and 6.2 Å for the apo and holo VEGFR‐2, respectively), the Root‐Mean‐Square Fluctuation (RMSF) plot (Figure 5.F) shows a very little variation (less than 2 Å) among the amino acids. In Figure 5.G, we can see that **T‐1‐AFPB** is separated from the VEGFR‐2′s mass center with two averages. The first one appears in the first 44 ns of the simulation with an average value of 8.3 Å, whereas the second one lasts for the remainder of the simulation and has an average value of 14 Å.

**Figure 6:**
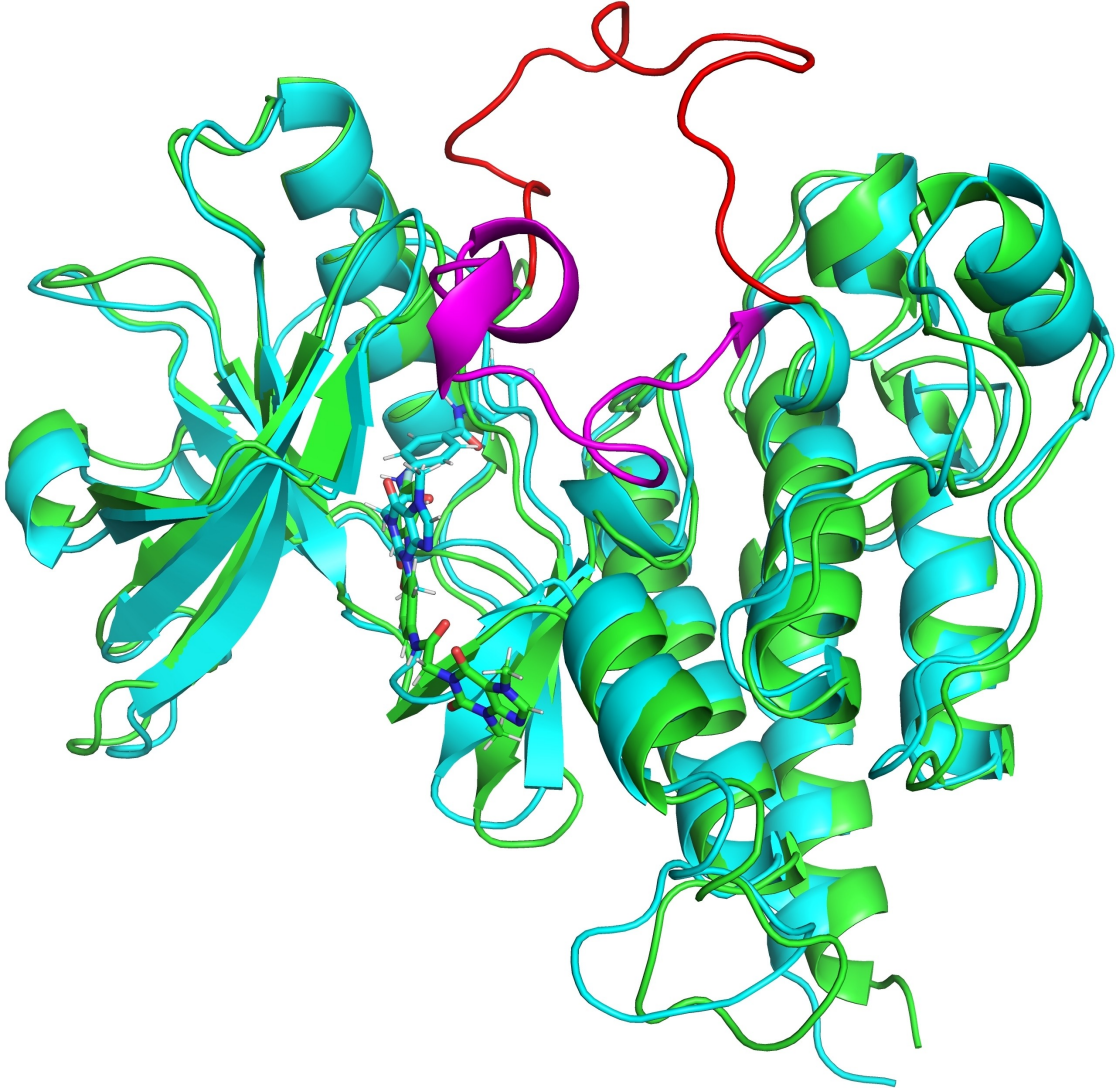
Ala1048 to Ala1066 loop motion at two snapshots at 0 ns (cyan cartoon), and 80 ns (green cartoon). The magenta and red part represent the Ala1048 to Ala1066 loop for 0 and 80 ns snapshots, respectively.

Figure 6 shows the holo VEGFR‐2 at two snapshots (0 ns and 80 ns) which indicate the opening of the Ala1048:Pro1066 loop and might account for the **T‐1‐AFPB**′s mobility.

### Molecular Mechanics with Generalized Born and Surface Area (MM‐GBSA) studies

**Figure 7:**
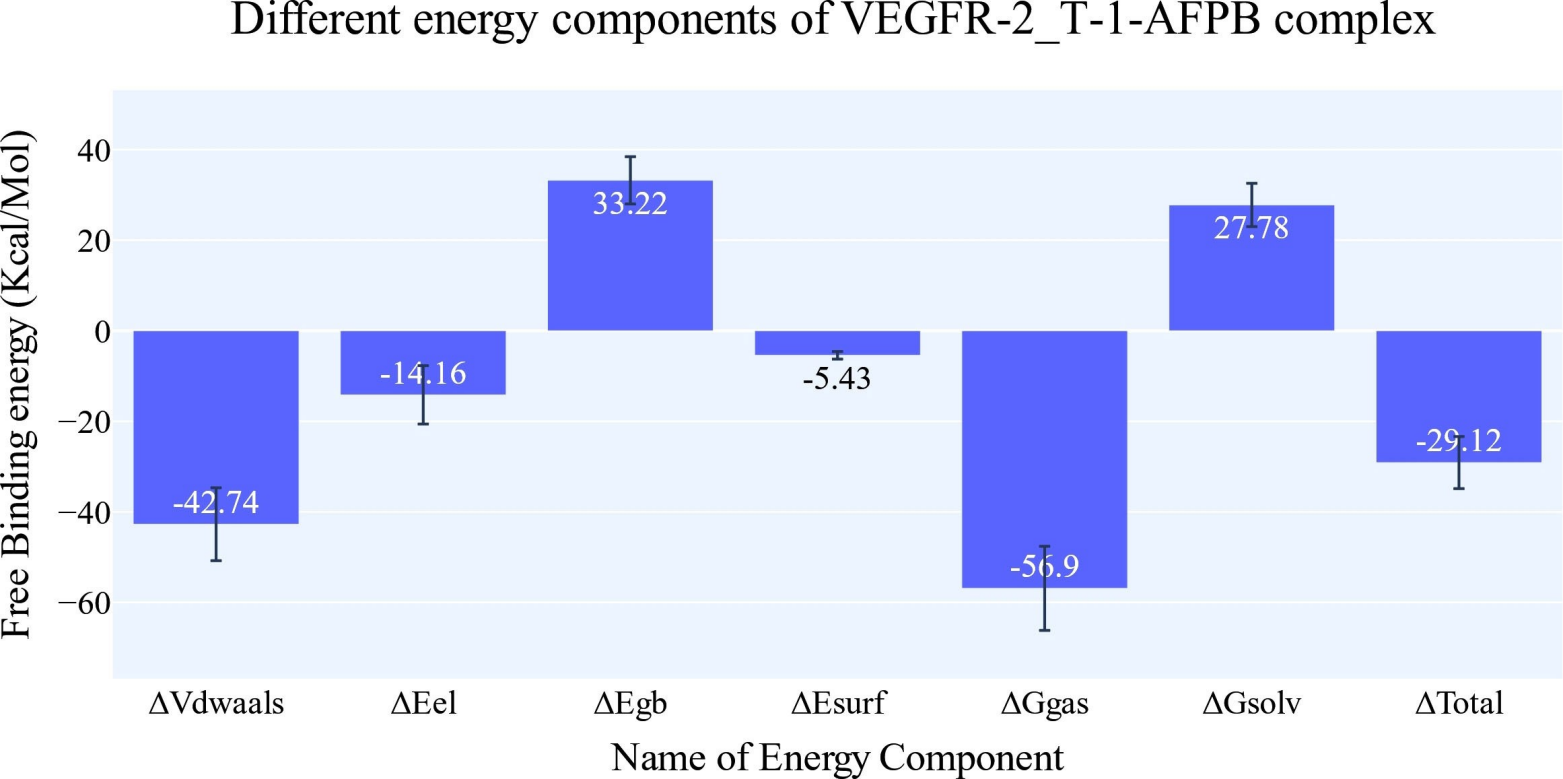
MM‐GBSA of VEGFR‐2_**T‐1‐AFPB** complex showing different energy components and the value of each one. Bars represent the standard deviations (SD) over the 200 ns of the trajectory time.

**Figure 8:**
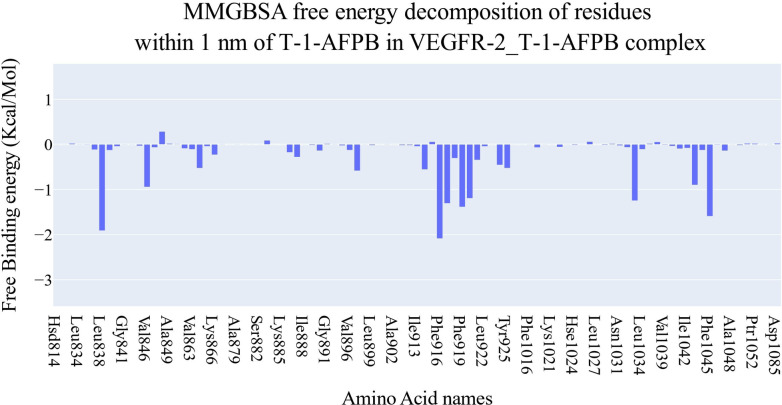
Decomposition of VEGFR‐2_**T‐1‐AFPB**’s binding free energy

Figure 7 displays the different components of the binding free energy for MM‐GBSA. In general, the **T‐1‐AFPB** has a binding energy of −29.12 kcal/mol on average. When comparing the contribution of the energies of various interactions, electrostatic interaction energy is the least effective with an average of −14.16 kcal/mol, while van der Waals energy is the most favorable at around −42.74 kcal/mol. Amino acids within 1 nm of the **T‐1‐AFPB** that were identified by decomposition analysis were shown to contribute to the interaction with binding energy better than −1 kcal/mol (Figure 8). These amino acids are Leu838 (−1.91 kcal/mol), Phe916 (−2.08 kcal/mol), Cys917 (−1.3 kcal/mol), Phe919 (−1.38 kcal/mol), Gly920 (−1.19 kcal/mol), Leu1033 (−1.24 kcal/mol), and Phe1045 (−1.59 kcal/mol).

### Protein‐Ligand Interaction Profiler (PLIP) studies

**Figure 9:**
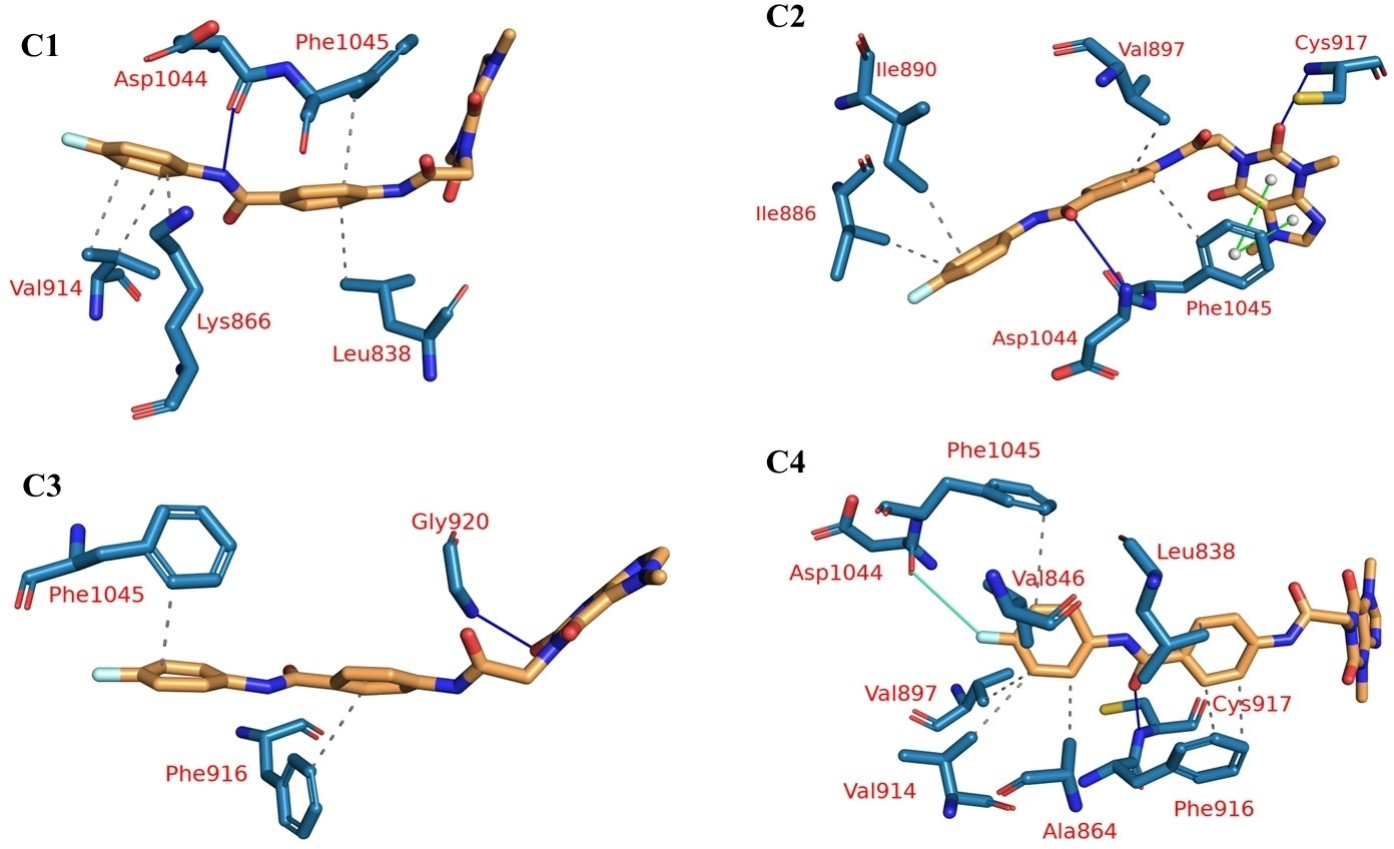
PLIP outputs showing the interactions of **T‐1‐AFPB** (orange sticks) with the amino acids of VEGFR‐2 (blue sticks) through the computed four clusters (C1, C2, C3 and C4). The represented interactions are: H‐bond (blue solid lines), hydrophobic interactions (dashed grey lines), π‐stacking (green dashed lines), and halogen bond (cyan solid lines).

After that, we used TTClust to cluster the trajectory, and for each cluster, a representative frame was obtained. We found four unique clusters by using the elbow technique. All cluster representatives of VEGFR‐2 **T‐1‐AFPB** were used to detect the interactions between the protein and the ligand using the PLIP website. The number and kind of PLIP‐detected interactions are shown in Table 2. At most, three interaction types were detected in the representative frames with numbers of 19 hydrophobic interactions (HIs), 5 H‐bonds, 2 π‐stacking, and 1 halogen bond. The amino acid Phe1045 has the highest number of HIs compared to any other. Moreover, it forms two π‐stacking interactions in the second cluster representative. On the other hand, Asp1044 makes just a single halogen bond in the last cluster. PLIP creates a .pse file that allows one to see the ligand‘s 3D conformation and its interaction with the protein (Figure 9).

**Table 2:** The interactions of VEGFR‐2 and **T‐1‐AFPB** according to the PLIP webserver. Bold amino acids are the amino acids with the largest number of interactions in all cluster representatives.

Cluster number	H.Is	Amino acids	H‐ bond s	Amino acids	π‐ stacking	Amino acids	Halogen bonds	Amino acids
C1	5	Leu838–Lys866–Val914 (2)–**Phe1045**	1	Asp1044	0	None	0	None
C2	4	Ile886–Ile890–Val897–**Phe1045**	2	Cys917–Asp1044	2	Phe1045 (2)	0	None
C3	2	Phe916–**Phe1045**	1	Gly920	0	None	0	None
C4	8	Leu838–Val846–Ala864–Val897–Val914–Phe916 (2)–**Phe1045**	1	Cys917	0	None	1	Asp1044

### Principle Component Analysis (PCA) analysis

**Figure 10:**
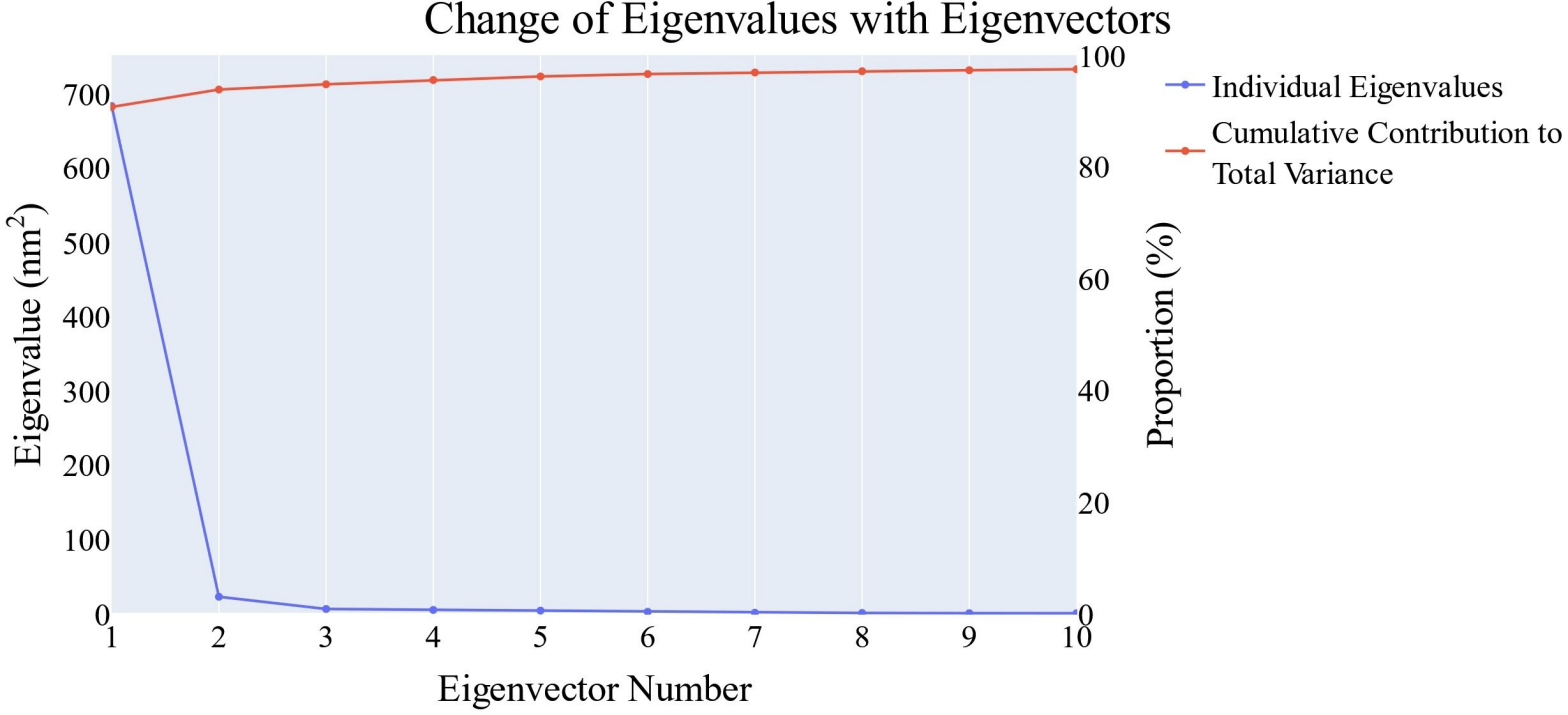
The change in the eigenvalues.

**Figure 11:**
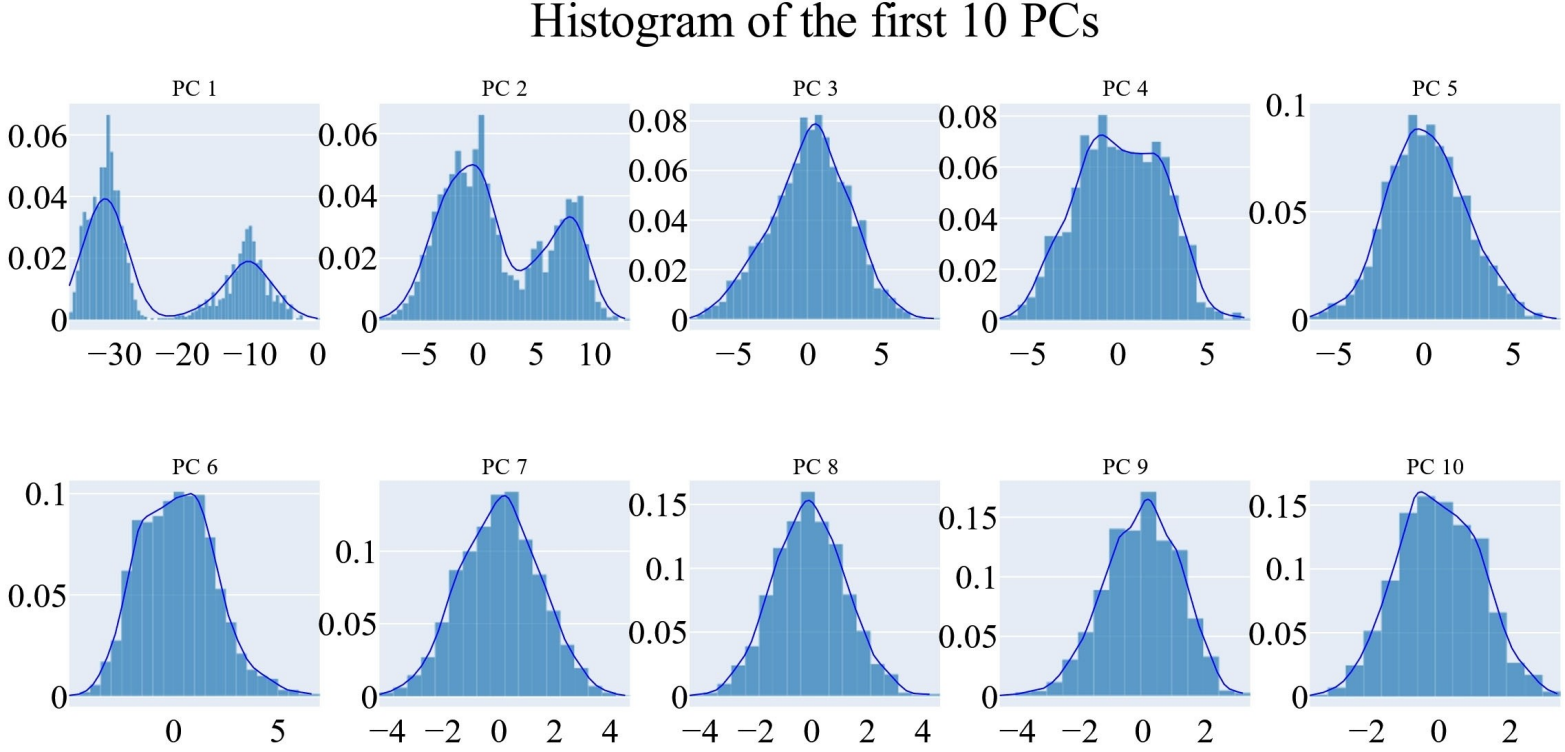
The distribution of the first ten eigenvectors.

The origin of the trajectory‘s synchronized motion was determined using principal component analysis. As shown in the methodology section, the essential subspace was chosen using the scree plot, the eigenvector distribution, and the variance maintained with additional eigenvectors. As can be seen from the scree plot, the slope reduces noticeably after the second PC. Almost 94.7 % of the total variance was maintained by the top three eigenvectors, and almost 91 % was maintained by the first eigenvector alone (Figure 10). The first four eigenvectors have been demonstrated to not have a Gaussian distribution (Figure 11). Thus, the top three eigenvectors were chosen to represent the essential subspace.

**Figure 12:**
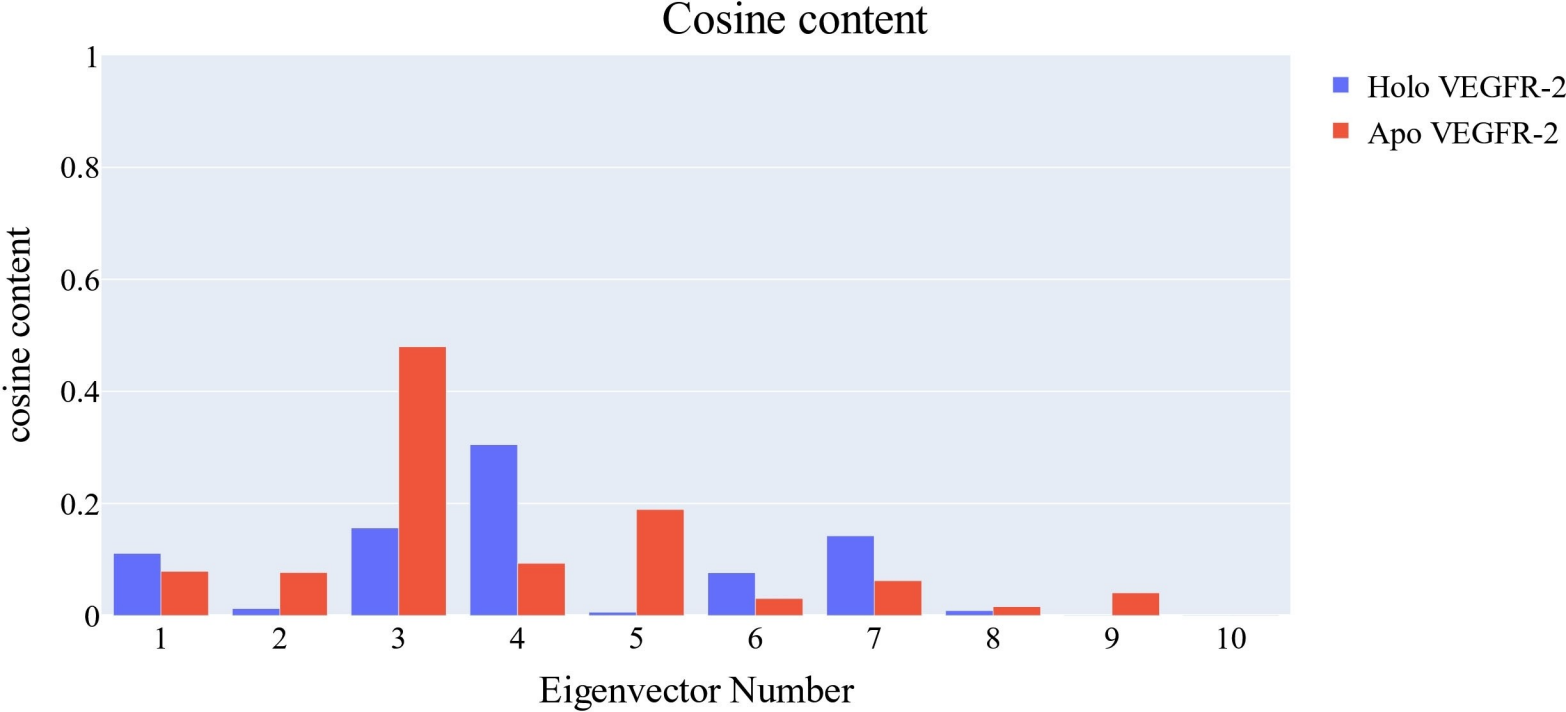
The cosine content of the first ten eigenvectors for the two trajectories.

To evaluate the randomness of the motion reflected by the first 10 eigenvectors, the cosine content was calculated for both apo and holo VEGFR‐2 simulations. Except for the third eigenvector of the apo VEGFR‐2, which has a value of 0.47, the cosine content of the first 10 eigenvectors of both the apo and holo VEGFR‐2 is less than 0.4. (Figure 12). Due to the little amount of overlap between the first three eigenvectors (22.7 % as calculated by the Root Mean Square Inner Product (RMSIP)), it is clear that the two trajectories were sampled differently. The RMSIP also found that the apo and holo C matrices were only 30.6 % similar.

### Bidimensional projection analysis

**Figure 13:**
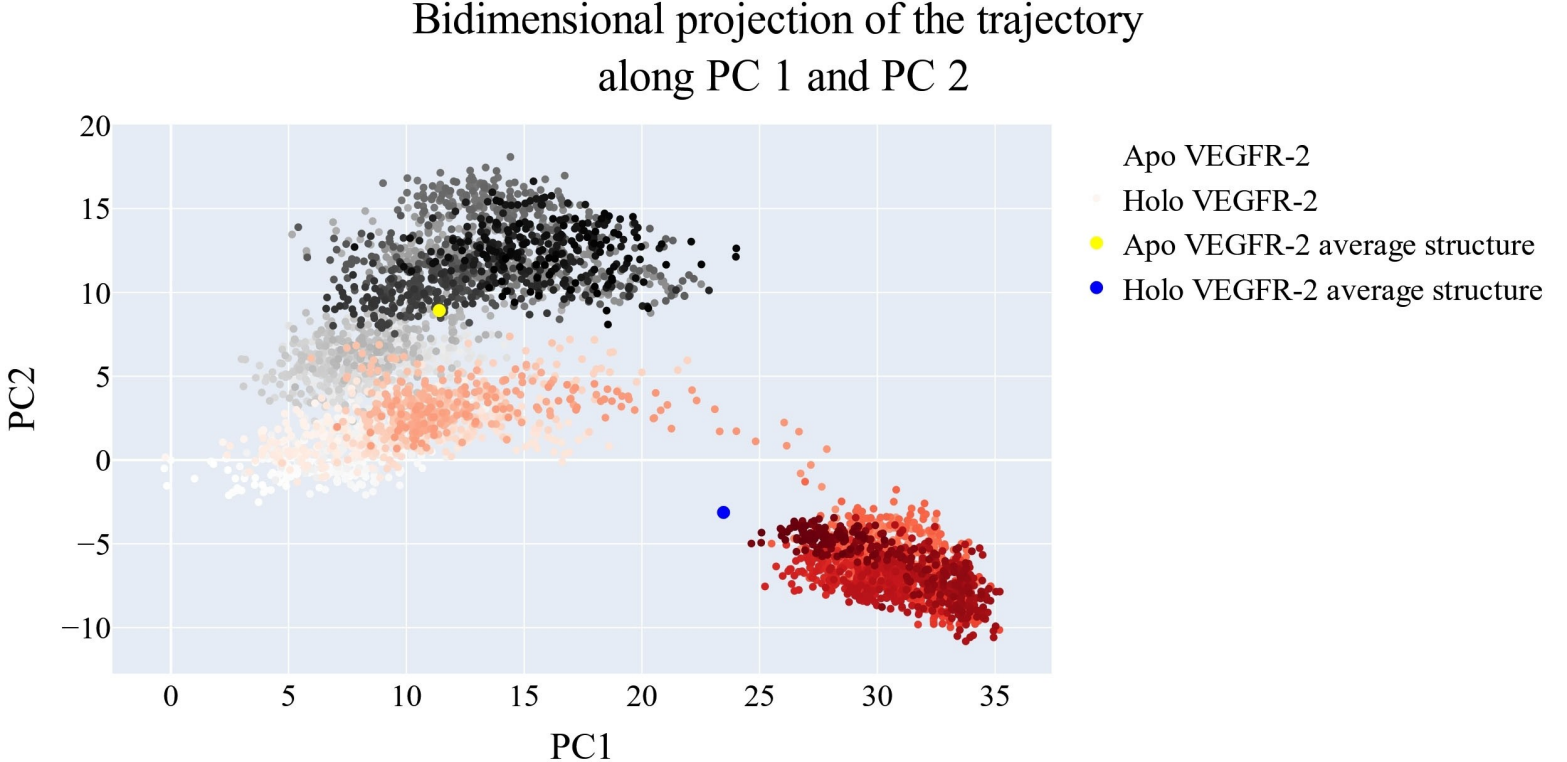
The projection of each trajectory on the first two eigenvectors.

**Figure 14:**
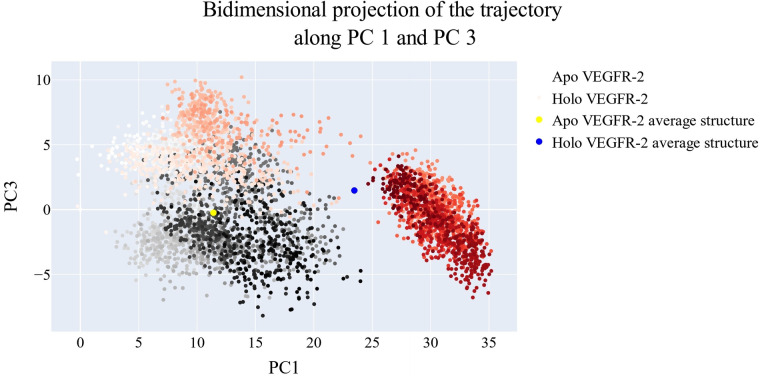
The projection of each trajectory on the first and third eigenvectors.

**Figure 15:**
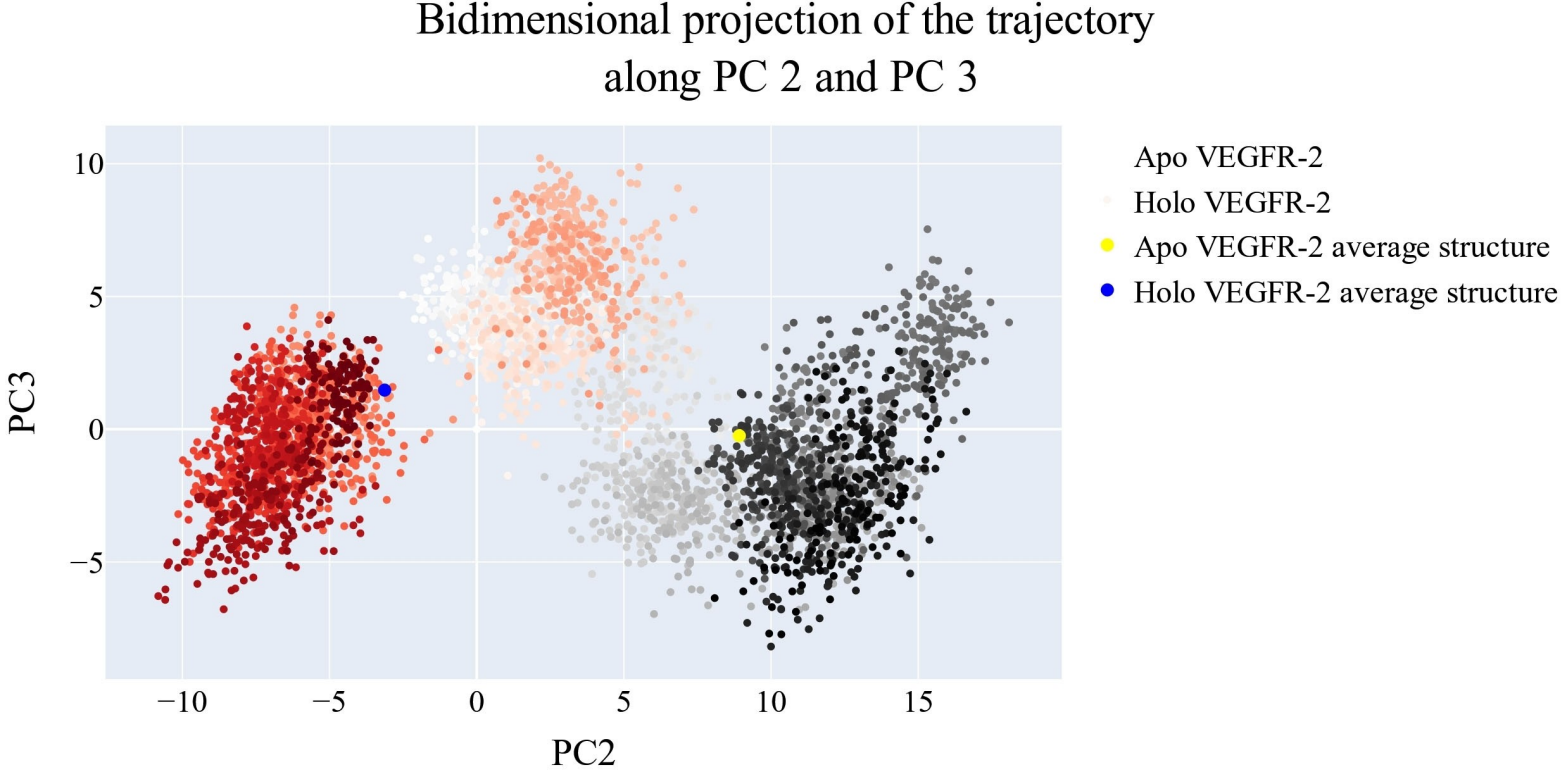
the projection of each trajectory on the second and third eigenvectors.

The projections of each trajectory onto the first three eigenvectors of the whole C matrix are shown in Figures 13, 14–15. The huge dot in each graph is symbolic of the average structure of the corresponding trajectory. Figure 13 (a projection of the first two eigenvectors) demonstrates that the two trajectories are distinct and initially slightly overlap (pale grey and pale red dots) then the two simulations become different in sampling (dark grey and red dots). There is a lot of overlap in the first few frames of the trajectories projected onto the first and third eigenvectors, as shown in Figure 14. Finally, a projection on the second and third eigenvectors reveals in Figure 15 that there is almost little overlap between the two trajectories and that their average structures are entirely distinct.

**Figure 16:**
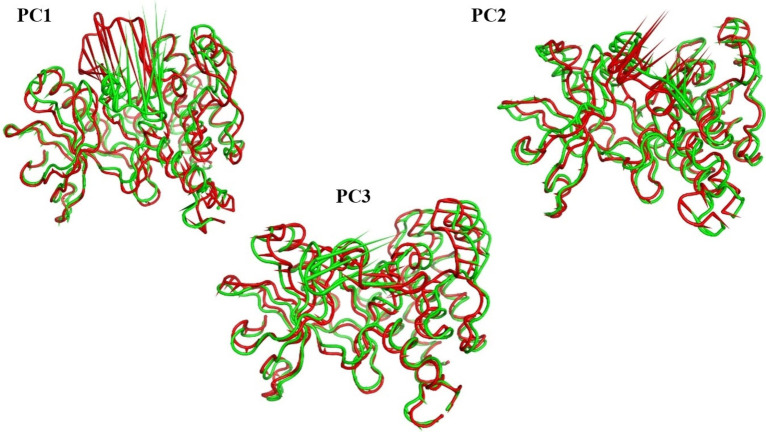
The porcupine figures of each of the first three eigenvectors for both systems. green cartoon: apo VEGFR‐2 trajectory, red cartoon: holo VEGFR‐2 trajectory.

The first three eigenvectors′ motion was shown using porcupine diagrams (Figure 16). The Gly1046:Leu1065 loop motion was found to be the most captured motion of the three eigenvectors. Loop opening in the apo VEGFR‐2 (green cartoon) and loop closing in the holo VEGFR‐2 (red cartoon) are characterized by the first eigenvector. Both the apo and holo VEGFR‐2 exhibit the loop opening, as seen by the second eigenvector. Both trajectories have opposite loop motion in the third eigenvector. In the apo VEGFR‐2, it opens, while in the holo VEGFR‐2, it slightly closes.

### Absorption, Distribution, Metabolism, Excretion and Toxicity (ADMET) profiling study

The approval of a new drug is a complex process that involves evaluating its pharmacokinetic properties and biological activity.^[33]^ It is important to assess the pharmacokinetic features of a new compound early in the drug discovery process to avoid delays in approval or even withdrawal from the market.^[34]^ Therefore, in order to establish the ADMET parameters for **T‐1‐AFPB** against sorafenib, the Discovery Studio 4.0 computing ADMET parameters were used.

**Figure 17:**
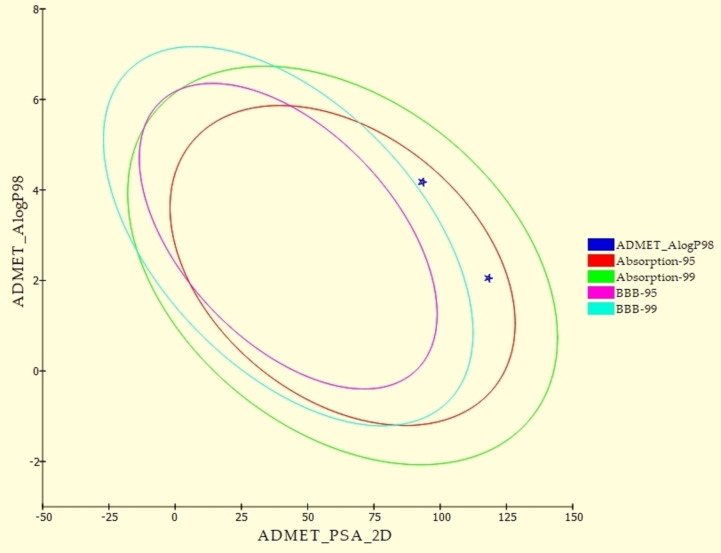
Computational prediction of ADMET parameters for **T‐1‐AFPB** and Sorafenib.

Interestingly, the ADMET results of **T‐1‐AFPB** compared to sorafenib (Figure 17 and Table 3) indicated a higher level of resemblance. It was projected to have a poor ability to cross the blood‐brain barrier (BBB), which can be a limiting factor for drugs targeting the central nervous system. Additionally, CYP2D6 inhibition was anticipated to be absent, which is important to avoid potential drug‐drug interactions. The ADMET calculations also showed good levels of aqueous solubility and intestinal absorption for **T‐1‐AFPB**, which are important factors for oral drug delivery. Furthermore, **T‐1‐AFPB** demonstrated a plasma protein binding (PPB) ability of less than 90 %, suggesting that it is less likely to be bound to plasma proteins and may be more freely available to exert its therapeutic effects. In conclusion, the ADMET results of **T‐1‐AFPB** demonstrate promising features for further development and clinical trials. However, additional studies are necessary to confirm its safety and efficacy in humans.

**Table 3:** ADMET parameters for **T‐1‐AFPB** and sorafenib.

Comp.	BBB level	Solubility level	Absorption level	Hepatotoxic prediction	CYP2D6 prediction	Plasma protein binding
**T‐1‐AFPB**	Very low	Good	Good	Non toxic	No inhibition	<90 %
Sorafenib	Very low	Low	Good	Toxic	No inhibition	>90 %

### In silico toxicity studies

To minimize drug approval failures, toxicity estimation in the early stages of drug development is crucial.^[35]^ However, traditional *in vitro* and *in vivo* research methods can be expensive, time‐consuming, and ethically restricted. Therefore, the use of *in silico* techniques in toxicity prediction has become increasingly popular.^[36]^ In this study, the toxicity of **T‐1‐AFPB** was estimated using eight toxicity models in the Discovery studio program and compared to Sorafenib. The results showed that **T‐1‐AFPB** had positive and safe values in all of the models run (Table 4). Particularly, in rat oral LD_50_ and chronic LOAEL models, **T‐1‐AFPB** was much safer than sorafenib.

**Table 4:** *In silico* toxicity studies of **T‐1‐AFPB** and sorafenib.

Toxicity model	**T‐1‐AFPB**	Sorafenib
Ames mutagenicity prediction	Non‐Mutagen	Non‐Mutagen
FDA Rodent Carcinogenicity (Female‐rats)	Non‐Carcinogen	Non‐Carcinogen
Carcinogenic Potency TD_50_ (Mouse)	9.975 mg/kg/day	19.236 mg/kg/day
Rat Maximum Tolerated Feeding Dose	0.047 g/kg	0.089 g/kg
Rat Oral LD_50_	2.050 g/kg	0.823 g/kg
Rat Chronic LOAEL	0.014 g/kg	0.005 g/kg
Ocular irritation potential	Mild	Mild
Dermal irritation potential	None irritant	None irritant

### Chemistry

**Scheme 1:**
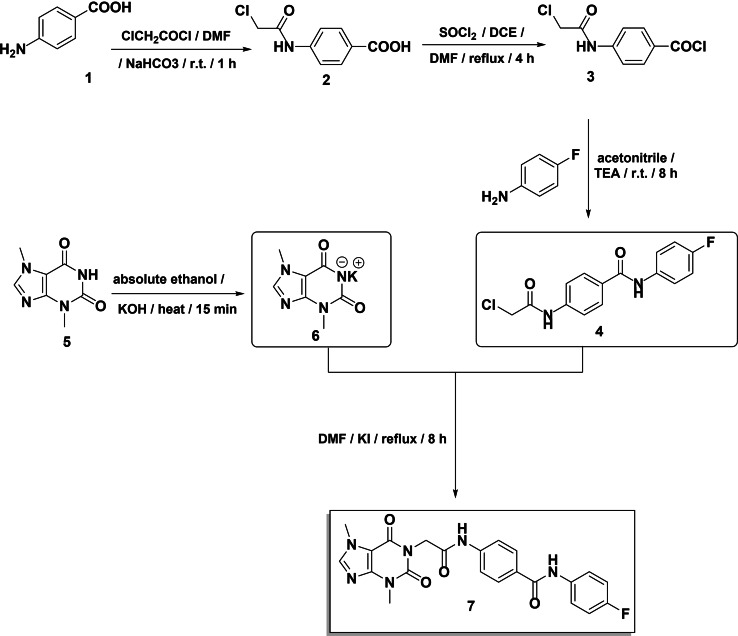
Semi‐synthetic pathway of **T‐1‐AFPB**.

The aforementioned promising outcomes of the *in silico* studies of **T‐1‐AFPB** motivated us to synthesize it as presented in Scheme 1. The chloroacetamide derivative **2** ^[37]^ was produced by acetylating *p*‐aminobenzoic acid **1** with chloroacetyl chloride in DMF. A carboxylic acid derivative **2** reacted with SOCl_2_ to produce the corresponding acyl chloride **3**.^[37]^ Mixing of the highly reactive acid chloride **3** with 4‐fluoroaniline in acetonitrile in the presence of triethylamine produced the target intermediate 4‐(2‐chloroacetamido)‐*N*‐(4‐fluorophenyl)benzamide **4** ^[38]^ in a good yield. On the other hand, to produce potassium salt **6**,^[39]^ theobromine **5** was treated with alcoholic KOH while being stirred continuously. The formed potassium salt **6** was next refluxed with 4‐(2‐chloroacetamido)‐*N*‐(4‐fluorophenyl)benzamide **4** in DMF using a catalytic amount of KI to afford the final **T‐1‐AFPB** (Scheme 1).

IR of **T‐1‐AFPB** was characterized by the appearance of carbonyl absorption bands at 1677 cm^−1^. Regarding the ^1^H NMR spectrum, one characteristic signal for methylene protons appeared at 4.75 ppm. Additionally, at 10.60 and 10.21 ppm, two downfield singlet signals pertaining to the two amidic protons have been detected. The ^13^C NMR spectrum, which displayed distinct peaks at 43.98, 33.67, and 29.92 ppm, corresponding to the CH_2_ and the 2 CH_3_ groups, supported the validity of the proposed structure.

### In vitro VEGFR‐2 Inhibition

**T‐1‐AFPB** was specifically designed to act as an inhibitor for VEGFR‐2, which is the primary regulator of angiogenesis. Upon observing its promising inhibitory capabilities through computer simulations, we decided to further investigate the potential of **T‐1‐AFPB** in inhibiting the VEGFR‐2 protein in vitro. Interestingly, the results of the in vitro study demonstrated that **T‐1‐AFPB** was a strong inhibitor of the VEGFR‐2 protein, with an IC_50_ value of 69±3 nM. This value was compared to sorafenib‘s IC_50_ value of 59±3 nM. The in vitro findings were consistent with the promising computational results, indicating that **T‐1‐AFPB** has strong potential as a VEGFR‐2 suppressant compound.

### Cytotoxicity and safety

**T‐1‐AFPB** has demonstrated noteworthy anti‐VEGFR‐2 potential in both in silico and in vitro studies, thus garnering interest as a potential therapeutic agent in cancer treatment. In order to further evaluate its anticancer properties, the cytotoxicity of **T‐1‐AFPB** was assessed against HepG2 and MCF7 cell lines in vitro using sorafenib as a reference drug. The results indicated that **T‐1‐AFPB** exhibited effective cytotoxicity against both cancer cell lines, as evidenced by IC_50_ values of 2.24 μM and 3.26 μM, respectively. These values were very near to those of sorafenib (Table 5). The selectivity index (SI) is a metric employed to assess the safety and effectiveness of a compound. It involves comparing the cytotoxicity of compound in two distinct cell types. The formula for computing the SI is SI=(IC_50_ in a normal cell)/(IC_50_ in a malignant cell). Interestingly, the selectivity index values of **T‐1‐AFPB** against HepG2 and MCF7 cell lines were calculated to be 42.3 and 29.1, respectively, based on the IC_50_ value of 94.82 μM in vero cell lines. Overall, these findings suggest that **T‐1‐AFPB** has a promising potential as a safe anticancer compound and warrants further investigation.

**Table 5:** *In vitro* anti‐proliferative assessment of **T‐1‐AFPB** against HepG2 and MCF7 cell lines.

Comp.	*In Vitro* IC_50_ (μM)^[a]^	
	MCF7	HepG2
**T‐1‐AFPB**	3.26±0.02	2.24±0.02
Sorafenib	3.17±0.01	2.24±0.06

[a] Data are presented as three times values mean of IC_50_.

### Apoptosis assay

**Figure 18:**
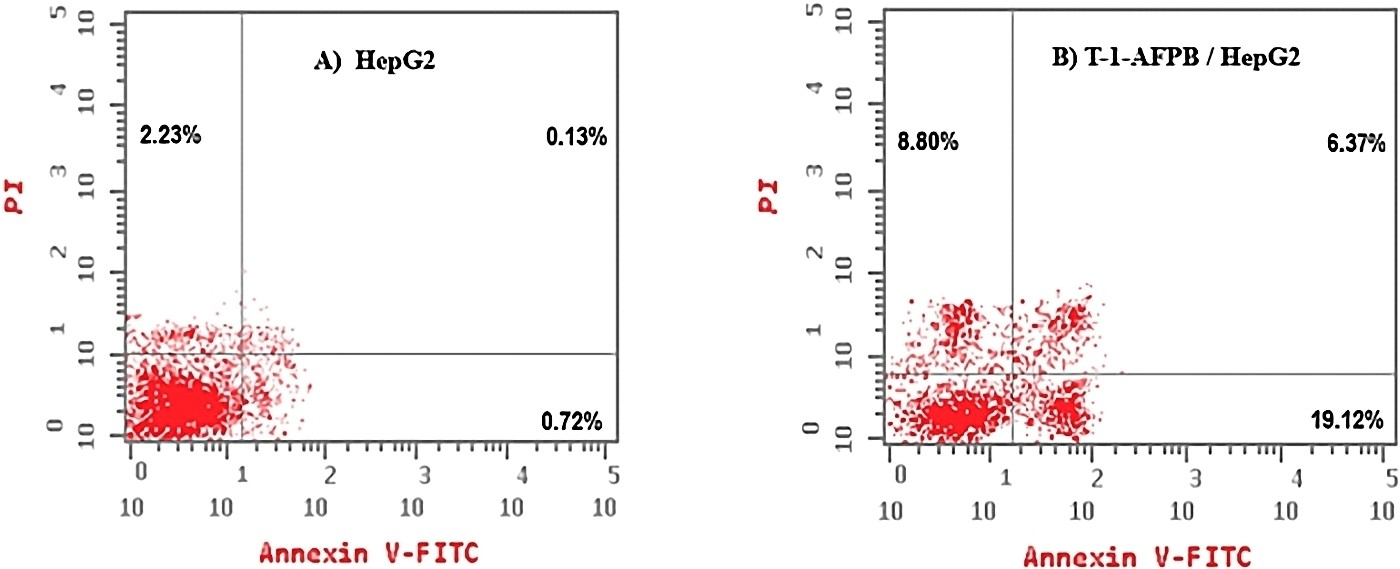
Flow cytometric chart of apoptosis in A) HepG2 cells and B) HepG2 cells exposed to **T‐1‐AFPB**.

Flow cytometry assay was used to investigate the apoptotic effects of **T‐1‐AFPB** on HepG2 cells. The Annexin V and PI double stains were utilized for the assay.^[40]^ As shown in Table 6 and Figure 18, **T‐1‐AFPB** induced a significant increase in the percentage of HepG2 cells in early‐stage apoptosis (from 0.72 % to 19.12 %) and late‐stage apoptosis (from 0.13 % to 6.37 %) when compared to the control group. The percentage of necrosis in the cells treated with **T‐1‐AFPB** was four times higher (8.80 %) than that of the control cells (2.23 %). These findings suggest that **T‐1‐AFPB** has cytotoxic potential and may be associated with inducing apoptosis, leading to cell cycle arrest in HepG2 cells.

**Table 6:** Effect of **T‐1‐AFPB** on stages of the cell death process in HepG2 cells.

Comp.	Apoptosis			Necrosis (%)
	Total (%)	Early (%)	Late (%)	
**T‐1‐AFPB**	34.29	19.12	6.37	8.80
Control	3.04	0.72	0.13	2.23

## Conclusions

This study reports on the development of a novel VEGFR‐2 inhibitor, **T‐1‐APFPB**, which was designed using a CADD approach in line with the pharmacophoric features of VEGFR‐2 inhibitors. The stability and reactivity of **T‐1‐AFPB** were evaluated using DFT calculations. The molecular docking assessments revealed that **T‐1‐AFPB** had the potential to effectively bind to and inhibit VEGFR‐2. The precise binding of **T‐1‐AFPB** to VEGFR‐2 was further confirmed through multiple molecular dynamics (MD) simulations, PLIP, MM‐GBSA, and PCA studies. **T‐1‐AFPB** was then semi‐synthesized, and it′s *in vitro* assays were conducted to evaluate its potential as a VEGFR‐2 inhibitor. The results indicated that **T‐1‐AFPB** could inhibit VEGFR‐2 with a comparable IC_50_ value to that of the standard inhibitor, sorafenib. Additionally, **T‐1‐AFPB** exhibited the ability to suppress the growth of HepG2 and MCF‐7 cancer cell lines with IC_50_ values of 2.24±0.02 and 3.26±0.02 μM, respectively. Importantly, **T‐1‐AFPB** displayed very high selectivity indices against the normal Vero cell lines, indicating its potential as a safe cancer therapeutic. Furthermore, **T‐1‐AFPB** was found to increase the incidence of apoptosis in HepG2 cell lines. The combined computational and experimental approaches utilized in this study provide evidence that **T‐1‐AFPB** can serve as a promising lead VEGFR‐2 inhibitor for further development in cancer therapy.

## Experimental Section

### Docking studies

MOE2014 software was operated to evaluate **T‐1‐AFPB**’s binding to the VEGFR‐2 that has the PDB ID, 2OH4, and a resolution of 2.05 Å for by.^[41]^ A comprehensive elucidation (Protein's preparation, ligands’ preparation, docking setup, validation and proceeding) is incorporated in the Supporting Information.

### MD simulations Studies

Interaction strength and stability of theVEGFR‐2_ **T‐1‐AFPB** complex were evaluated by running a 200‐ns classical unbiased MD simulation in GROMACS 2021, aiming to make a comparison between the apo and holo VEGFR‐2 protein structures.^[42]^ To facilitate this, we utilized the solution builder of the CHARMM‐GUI web server, which aided in preparing the necessary input files for the simulation.^[43]^ A comprehensive elucidation is incorporated in the Supporting Information.

### Binding free energy calculation using MM‐GBSA:

The MM‐GBSA method available in the gmx_MMPBSA software was employed to assess the binding strength of the system. Additionally, we performed decomposition analysis to determine the individual contribution of amino acids within a 1 nm radius of the ligand to the overall binding energy.^[44]^ A comprehensive elucidation is incorporated in the Supporting Information.

### PCA Analysis:

PCA of the mass‐weighted covariance matrix (C) of atoms reveals correlated mobility patterns along the molecular dynamics (MD) trajectories. Specifically, PCA was applied to detect the motion of alpha‐carbons within the range of amino acids Glu826 to Leu1161.^[45]^ A comprehensive elucidation is incorporated in the Supporting Information.

### Bidimensional Projections Analysis

In order to directly compare the frames within the reduced subspace, we followed a series of steps. Firstly, we merged the trajectories of the apo‐protein and complex. Next, we aligned them to the apo‐protein configuration we obtained during equilibration. Then, we created a new C matrix for the merged trajectories. Finally, we projected each trajectory onto the new C matrix. To assess the similarity between the two trajectories, we plotted the projection onto the first three eigenvectors using various combinations of eigenvector pairs.^[46]^

### DFT

Density Functional Theory (DFT) calculations were executed using Gaussian09 W.01D software with the “B3LYP/6‐311G++(d,p)” level of theory. For the assessment of reactivity indices, electrostatic surface potential (ESP), and total electron density (TDOS), we employed GaussView5 and GaussSum 3.0 software.^[47]^ The utilization of these tools facilitated the acquisition of valuable insights into the reactivity and electronic properties of **T‐1‐AFPB**, which played a pivotal role in enhancing the depth of our study. A comprehensive elucidation is incorporated in the Supporting Information.

### ADMET studies

ADMET studies were operated for **T‐1‐AFPB** by Discovery Studio 4.0.^[48]^ A comprehensive elucidation is incorporated in the Supporting Information.

### Toxicity studies

Toxicity studies (eight modules) were operated for **T‐1‐AFPB** by Discovery Studio 4.0.^[49]^ A comprehensive elucidation is incorporated in the Supporting Information.

### General procedure for the semi‐synthesis of T‐1‐AFPB

4‐(2‐Chloroacetamido)‐*N*‐(4‐fluorophenyl)benzamide **4** (0.001 mol) was added to a solution of the potassium salt of 3,7‐dimethyl‐3,7‐dihydro‐1*H*‐purine‐2,6‐dione **6** (0.001 mol) in dry DMF (10 mL), and the mixture was heated in a water bath for 8 h. After being poured onto 200 mL of ice water, the reaction mixture was stirred for 30 minutes. To obtain **T‐1‐AFPB**, the precipitate was filtered, water washed, and crystallized from ethanol.

### 4‐(2‐(3,7‐Dimethyl‐2,6‐dioxo‐2,3,6,7‐tetrahydro‐1H‐purin‐1‐yl)acetamido)‐N‐(4‐fluorophenyl)benzamide


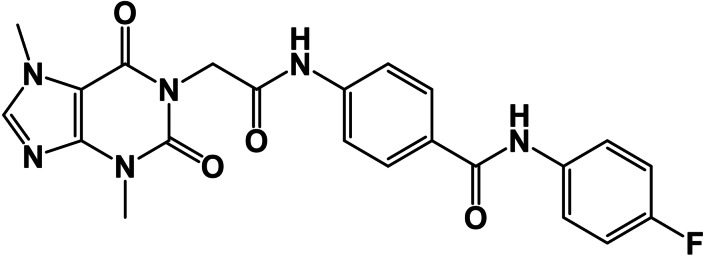


Yellow crystal **(**yield, 8 %); m. p.=240–242 °C; IR (KBr) *ν* cm^−1^: 3275 (NH), 2929 (CH aliphatic), 1711, 1677 (C=O); ^1^H NMR (400 MHz, DMSO‐*d*_6_) δ 10.60 (s, 1H), 10.21 (s, 1H), 8.09 (s, 1H), 7.98 (d, *J*=8.3 Hz, 2H), 7.81 (dd, *J*=8.7, 5.0 Hz, 2H), 7.74 (d, *J*=8.4 Hz, 2H), 7.20 (t, *J*=8.8 Hz, 2H), 4.75 (s, 2H), 3.92 (s, 3H), 3.47 (s, 3H); ^13^C NMR (101 MHz, DMSO‐*d*_6_) δ 166.71, 165.21, 154.63, 151.36, 148.99, 143.75, 142.20, 136.11, 129.68, 129.16, 122.62, 122.55, 118.77, 115.70, 115.48, 107.05, 43.98, 33.67, 29.92; Mass (*m/z*): 450 (M^+^, 17 %), and 201 (100 %, base peak); Anal. calcd. for C_22_H_19_FN_6_O_4_ (450.43): C, 58.66; H, 4.25; N, 18.66; found: C, 58.92; H, 4.37; N, 18.89 %. Moreover, mass spectroscopic analysis for **T‐1‐AFPB** showed a molecular ion peak at 450.

### In vitro VEGFR‐2 inhibition

*In vitro* VEGFR‐2 inhibition was operated for **T‐1‐AFPB** by Human VEGFR ELISA kit. A comprehensive elucidation is incorporated in the Supporting Information.

### In vitro antiproliferative and safety investigations

We employed the MTT assay^[50]^ to assess the cytotoxicity and selectivity of **T‐1‐AFPB** and sorafenib (reference drug) against cancer cell lines (HepG2, MCF‐7) and non‐cancerous cell lines (Vero cell lines). Further comprehensive information and supplementary insights regarding this procedure are available in the Supporting Information.

### Apoptosis analysis

Apoptotic properties of as **T‐1‐AFPB** were evaluated employing a flow cytometry analysis technique.^[51]^ A comprehensive elucidation is incorporated in the Supporting Information.

## Supporting Information Summary

The Supporting Information contains detailed methodologies for (Molecular Docking, MD Simulations, MM‐GBSA, DFT, essential dynamics, ADMET, semi‐synthesis, and the *in vitro* assays). It also includes spectral data (MS, IR, ^1^H and ^13^C NMR), as well as Table S.1 and Figure S.1 illustrating the QTAIM parameters (in atomic units) at bond critical points (BCPs) of **T‐1‐AFPB**. Lastly, a comprehensive computational toxicity report is presented, highlighting the degree of similarity between **T‐1‐AFPB** and a range of reported toxic and safe compounds. **T‐1‐AFPB** is available from the authors.

## Conflict of interest

No conflict of interest to be declared.

1

## Supporting information

As a service to our authors and readers, this journal provides supporting information supplied by the authors. Such materials are peer reviewed and may be re‐organized for online delivery, but are not copy‐edited or typeset. Technical support issues arising from supporting information (other than missing files) should be addressed to the authors.

Supporting Information

## Data Availability

Data are available with corresponding authors upon request.
