# Supplementary material for: A Theobromine Derivative with Anticancer Properties Targeting VEGFR‐2: Semisynthesis, in silico and in vitro Studies
Source: ChemistryOpen. 2023 Oct 6;12(10):e202300066. doi: 10.1002/open.202300066 (PMC10558427; doi:10.1002/open.202300066)
Supplement: Supplementary file 1 — Supporting Information [file OPEN-12-e202300066-s001.pdf]

ChemistryOpen

Supporting Information

A Theobromine Derivative with Anticancer Properties Targeting VEGFR-2: Semisynthesis, *in silico* and *in vitro* Studies

Ibrahim H. Eissa,* Reda G. Yousef, Hazem Elkady, Eslam B. Elkaeed, Aisha A. Alsfouk, Dalal Z. Husein, Ibrahim M. Ibrahim, Mohamed M. Radwan, and Ahmed M. Metwaly*

Content

**Method**	**Molecular Docking**
	**MD Simulations**
	**MM-GBSA**
	**DFT**
	**Essential dynamics studies**
	**ADMET studies**
	**Semi Synthesis**
	***In vitro* assays**
**Spectral Data**	**Ms and IR**
	**^{1}H and ^{13}C NMR**
**Table S.1. QTAIM parameters (a.u.) at bond critical points (BCPs) of T-1-AFPB**	
**Figure S.1. QTAIM analysis: molecular graph showing the bonding critical points (BCP) for T-1-AFPB**	
**Toxicity report**	

Method

- **Molecular Docking studies**

Protein Preparation:

The crystal structure of VEGFR-2 [PDB ID: PDB ID: 2OH4, resolution: 2.05 Å] was obtained from Protein Data Bank (https://www.rcsb.org). At first, the crystal structure of the VEGFR-2 complexed with (sorafenib) the co-crystallized ligand was prepared by removing crystallographic water molecules. Only one chain was retained besides the co-crystallized ligand. The selected protein chain was protonated using the following setting. The used electrostatic functional form was GB/VI with a distance cut-off of 15 Å. The used value of the dielectric constant was 2 with an 80 dielectric constant of the used solvent. The used Van der Waals functional form was 800R3 with a distance cut-off of 10 Å. Then, the energy of the protein chain was minimized using Hamiltonian AM1 implanted in Molecular Operating Environment (MOE 2019 and MMFF94x (Merck molecular force field) for structural optimization. Next, the active site of the target protein was defined for ligand docking and redocking (in case of validation of docking protocol). The active site of the protein was identified as the residues that fall within the 5 Å distance from the perimeter of the co-crystallized ligand.

Ligand Preparation: 2D structures of **T-1-APFPB** and the standard compound, sorafenib, were drawn using ChemBioDraw Ultra 14.0 and saved in MDL-SD file format. The 3D structures of the ligands were protonated, and the structures were optimized by energy minimization using MM2 force-field and 10000 iteration steps of 2 fs. The conformationally optimized ligands were used for docking studies.

Docking Setup and Validation of Docking Protocol: The protein-ligand docking studies were carried out using MOE version 2019. Validation of the docking protocol was carried out by redocking the co-crystallized reference ligand (sorafenib) against the isolated pocket of VEGFR-

2. The docking protocol was validated by comparing the heavy atoms RMSD value of the re-docked ligand pose with the corresponding co-crystallized reference ligand structure.

The docking setup for **T-1-AFPB** was established according to the protocol followed in the validation step. For each docking run, 30 docked solutions were generated using ASE for scoring function and rigid receptor for refinement. The pose with ideal binding mode was selected for further investigations. The docking results were visualized using Discovery Studio (DS) 4.0. Analysis of the docking results was carried out by comparing the interactions and docking score obtained for the docked ligands with that of the re-docked reference molecule (sorafenib).

- **Molecular Dynamic Simulation**

A 200 ns classical unbiased MD simulation was conducted in GROMACS 2021 to evaluate the interaction strength and stability of the VEGFR-2_T-1-AFPB complex and to compare between the apo and holo proteins structures (1). Here, we made use of the CHARMM-GUI web server's solution builder to prepare our input files for simulation (2–5). After uploading a PDB for each system, it was solvated in a cubic box with 1 nm padding using the transferable intermolecular potential 3 points (TIP3P) water model and then neutralized with NaCl ions at a concentration of 0.154 M to approximate physiological salt concentration. Parameters for the VEGFR-2 protein's amino acids, the TIP3P water model, and the neutralizing ions were obtained using the modified Chemistry at Harvard Macromolecular Mechanics (CHARMM36m) force field. To parameterize the T-1-AFPB compound, the CHARMM General Force Field (CGenFF) was utilized.

The dynamics were carried out in GROMACS 2021 with periodic boundary conditions (PBC) applied in all three spatial dimensions. The potential energy of the solvated and neutralized system was reduced to avoid atomic collisions during the production run. Initially, a minimization

step was initiated with a maximum force on any atom fixed at 100 KJ.mol^{-1}.nm^{-1} as a convergence criterion. Then, the pressure and the temperature were equilibrated. The temperature was first brought to equilibrium using a canonical (NVT) ensemble, and then the pressure was brought to equilibrium using an isothermal-isobaric (NPT) ensemble. Both the V-rescale algorithm and the Berendsen barostat were used to get the final results of 310 K for temperature and 1 atm for pressure (6). Nose-Hoover thermostat was used to maintain the temperature at 310 degrees Kelvin during the production run which was started for 200 ns in an NVT ensemble (7). The LINear Constraint Solver (LINCS) was used at each step to set restrictions on the lengths of H-bonded atoms' bonds (8). The electrostatics were calculated using a Particle Mesh Ewald (PME) method with a threshold of 1.2 nm (9). Newtonian equations of motion were integrated using the leap-frog integrator. During equilibration, a time step of 1 femtosecond was used, and during the production run, a time step of 2 femtoseconds was used. During the production process, one frame was recorded every 0.1 ns, for a total of 2000 frames. After removing the PBC from the system using the trjconv tool in GROMACS, the trajectory could be analyzed. To examine the production run, VMD TK scripts were employed (10). The RMSD values of VEGFR-2, and T-1-APFPB were calculated. Root mean square fluctuation (RMSF), the radius of gyration (RoG), solvent accessible surface area (SASA), the distance between ligand and protein center of mass, and the number of hydrogen bonds were also determined. To lower the dimensionality and provide a representative frame for each cluster, the trajectories were clustered using TTClust (11). TTClust used the elbow technique to find the optimal number of clusters after backbone alignment had removed all rotational and translational motion in the system. The protein-ligand interaction profiler (PLIP) was used to detect interactions in each representative frame (12).

Principal Component Analysis:

The principal component analysis (PCA) of the mass-weighted covariance matrix (C) of of atoms indicates correlated mobility along MD trajectories. Alpha-carbon motion in amino acids Glu826:Leu1161 was detected using principal component analysis (12). In the case of single-trajectory analysis, the final frame of the equilibrium step was used as the alignment reference frame. However, the final frame of the apo system after equilibration was used as the reference structure for evaluating the combined trajectories. PCA can figure out which eigenvectors and their corresponding eigenvalues are best at capturing atomic movements by diagonalizing the C matrix. A system's eigenvalue is largest for its first principal component and drops for succeeding PCs, indicating less motion. In GROMACS, we diagonalized the C matrix using gmx covar and analyzed it with gmx anaeig.

When determining the size of the essential subspace, we considered 1) the cumulative sum of the eigenvalues with additional eigenvectors. 2) the location of the largest decrease in the slope of the line between the eigenvalues and eigenvector index number (scree plot). 3) Since it is well-established that non-random eigenvectors do not follow a Gaussian distribution, the distribution of the eigenvectors was also considered.

From the C matrix, we calculated the cosine content (ci) of each eigenvector of the C matrix, which can take on values between 0 (no cosine) and 1 (perfect cosine). The equation of cosine content is as follows:

$$c_i = \frac{2}{T} \left(\int \cos\left(i\pi t\; p_i(t)\; dt\right)^2 \left(\int p_i^2\;(t)\; dt\right)^{-1}$$

Where T is the time of the simulation. Abnormally large ci values, which represent random motion, are related to insufficient sampling. When the cosine content of the first few PCs is near 1, the behavior of proteins on a large scale is analogous to diffusion. Accordingly, the first 10 PCs were used to calculate their cosine content (13–15).

To directly compare the frames in the reduced essential subspace, we aligned the combined apo-protein and complex trajectories to the apo-protein configuration acquired after equilibration, constructed a new C matrix for the combined trajectories, and then projected each trajectory onto the new C matrix. By projecting each trajectory onto the first three eigenvectors with unique eigenvector pair, we were able to assess the degree of similarity between the two trajectories.

Binding free energy calculation using MM-GBSA:

The Molecular Mechanics-Generalized Born Surface Area (MM-GBSA) provided in gmx_MMPBSA was used to evaluate the system's binding strength. We also used decomposition analysis to calculate the contribution of amino acids within 1 nm of the ligand to the binding energy (13,14). The ionic strength was adjusted to 0.154 and the solvation method (igb) was set to 5. All parameters were kept at their defaults except for the dielectric constants, which were altered to 1.0 inside and 78.5 outside. The MM-GBSA method is shown in Equation 1.

$$\Delta G = < G_{complex} - (G_{receptor} + G_{ligand}) > \quad \text{Equation 1}$$

Where < > represents the average of the enclosed free energies of complex, receptor, and ligand over the frames used in the calculation. In our approach, we used the whole trajectory (a total of 2000 frames). Different energy terms can be calculated according to Equations 2 to 6 as follows:

$$\Delta G_{binding} = \Delta H - T\Delta S \quad \text{Equation 2}$$

$$\Delta H = \Delta E_{gas} + \Delta E_{sol} \quad \text{Equation 3}$$

$$\Delta E_{gas} = \Delta E_{ele} + \Delta E_{vdW} \quad \text{Equation 4}$$

$$\Delta E_{solv} = E_{GB} + E_{SA} \quad \text{Equation 5}$$

$$E_{SA} = \gamma.SASA \quad \text{Equation 6}$$

Where:

ΔH is the enthalpy which can be calculated from gas-phase energy (E_{gas}) and solvation-free energy

(E_{sol}). -TΔS is the entropy contribution to the free binding energy. E_{gas} is composed of electrostatic and van der Waals terms; E_{ele}, E_{vdW}, respectively. E_{sol} can be calculated from the polar solvation energy (E_{GB}) and nonpolar solvation energy (E_{SA}) which is estimated from the solvent-accessible surface area (15,16).

- **Density Function Theory (DFT) calculations**

The Gaussian 09 program was used to perform the quantum chemistry calculations for **T-1-AFPB** using the DFT method. GaussianView5 was used to display all of the data files. The density function theory (DFT) at 6-311G++(d,p) basis set/B3LYP approach was utilized to optimize organic chemical structure of the compound under investigation and Chem3D 15.0 software was used to create the original chemical structures. Both the Total Electron Density (TED) and the Electrostatic Surface (ESP) maps were examined at the same theoretical level. GaussSum3.0 software was used to compute and evaluate the total density of state (TDOS) for the optimized log file.

Equations of Koopmans' theory: The chemical potential (μ), ma**xi**mal charge acceptance (ΔN_{max}), global hardness (η), energy change (ΔE), electronegativity (χ), the global softness (σ), electrophilicity index (ω), ionization potential (IP) and electron affinity (EA)

$IP = -E_{\mathrm{HOMO}}$

$EA = -E_{\mathrm{LUMO}}$

μ=

(IP+EA)/2

η= (IP-EA)

χ= - η

$\omega = \mu^2/(2$

$\eta)$ $\sigma = 1/$

η

$\Delta N = -(\mu/\eta)$

$\Delta E = -\omega$

$E_{gap} = E_{LUMO} – E_{HOMO}$

- **Preparation of T-1-AFPB for ADMET and toxicity studies:**

In this protocol, the general-purpose panel was utilized with the activation of the Prepare ligand option. The change ionization was switched on the true option using the Rule based as an ionization method. In Rule based task, we used the carboxylate as an acid ionization. Additionally, the primary, secondary, and tertiary amines were selected as Base ionization. The ionization enumeration option was switched on the one protomer. Under the filter smart option, we selected all options. The false option was selected for tasks Generate tautomers, generate isomers, Fix bad valencies, and parallel processing. The generate coordinates task was switched on the 3D option. Finally, the duplicate structure task was activated on the remove option.

ADMET descriptors (absorption, distribution, metabolism, excretion and toxicity) of **T-1-AFPB** were determined using Discovery studio 4.0. **Sorafenib** was used as a reference molecule. At first, the CHARMM force field was applied then the tested compounds were prepared and minimized according to the preparation of small molecule protocol. The ADMET descriptors that applied including models for

1. Human intestinal absorption,
2. Aqueous solubility,

3. Blood brain barrier penetration,
4. Plasma protein binding,
5. Cytochrome P450 2D6 inhibition, and
6. Hepatotoxicity.

Running of ADMET protocol

In this protocol, after compounds preparation, the small molecules panel was utilized with the activation of the ADMET descriptors option. Then, we selected the prepared compounds as the input ligands. Further, all the ADMET parameters (aqueous solubility, Blood brain barrier, intestinal absorption, CYP2D6, and plasma protein binding) were selected. Then, the output of the running protocol was visualized to give the ADMET chart.

- **Toxicity studies**

The toxicity parameters of **T-1-APFPB** were calculated using Discovery studio 4.0. Sorafenib was used as a reference molecule. Then different parameters were calculated from the toxicity prediction (extensible) protocol (TOPKAT) that evaluated the examined compounds' performance in experimental assays and animal models. TOPKAT computed and validated assessments of the toxic and environmental effects of the examined chemicals solely from their molecular structure. TOPKAT employs robust and cross-validated Quantitative Structure Toxicity Relationship (QSTR) models for assessing various measures of toxicity and utilizing the patented Optimal Predictive Space validation method to assist in interpreting the results.

The predicted models are

1. FDA rat carcinogenicity test,
2. Carcinogenic potentiality TD_{50} (the median toxic dose of a substance in which toxicity occurs in 50% of a species),

3. Maximum tolerated dose (MTD) in rats,
4. Oral LD_{50} in rats (the amount that kills 50% of test animals),
5. Chronic LOAEL (Lowest-observed-adverse-effect level) in rats,
6. Ocular irritancy and
7. Skin irritancy

Running of Toxicity protocol

In this protocol, after compounds preparation, the small molecules panel was utilized with the activation of the toxicity prediction (extensible) option. Then, we selected **T-1-AFPB** and sorafenib as the input ligands. Further, the different toxicity models were selected from the model panel. The similarity search task was activated to be true. The detailed report task was switched on as a PDF file. Then, the output of the running protocol was visualized to give the toxicity PDF report.

- **Chemistry**

General

The melting point were carried out by open capillary method on a Gallen kamp Melting point apparatus. The infrared spectra were recorded on pye Unicam SP 1000 IR spectrophotometer using potassium bromide disc technique. Proton magnetic resonance ^{1}HNMR spectra were recorded on a Bruker 400 Megahertz-nuclear magnetic resonance (400 MHZ-NMR) spectrophotometer. Carbon-13 (C13) nuclear magnetic resonance (^{13}CNMR) spectra were recorded on a Bruker 100 Megahertz-nuclear magnetic resonance (100 MHZ-NMR) spectrophotometer. Tetramethylsilane (TMS) was used as internal standard and chemical shifts were measured in δ scale one part per million (ppm). The reactions were monitored by thin-layer chromatography (TLC) using TLC sheets precoated with UV fluorescent silica gel Merck

60 F254 plates and were visualized using ultraviolet (UV) lamp and different solvents as mobile phases.

General procedure for the semi-synthesis of T-1-AFPB

4-(2-Chloroacetamido)-*N*-(4-fluorophenyl)benzamide **4** (0.001 mol) was added to a solution of the potassium salt of 3,7-dimethyl-3,7-dihydro-1*H*-purine-2,6-dione **6** (0.001 mol) in dry DMF (10 mL), and the mixture was heated in a water bath for 8 hours. After being poured onto 200 mL of ice water, the reaction mixture was stirred for 30 minutes. To obtain **T-1-AFPB**, the precipitate was filtered, water washed, and crystallized from ethanol.

All solvents that were used to the semisynthesis were obtained (analytical grade) from Al-Nasr company, Cairo, Egypt.

4-(2-(3,7-Dimethyl-2,6-dioxo-2,3,6,7-tetrahydro-1*H*-purin-1-yl)acetamido)-*N*-(4-fluorophenyl)benzamide

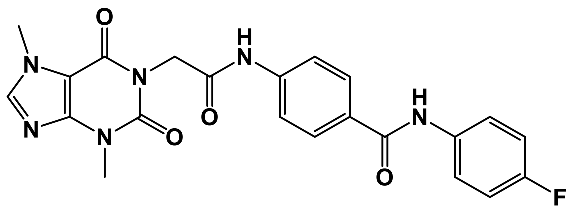

Yellow crystal (yield, 87 %); m. p. = 240-242 °C; IR (KBr) *ν* cm^{-1}: 3275 (NH), 2929 (CH aliphatic), 1711, 1677 (C=O); ^{1}H NMR (400 MHz, DMSO-d_6) δ 10.60 (s, 1H), 10.21 (s, 1H), 8.09 (s, 1H), 7.98 (d, *J* = 8.3 Hz, 2H), 7.81 (dd, *J* = 8.7, 5.0 Hz, 2H), 7.74 (d, *J* = 8.4 Hz, 2H), 7.20 (t, *J* = 8.8 Hz, 2H), 4.75 (s, 2H), 3.92 (s, 3H), 3.47 (s, 3H); ^{13}C NMR (101 MHz, DMSO-d_6) δ 166.71, 165.21, 154.63, 151.36, 148.99, 143.75, 142.20, 136.11, 129.68, 129.16, 122.62, 122.55, 118.77, 115.70, 115.48, 107.05, 43.98, 33.67, 29.92; Mass (*m/z*): 450 (M^+, 17%), and 201 (100 %, base peak); Anal. Calcd. for $C_{22}H_{19}FN_6O_4$ (450.43): C, 58.66; H, 4.25; N, 18.66; Found: C, 58.92; H, 4.37; N, 18.89 %. Moreover, mass spectroscopic analysis for **T-1-AFPB** showed a molecular ion peak at

450.

- **Biological evaluations**

In vitro VEGFR-2 kinase assay

T-1-AFPB was estimated for their *in vitro* inhibition on human VEGFR-2; using ELISA kit. Firstly, a plate was used for the assay had been coated by an antibody specific for human VEGFR-2 enzyme, Sorafenib was nominated as a standard VEGFR-2 inhibitor. Both standard and sample were added to the wells and incubated overnight at 4 ◦C, then washed. The biotinylated antibody was supplemented and further incubated for 1 h at room temperature. The unreacted, liberated antibody was then washed; followed by addition of HRP-conjugated streptavidin and incubated for 45 min at room temperature. Wells were washed and a TMB substrate solution was added and kept at room temperature for 30 min. Finally, the stop solution was added, and the intensity of the color produced was measured at 450 nm. Concentration- inhibition response curve was established by GraphPad Prism 5.0. The IC_{50} value was calculated as the concentration at which 50% of the cells could survive in comparison to sorafenib.

code	IC50	conc	log	%inh	T2	T1	ΔT	RFU2	RFU1	ΔRFU	slope	K.Activity
**27RR**		100	2	93.59	30	0	30	6.41	0	6.41	3.3333	7.69208
		10	1	88.23	30	0	30	11.77	0	11.77	3.3333	14.1241
		1	0	70.32	30	0	30	29.68	0	29.68	3.3333	35.6164
		0.1	-1	51.08	30	0	30	48.92	0	48.92	3.3333	58.7046
		0.01	-2	35.75	30	0	30	64.25	0	64.25	3.3333	77.1008
EC				0	30	0	30	100	0	100	3.3333	120

code	IC50	conc	log	%inh	T2	T1	ΔT	RFU2	RFU1	ΔRFU	slope	K.Activity
Sorafenib		100	2	95.27	30	0	30	4.73	0	4.73	3.3333	5.67606
		10	1	90.93	30	0	30	9.07	0	9.07	3.3333	10.8841
		1	0	73.25	30	0	30	26.75	0	26.75	3.3333	32.1003
		0.1	-1	50.74	30	0	30	49.26	0	49.26	3.3333	59.1126
		0.01	-2	37.38	30	0	30	62.62	0	62.62	3.3333	75.1448
EC				0	30	0	30	100	0	100	3.3333	120

Mammalian cell lines culture

The anticancer profile of **T-1-AFPB** was checked on HepG2 and MCF-7 cell lines that were cultured on DMEM media. The cultured media were supplemented with 200 mM L-glutamine, 10.0% fetal bovine serum (Lonza), and 1.0% penicillin/streptomycin. Cells were seeded into 25.0 cm tissue culture flasks and incubated at 37°C in a 5.0% CO_2 incubator for 24 h or till confluency. The safety profile of **T-1-AFPB** was checked on one non-cancerous cell line (vero) to determine the treatments concentrations that do not depict toxic effects against the tested cells.

Determination of sample cytotoxicity on cells (MTT protocol)

1-the 96 well tissue culture plate was inoculated with 1 X 10^5 cells / ml (100 ul / well) and incubated at 37°C for 24 hours to develop a complete monolayer sheet.

2- Growth medium was decanted from 96 well micro titer plates after confluent sheet of cells were formed, cell monolayer was washed twice with wash media.

3- two-fold dilutions of tested sample was made in RPMI medium with 2% serum (maintenance medium).

4- 0.1 ml of each dilution was tested in different wells leaving 3 wells as control, receiving only maintenance medium.

5- Plate was incubated at 37°C and examined. Cells were checked for any physical signs of toxicity, e.g. partial or complete loss of the monolayer, rounding, shrinkage, or cell granulation.

6- MTT solution was prepared (5mg/ml in PBS) (BIO BASIC CANADA INC).

8- 20ul MTT solution were added to each well. Place on a shaking table, 150rpm for 5 minutes, to thoroughly mix the MTT into the media.

9) Incubate (37C, 5% CO2) for 4 hours to allow the MTT to be metabolized.

10) Dump off the media. (dry plate on paper towels to remove residue if necessary.

11) Resuspend formazan (MTT metabolic product) in 200ul DMSO. Place on a shaking table, 150rpm for 5 minutes, to thoroughly mix the formazan into the solvent.

12) Read optical density at 560nm and subtract background at 620nm. Optical density should be directly correlated with cell quantity.

T-1-AFPB Activity against Vero cells

Effect of sample 27RR on vero cells at different concentration

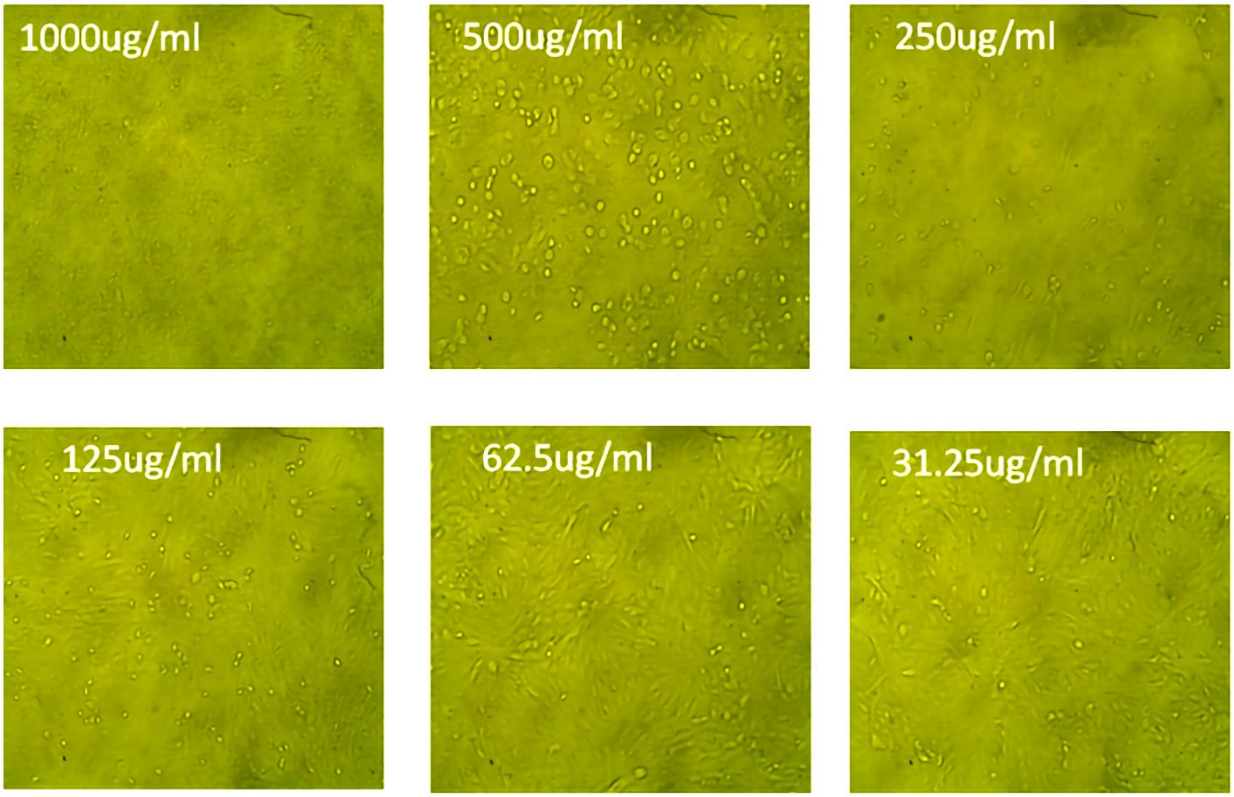

ID	ug/ml	O.D			Mean O.D	±SE	Viability %	Toxicity %	IC50 ± SD
Vero	--------	0.772	0.79	0.778	0.78	0.005292	100	0	ug
27RR (T-1-AFPB)	1000	0.06	0.053	0.055	0.056	0.002082	7.179487179	92.82051282	332.96 ± 9.43
	500	0.219	0.185	0.203	0.202333	0.009821	25.94017094	74.05982906	
	250	0.42	0.387	0.377	0.394667	0.012991	50.5982906	49.4017094	
	125	0.775	0.779	0.771	0.775	0.002309	99.35897436	0.641025641	
	62.5	0.77	0.785	0.778	0.777667	0.004333	99.7008547	0.299145299	
	31.25	0.774	0.791	0.771	0.778667	0.006227	99.82905983	0.170940171	

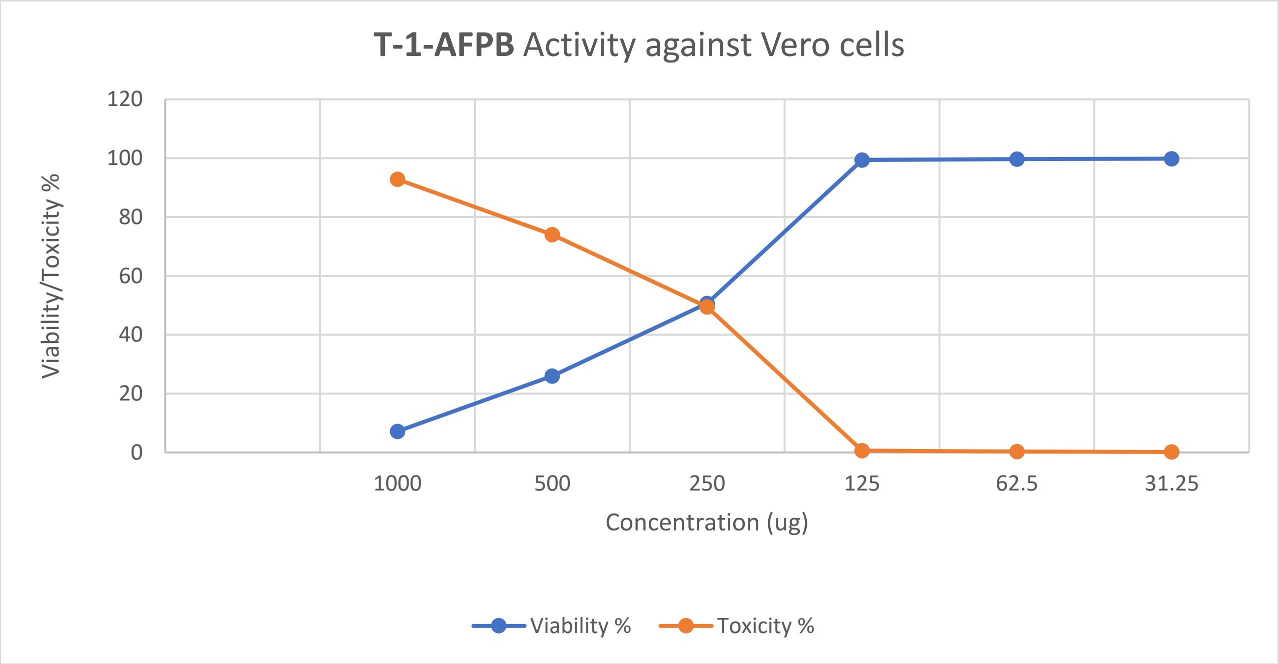

T-1-AFPB Activity against HepG2 cells

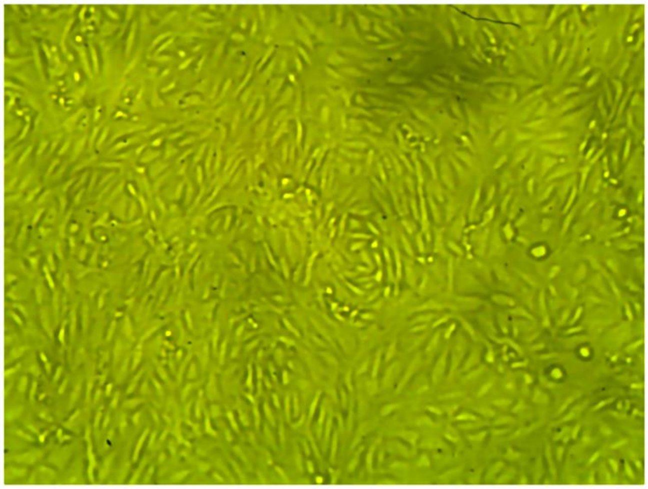

control HepG2 cells

Organism :	*Homo sapiens*, human
Tissue :	liver
Cell Type :	epithelial
Culture Properties :	adherent
Disease :	hepatocellular carcinoma
ATCC :	HB-8065

Effect of 27RR on HepG2 cells at different concentration

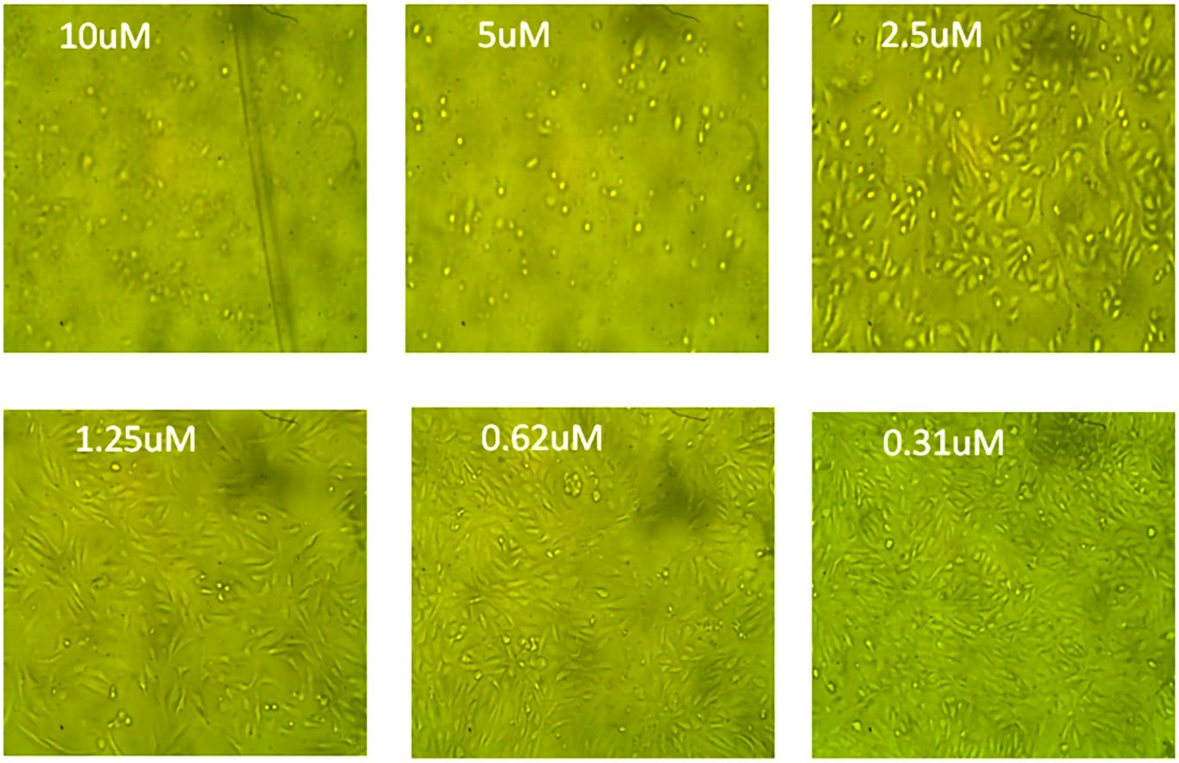

ID	uMl	O.D			Mean O.D	±SE	Viability %	Toxicity %	IC50 ± SD
HepG2	--------	0.759	0.772	0.764	0.765	0.003786	100	0	uM
27RR (**T-1-AFPB**)	10	0.027	0.033	0.035	0.031667	0.002404	4.139433551	95.86056645	2.24 ± 0.02
	5	0.054	0.044	0.048	0.048667	0.002906	6.361655773	93.63834423	
	2.5	0.317	0.327	0.308	0.317333	0.005487	41.48148148	58.51851852	
	1.25	0.64	0.617	0.622	0.626333	0.006984	81.87363834	18.12636166	
	0.625	0.76	0.768	0.759	0.762333	0.002848	99.65141612	0.348583878	
	0.312	0.756	0.774	0.761	0.763667	0.005364	99.82570806	0.174291939	

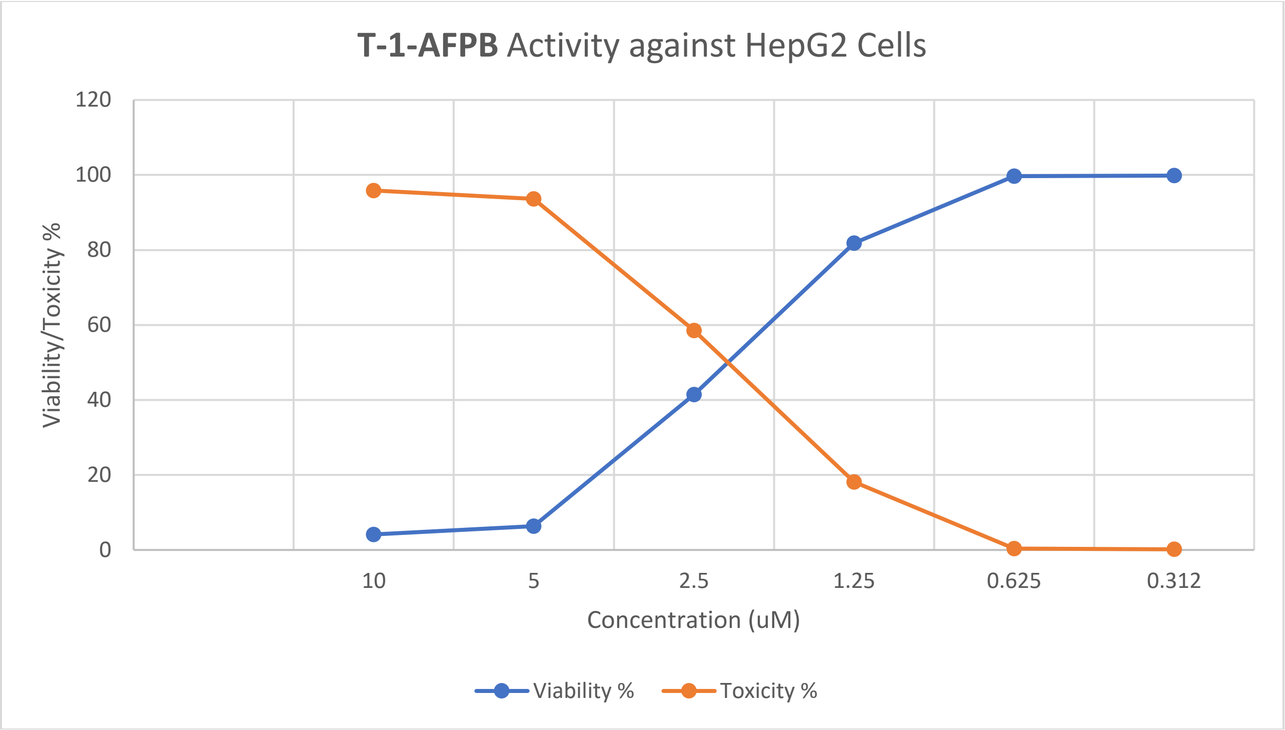

Sorafenib Activity against HepG2 cells

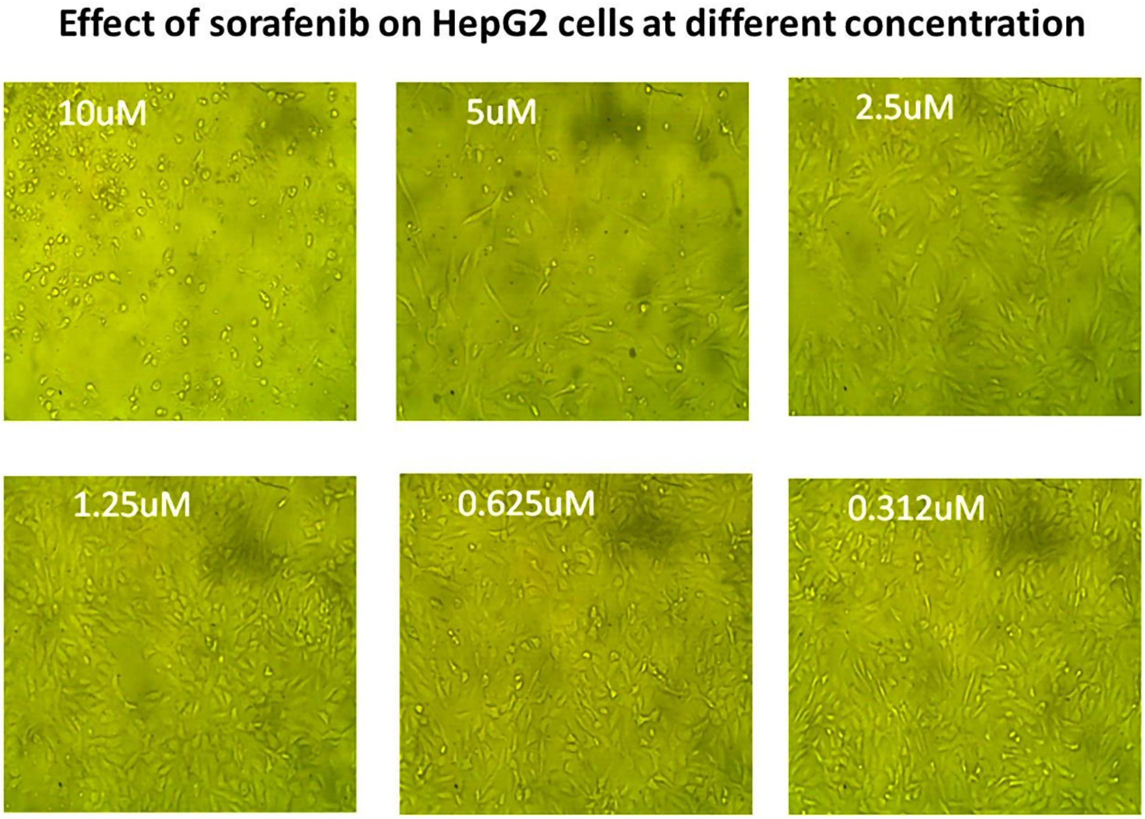

ID	uM/ml	O.D			Mean O.D	±SE	Viability %	Toxicity %	IC50 ± SD
HepG2	--------	0.759	0.763	0.77	0.764	0.003215	100	0	uM
sorafenib	10	0.023	0.021	0.026	0.023333	0.001453	3.054101222	96.94589878	2.24 ± 0.06
	5	0.127	0.162	0.158	0.149	0.01106	19.5026178	80.4973822	
	2.5	0.278	0.314	0.328	0.306667	0.014892	40.13961606	59.86038394	
	1.25	0.683	0.699	0.658	0.68	0.01193	89.0052356	10.9947644	
	0.625	0.747	0.77	0.756	0.757667	0.006692	99.17102967	0.828970332	
	0.312	0.764	0.769	0.757	0.763333	0.00348	99.91273997	0.087260035	

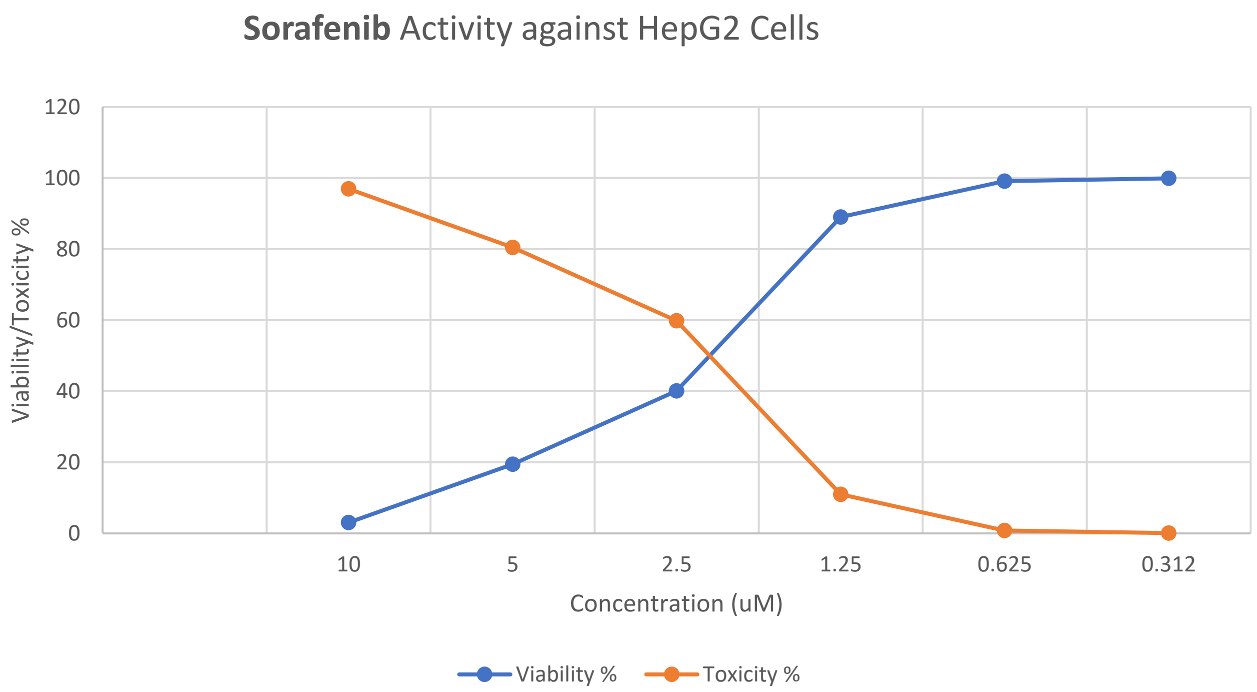

T-1-AFPB Activity against MCF-7 cells

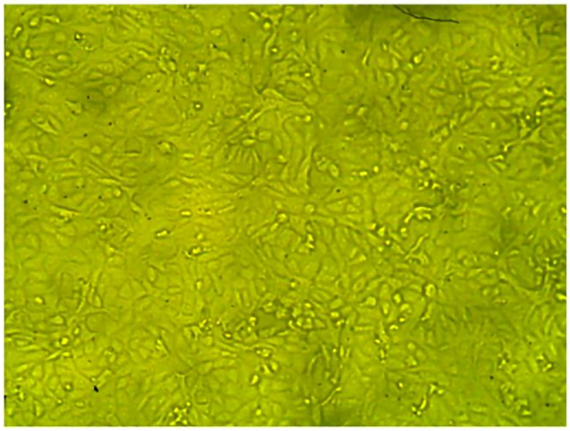

control
Mcf7 cells

Organism :	*Homo sapiens*, human
Tissue :	mammary gland, breast; derived from metastatic site: pleural effusion
Cell Type :	epithelial
Culture Properties :	adherent
Disease :	adenocarcinoma
ATCC :	HTB-22

Effect of 27RR on Mcf7 cells at different concentration

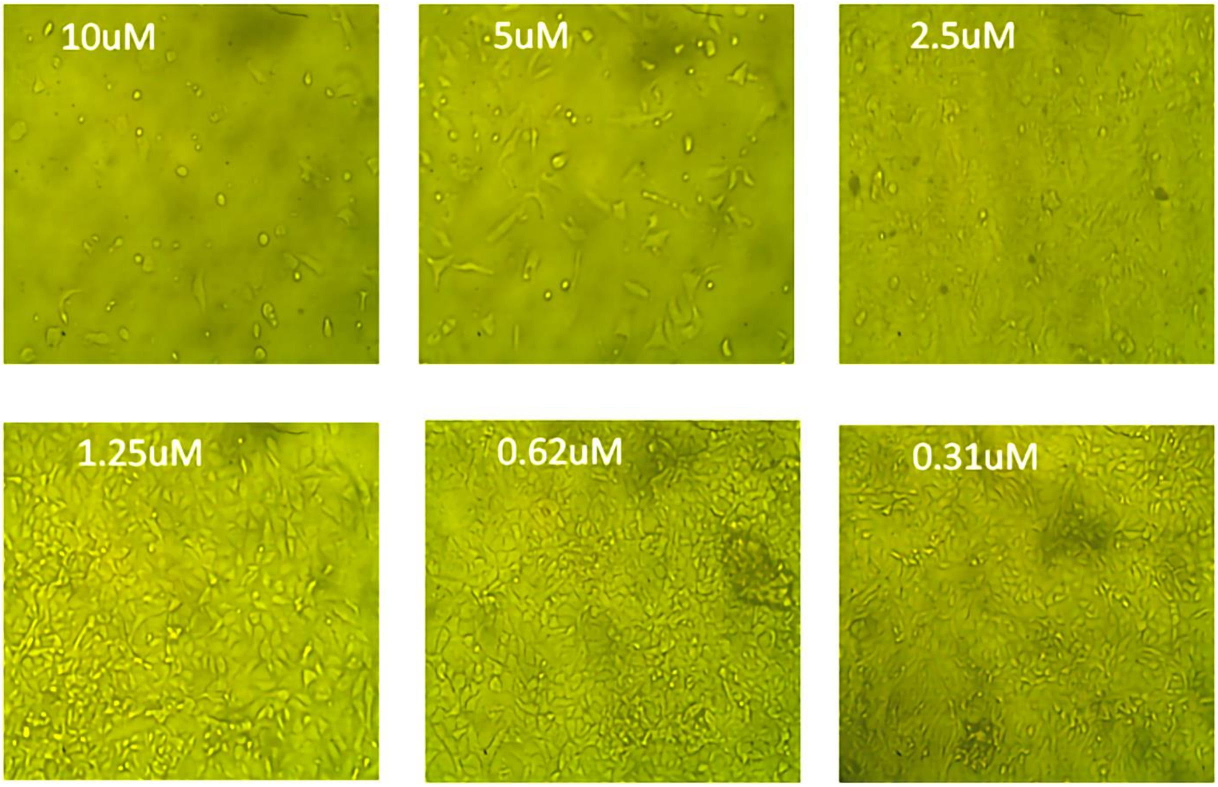

ID	uMl	O.D			Mean O.D	±SE	Viability %	Toxicity %	IC50 ± SD
Mcf7	--------	0.793	0.805	0.787	0.795	0.005292	100	0	uM
27RR (**T-1-AFPB**)	10	0.042	0.038	0.04	0.04	0.001155	5.031446541	94.96855346	3.26 ± 0.02
	5	0.173	0.188	0.179	0.18	0.004359	22.64150943	77.35849057	
	2.5	0.437	0.451	0.449	0.445667	0.004372	56.05870021	43.94129979	
	1.25	0.727	0.701	0.713	0.713667	0.007513	89.76939203	10.23060797	
	0.625	0.786	0.799	0.791	0.792	0.003786	99.62264151	0.377358491	
	0.312	0.786	0.803	0.792	0.793667	0.004978	99.83228512	0.167714885	

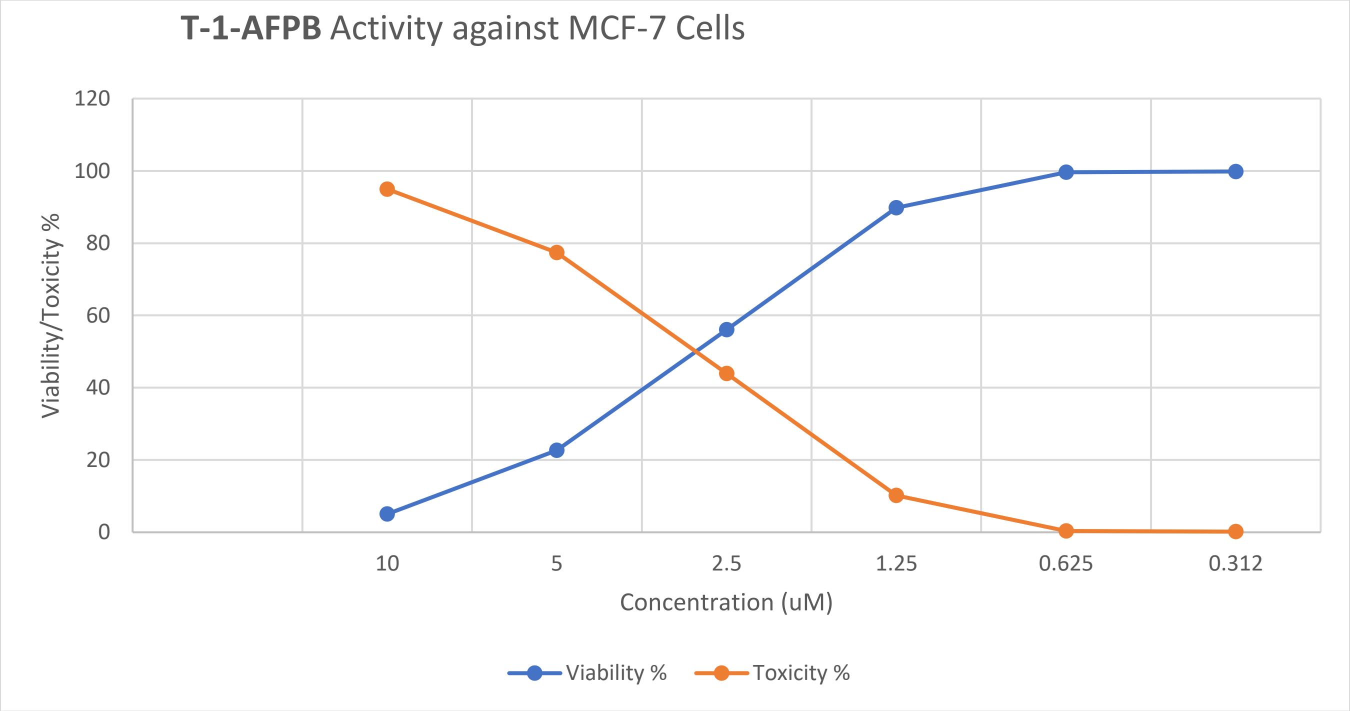

Sorafenib Activity against MCF-7 cells

Effect of sorafenib on Mcf7 cells at different concentration

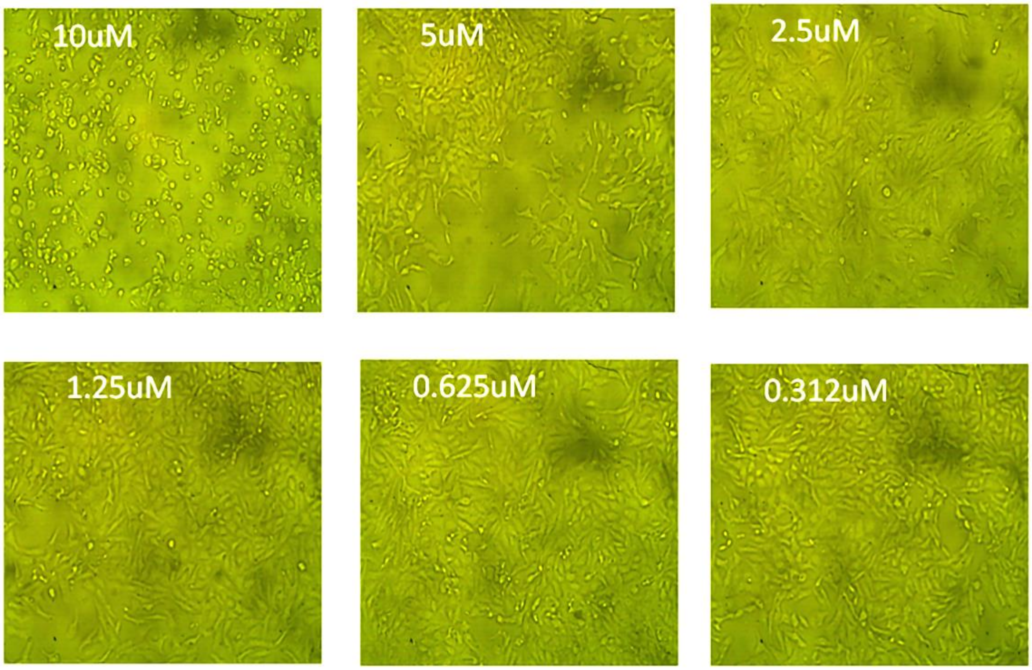

ID	uM/ml	O.D			Mean O.D	±SE	Viability %	Toxicity %	IC50 ± SD
Mcf7	--------	0.733	0.747	0.743	0.741	0.004163	100	0	uM
sorafenib	10	0.084	0.1	0.088	0.090667	0.004807	12.2357175	87.7642825	3.17 ± 0.01
	5	0.152	0.189	0.184	0.175	0.01159	23.61673414	76.38326586	
	2.5	0.387	0.367	0.372	0.375333	0.006009	50.6522717	49.3477283	
	1.25	0.657	0.673	0.661	0.663667	0.004807	89.56365272	10.43634728	
	0.625	0.735	0.742	0.741	0.739333	0.002186	99.77507872	0.224921278	
	0.312	0.74	0.746	0.736	0.740667	0.002906	99.95501574	0.044984256	

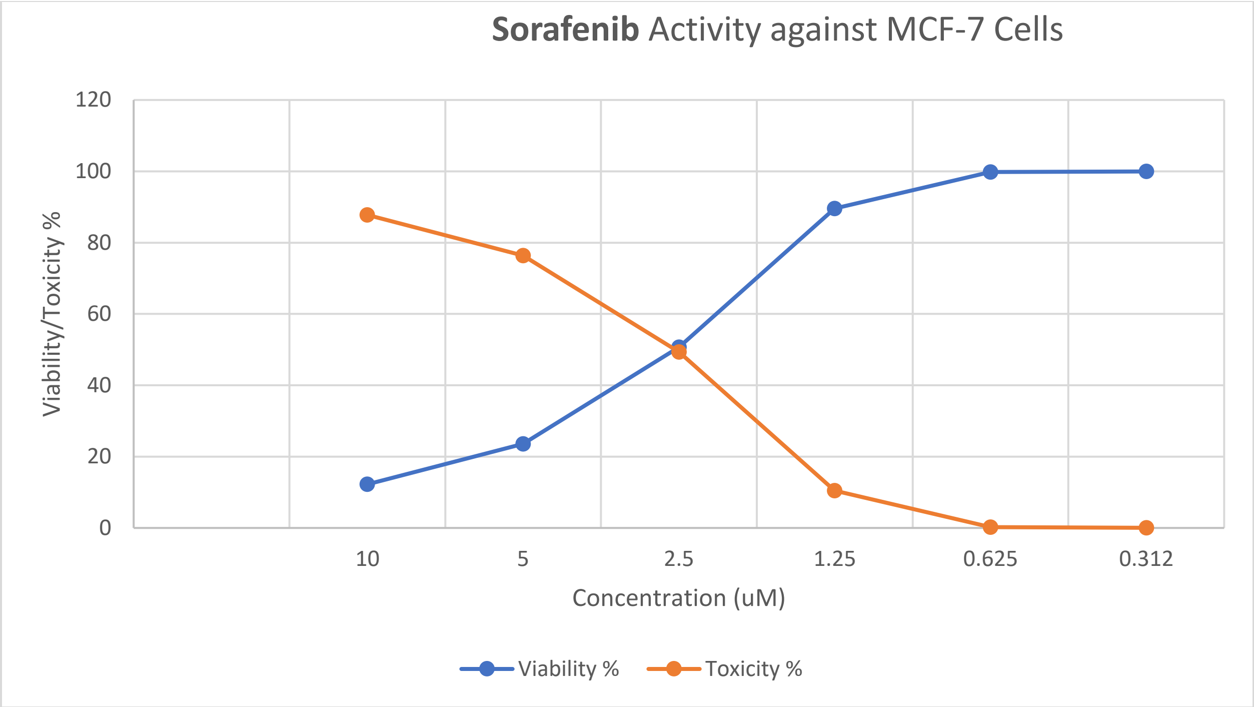

Selectivity index (SI)

The selectivity index values of the tested compounds on cancer cells were calculated as described by Koch et al., with slight modifications; SI=IC50nc/IC50cc, where IC50nc: the IC50 value of the tested compound on normal cells and IC50cc: IC50 of the tested compound on cancer cell line**.**

Flow cytometry analysis for apoptosis

Flow cytometry cell apoptosis analysis was used to investigate the apoptotic effect of the synthesized compound. HepG2 cells were treated with compound **T-1-APFPB** for 72h, collected by trypsin, centrifuged, washed two successive times with PBS, suspended in 500 µl binding buffer, and double stained with 5 µl Anne**xi**n V-FITC and 5 µl PI in the dark at room temperature for 15 min. The stained cells were measured using Epics XL-MCL™ Flow Cytometer and analyzed using software.

The utilized Reader: BD FACSCalibur

Kit used : ab139418_Propidium Iodide Flow Cytometry Kit/BD

Solvent : DMSO

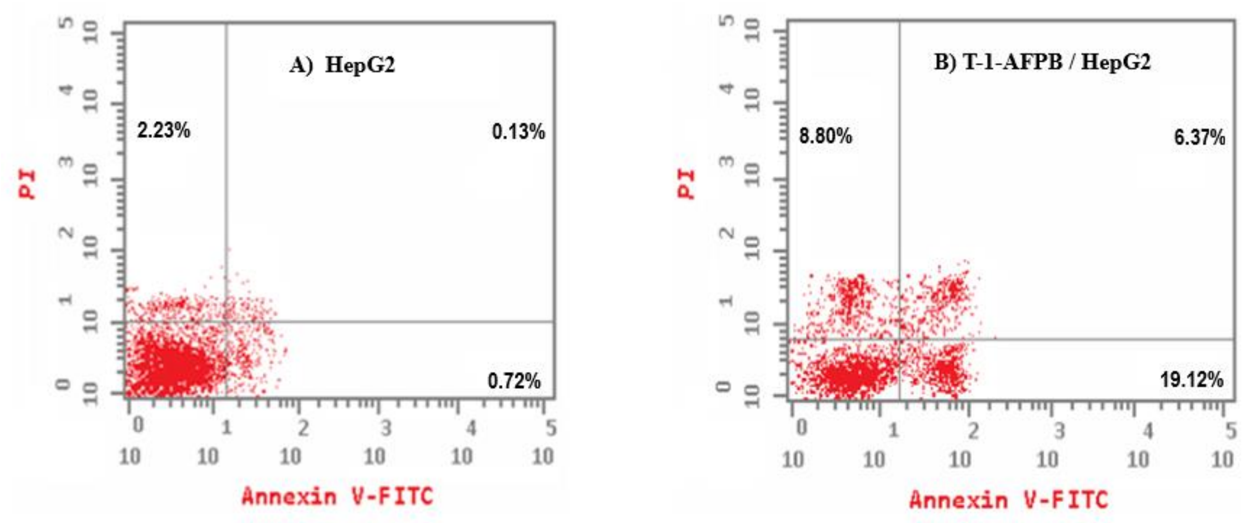

References:

1. Abraham MJ, Murtola T, Schulz R, Páll S, Smith JC, Hess B, et al. GROMACS: High performance molecular simulations through multi-level parallelism from laptops to supercomputers. SoftwareX. 2015;1:19–25.
2. Brooks BR, Brooks III CL, Mackerell Jr AD, Nilsson L, Petrella RJ, Roux B, et al. CHARMM: the biomolecular simulation program. J Comput Chem. 2009;30(10):1545–614.
3. Jo S, Cheng X, Islam SM, Huang L, Rui H, Zhu A, et al. Chapter Eight - CHARMM-GUI PDB Manipulator for Advanced Modeling and Simulations of Proteins Containing Nonstandard Residues. In: Karabencheva-Christova T, editor. Biomolecular Modelling and Simulations. Academic Press; 2014. p. 235–65. (Advances in Protein Chemistry and Structural Biology; vol. 96).
4. Jo S, Kim T, Iyer VG, Im W. CHARMM-GUI: A web-based graphical user interface for CHARMM. J Comput Chem. 2008;29(11):1859–65.
5. Lee J, Cheng X, Swails JM, Yeom MS, Eastman PK, Lemkul JA, et al. CHARMM-GUI input generator for NAMD, GROMACS, AMBER, OpenMM, and CHARMM/OpenMM simulations using the CHARMM36 additive force field. J Chem Theory Comput. 2016;12(1):405–13.
6. Bussi G, Donadio D, Parrinello M. Canonical sampling through velocity rescaling. J Chem Phys. 2007;126(1):14101.
7. Evans DJ, Holian BL. The nose–hoover thermostat. J Chem Phys. 1985;83(8):4069–74.
8. Hess B, Bekker H, Berendsen HJC, Fraaije JGEM. LINCS: a linear constraint solver for molecular simulations. J Comput Chem. 1997;18(12):1463–72.
9. Essmann U, Perera L, Berkowitz ML, Darden T, Lee H, Pedersen LG. A smooth particle mesh Ewald method. J Chem Phys. 1995;103(19):8577–93.
10. Humphrey W, Dalke A, Schulten K. VMD: visual molecular dynamics. J Mol Graph. 1996;14(1):33–8.
11. Tubiana T, Carvaillo JC, Boulard Y, Bressanelli S. TTClust: a versatile molecular simulation trajectory clustering program with graphical summaries. J Chem Inf Model. 2018;58(11):2178–82.
12. Salentin S, Schreiber S, Haupt VJ, Adasme MF, Schroeder M. PLIP: fully automated protein–ligand interaction profiler. Nucleic Acids Res. 2015 Jul 1;43(W1):W443–7.

13. Valdés-Tresanco MS, Valdés-Tresanco ME, Valiente PA, Moreno E. gmx_MMPBSA: A New Tool to Perform End-State Free Energy Calculations with GROMACS. J Chem Theory Comput. 2021;17(10):6281–91.
14. Miller III BR, McGee Jr TD, Swails JM, Homeyer N, Gohlke H, Roitberg AE. MMPBSA. py: an efficient program for end-state free energy calculations. J Chem Theory Comput. 2012;8(9):3314–21.
15. Xue W, Yang F, Wang P, Zheng G, Chen Y, Yao X, et al. What contributes to serotonin--norepinephrine reuptake inhibitors' dual-targeting mechanism? The key role of transmembrane domain 6 in human serotonin and norepinephrine transporters revealed by molecular dynamics simulation. ACS Chem Neurosci. 2018;9(5):1128–40.
16. Tuccinardi T. What is the current value of MM/PBSA and MM/GBSA methods in drug discovery? Vol. 16, Expert opinion on drug discovery. Taylor \& Francis; 2021. p. 1233–7.

Click or tap here to enter text.

Table S1. The QTAIM parameters (a.u.) at bond critical points (BCPs) of **T-1-APFPB**

BCP #	Atoms	(ρ)	(∇2ρ)	K(r)	G(r)	V(r)	H(r)
1	N1 - C2	0.352502	-1.06857	0.497118	0.229974	-0.72709	-0.49712
2	C2 - N3	0.318258	-0.73347	0.457905	0.274537	-0.73244	-0.45791
3	N1 - C5	0.335587	-1.00561	0.433965	0.182563	-0.61653	-0.43397
4	C5 - C6	0.32155	-0.90715	0.339163	0.112376	-0.45154	-0.33916
5	N4 - C5	0.307991	-0.87284	0.413154	0.194945	-0.6081	-0.41315
6	N3 - C6	0.294333	-0.69086	0.397483	0.224769	-0.62225	-0.39748
7	C6 - C7	0.2936	-0.79741	0.281677	0.082325	-0.364	-0.28168
8	C7 - N8	0.279722	-0.74691	0.328472	0.141744	-0.47022	-0.32847
9	N4 - C9	0.307609	-0.89567	0.391645	0.167728	-0.55937	-0.39165
10	N14 - H36	0.342557	-1.66365	0.470344	0.054431	-0.52478	-0.47034
11	N8 - C9	0.287688	-0.78716	0.334362	0.137571	-0.47193	-0.33436
12	C7 - O10	0.406059	-0.33209	0.682067	0.599046	-1.28111	-0.68207
13	C9 - O11	0.41344	-0.35262	0.701718	0.613564	-1.31528	-0.70172
14	N8 - C12	0.249249	-0.60247	0.289303	0.138685	-0.42799	-0.2893
15	C12 - C13	0.24663	-0.5707	0.197446	0.054771	-0.25222	-0.19745
16	N8 - H36	0.016282	0.067149	-0.00267	0.014118	-0.01145	0.00267
17	N14 - C15	0.284469	-0.76833	0.368401	0.176318	-0.54472	-0.3684
18	C12 - H34	0.284633	-0.98694	0.282392	0.035657	-0.31805	-0.28239
19	C13 - N14	0.31066	-0.86225	0.423002	0.207439	-0.63044	-0.423
20	C13 - O16	0.409063	-0.25462	0.689543	0.625887	-1.31543	-0.68954
21	O16 - H46	0.017506	0.064124	-0.00217	0.013857	-0.01168	0.002173
22	N4 - C17	0.245557	-0.57337	0.292624	0.149283	-0.44191	-0.29262
23	N3 - C18	0.249585	-0.59598	0.302016	0.15302	-0.45504	-0.30202
24	C15 - C19	0.305081	-0.842	0.307787	0.097285	-0.40507	-0.30779
25	C19 - C20	0.313267	-0.88167	0.325107	0.10469	-0.4298	-0.32511
26	C20 - H44	0.284954	-0.99431	0.283974	0.035396	-0.31937	-0.28397
27	C21 - C22	0.305027	-0.83414	0.307754	0.099219	-0.40697	-0.30775
28	C20 - C21	0.30575	-0.84543	0.308815	0.097458	-0.40627	-0.30882
29	C15 - C23	0.305212	-0.84532	0.310936	0.099605	-0.41054	-0.31094
30	C23 - H46	0.28836	-1.0171	0.289612	0.035337	-0.32495	-0.28961
31	C22 - C23	0.309085	-0.85935	0.316382	0.101544	-0.41793	-0.31638
32	C24 - N25	0.306262	-0.8566	0.411405	0.197257	-0.60866	-0.41141
33	C21 - C24	0.260265	-0.63678	0.219994	0.0608	-0.28079	-0.21999
34	C24 - O26	0.404153	-0.29762	0.677597	0.603191	-1.28079	-0.6776
35	O26 - H52	0.017555	0.064326	-0.00215	0.013931	-0.01178	0.002151
36	C31 - C32	0.308172	-0.8564	0.314326	0.100225	-0.41455	-0.31433
37	N25 - C27	0.281812	-0.75628	0.36232	0.173249	-0.53557	-0.36232
38	C27 - C32	0.304445	-0.83975	0.309625	0.099688	-0.40931	-0.30963
39	C30 - C31	0.309524	-0.86613	0.316922	0.100389	-0.41731	-0.31692
40	C31 - H51	0.28186	-0.97105	0.280925	0.038164	-0.31909	-0.28093
41	C27 - C28	0.305077	-0.83878	0.307587	0.097892	-0.40548	-0.30759
42	C29 - C30	0.308287	-0.85996	0.314688	0.099699	-0.41439	-0.31469

BCP #	Atoms	(ρ)	(∇2ρ)	K(r)	G(r)	V(r)	H(r)
43	N25 - H47	0.343733	-1.65232	0.46856	0.05548	-0.52404	-0.46856
44	C28 - C29	0.310199	-0.86528	0.318838	0.102517	-0.42136	-0.31884
45	C30 - H50	0.281235	-0.96491	0.280346	0.039119	-0.31947	-0.28035
46	C32 - H52	0.288207	-1.01504	0.289444	0.035686	-0.32513	-0.28944
47	C2 - H33	0.288383	-1.02565	0.288499	0.032088	-0.32059	-0.2885
48	C12 - H35	0.283911	-0.98203	0.281339	0.035833	-0.31717	-0.28134
49	C19 - H43	0.278959	-0.94765	0.277161	0.04025	-0.31741	-0.27716
50	C17 - H37	0.280464	-0.95658	0.276664	0.03752	-0.31418	-0.27666
51	C17 - H38	0.281795	-0.96642	0.278712	0.037108	-0.31582	-0.27871
52	C17 - H39	0.284489	-0.98637	0.28296	0.036367	-0.31933	-0.28296
53	C18 - H40	0.280775	-0.96055	0.278317	0.038179	-0.3165	-0.27832
54	C18 - H41	0.282	-0.96878	0.278759	0.036566	-0.31533	-0.27876
55	C18 - H42	0.282274	-0.97094	0.279144	0.036409	-0.31555	-0.27914
56	C22 - H47	0.012453	0.054665	-0.0027	0.010966	-0.00827	0.0027
57	C22 - H45	0.281202	-0.96358	0.280544	0.039648	-0.32019	-0.28054
58	C28 - H48	0.278282	-0.94228	0.276518	0.040948	-0.31747	-0.27652
59	C29 - H49	0.281556	-0.96875	0.280667	0.038479	-0.31915	-0.28067

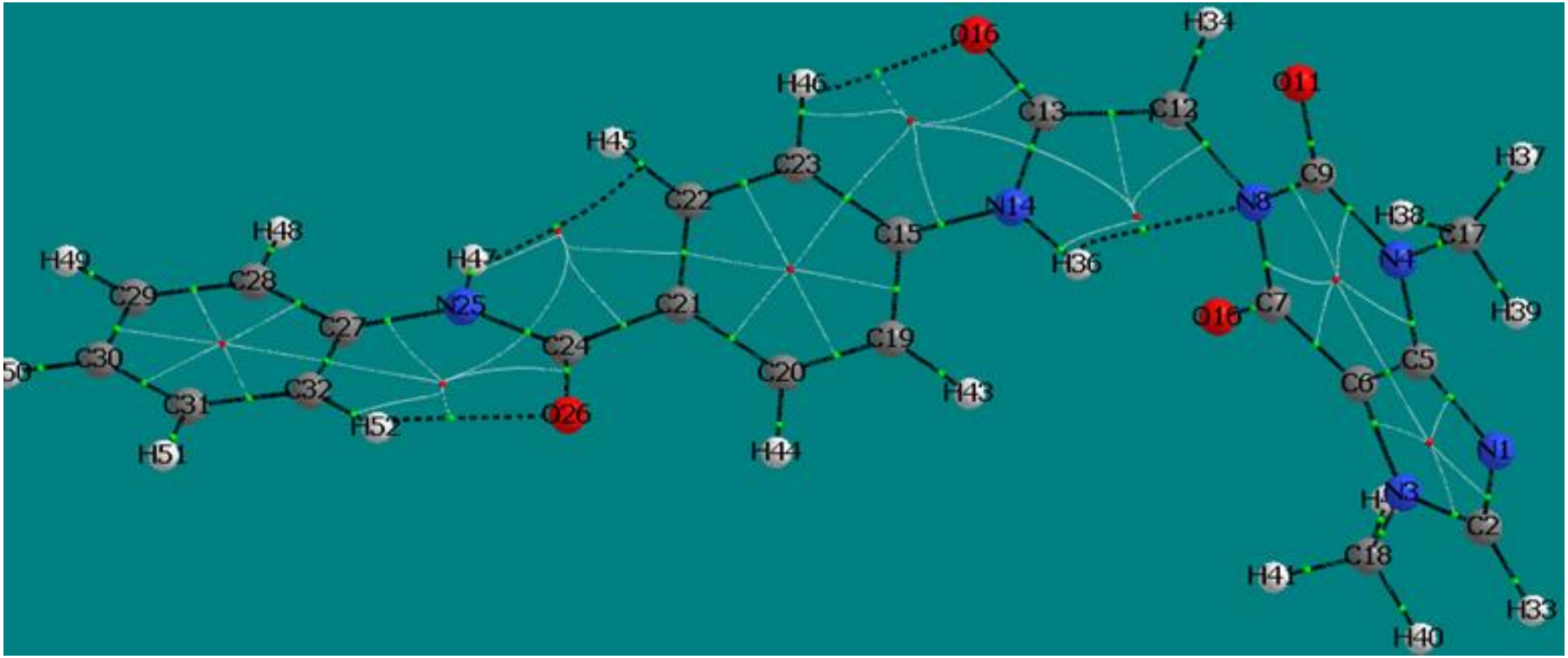

Figure S1. QTAIM analysis: molecular graph showing the bonding critical points (BCP) for **T-1-APFPB**.

Spectra of theobromine

Ibraheim Eissa-T-2-proton-AS-insol

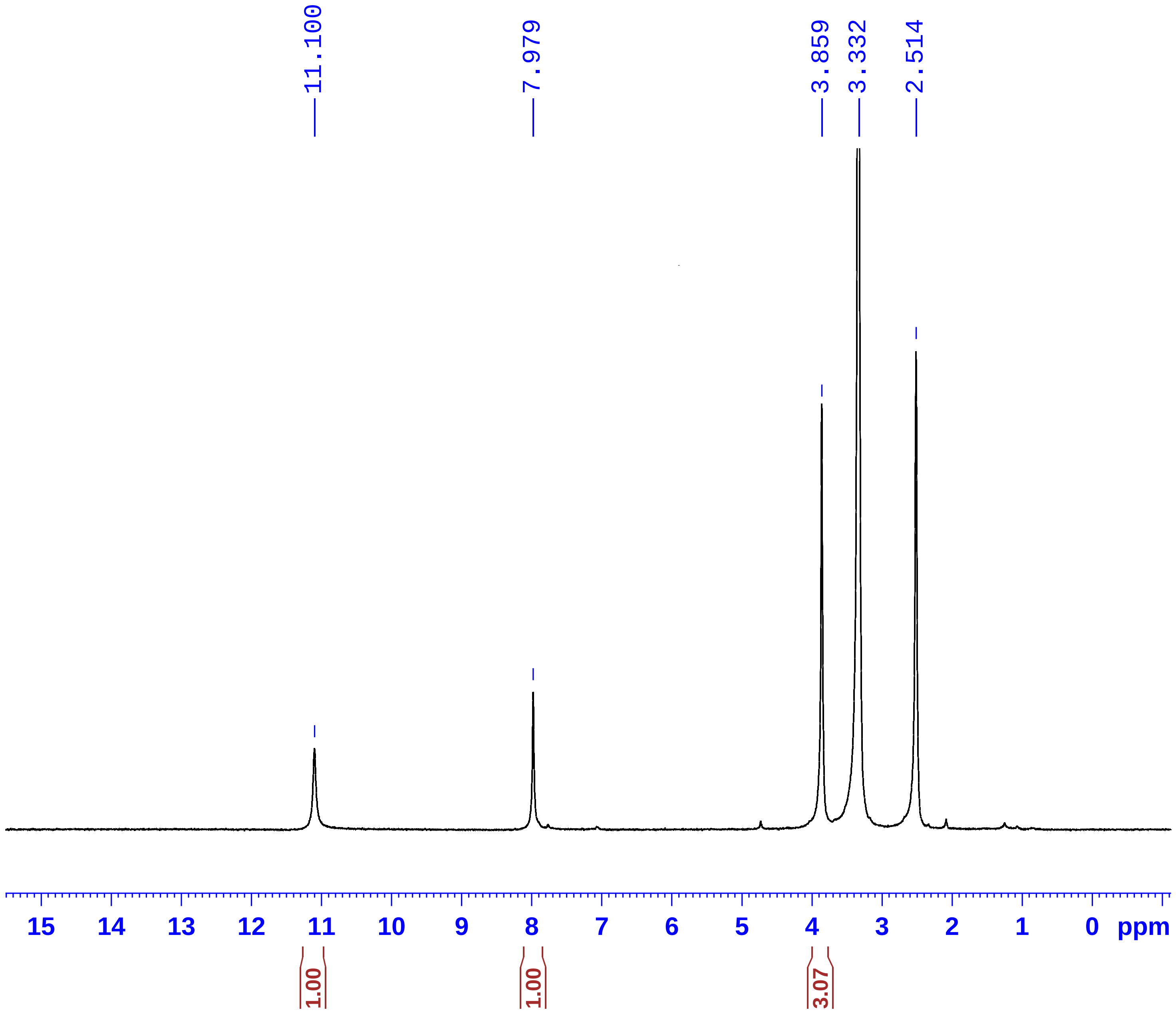

Faculty of Pharmacy

Current Data Parameters

NAME	Ibraheim Eissa-T-2-proton-AS-insol
EXPNO	10
PROCNO	1

F2 - Acquisition Parameters

Date_	20230409
Time	10.33 h
INSTRUM	spect
PROBHD	Z108618_0945 (
PULPROG	zg30
TD	65536
SOLVENT	DMSO
NS	16
DS	2
SWH	8012.820 Hz
FIDRES	0.244532 Hz
AQ	4.0894465 sec
RG	135.42
DW	62.400 usec
DE	6.50 usec
TE	299.9 K
D1	1.00000000 sec
TD0	1
SFO1	400.2024712 MHz
NUC1	1H
P1	13.50 usec
PLW1	13.00000000 W

F2 - Processing parameters

SI	65536
SF	400.2000000 MHz
WDW	EM
SSB	0
LB	0.30 Hz
GB	0
PC	1.00

Ibraheim Eissa-T-2-proton-AS-insol

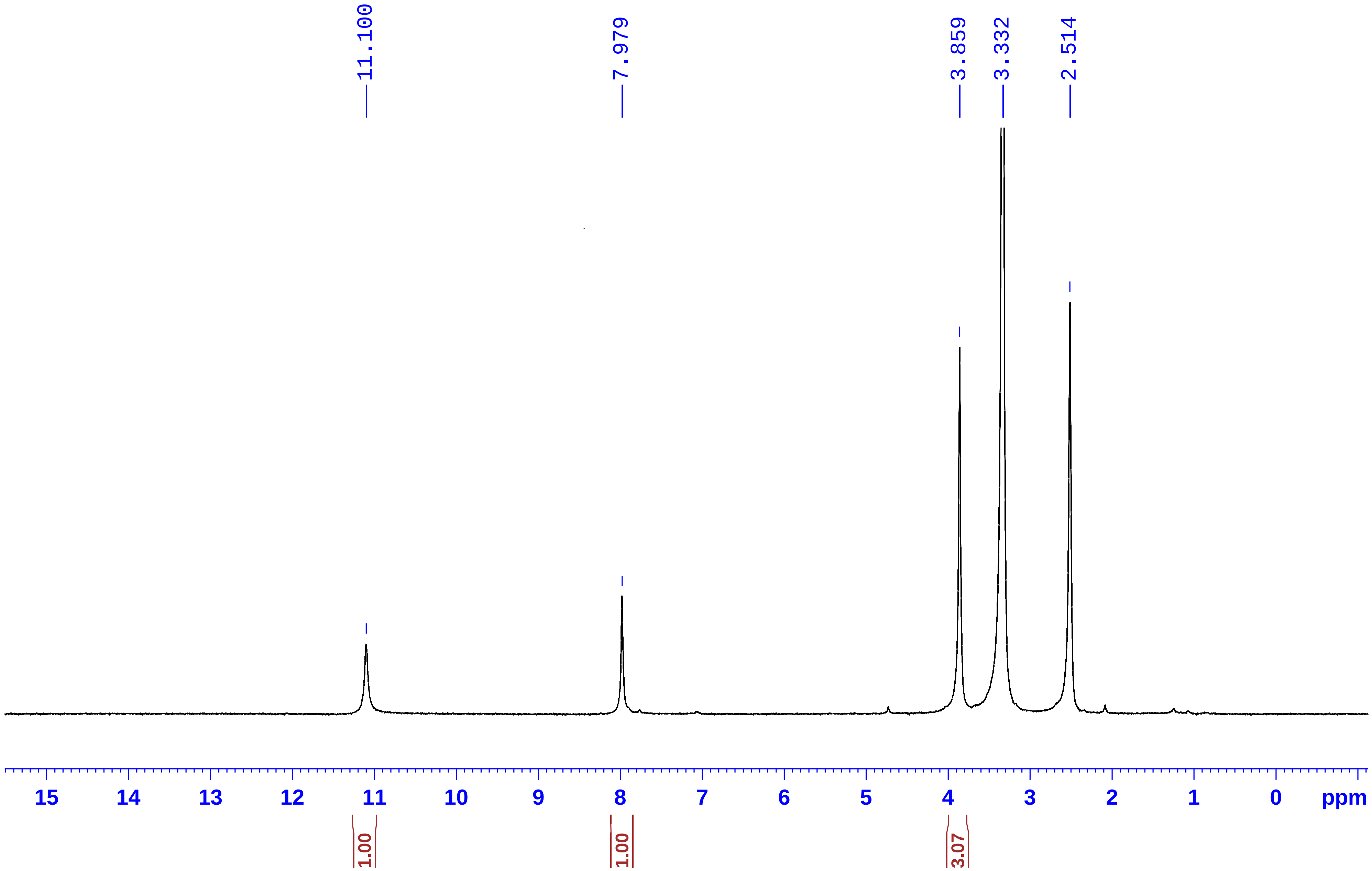

Ibraheim Eissa-T-2-proton-AS-insol

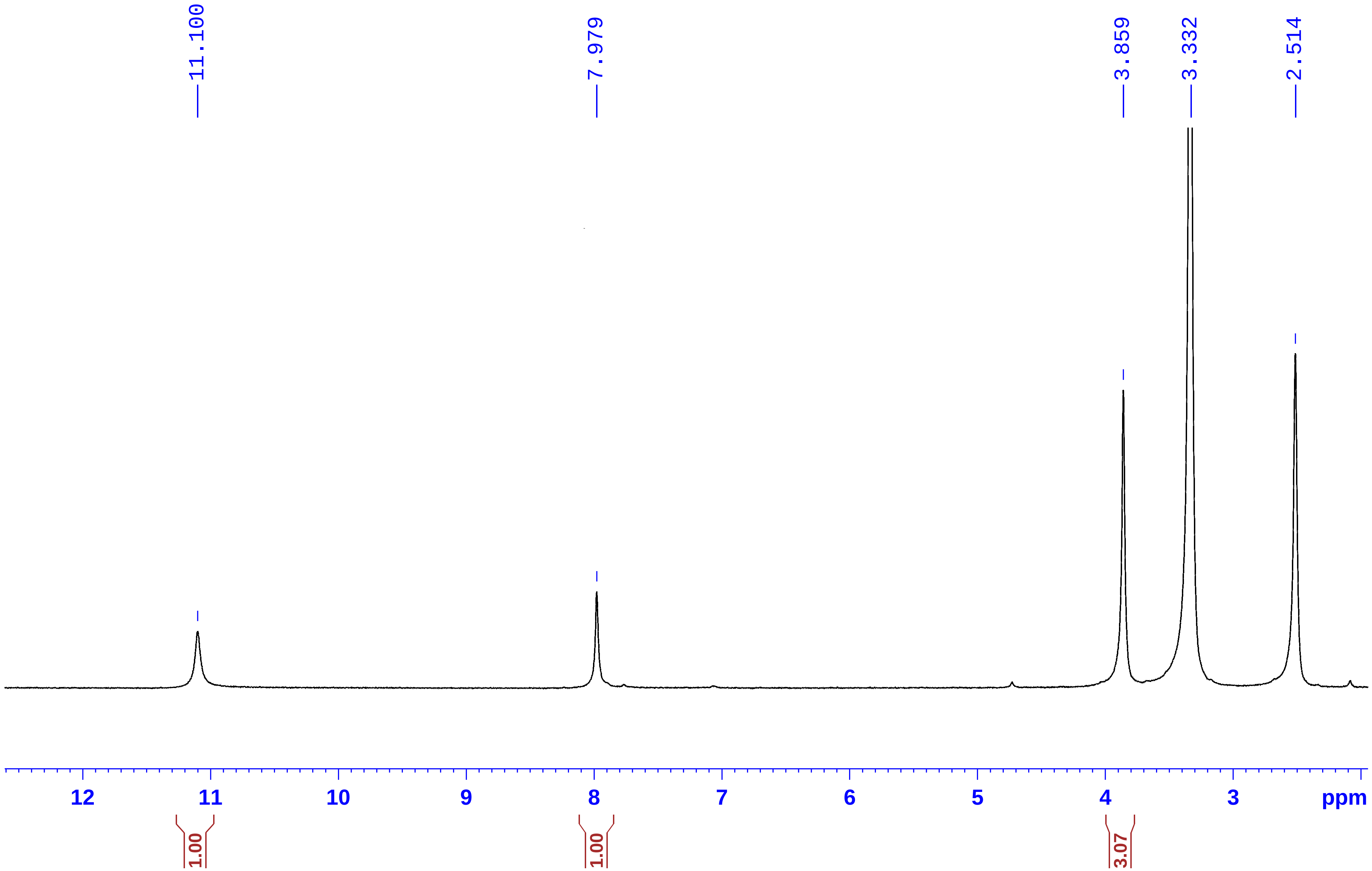

Ibraheim Eissa-T2-carbon-DMSO-AS

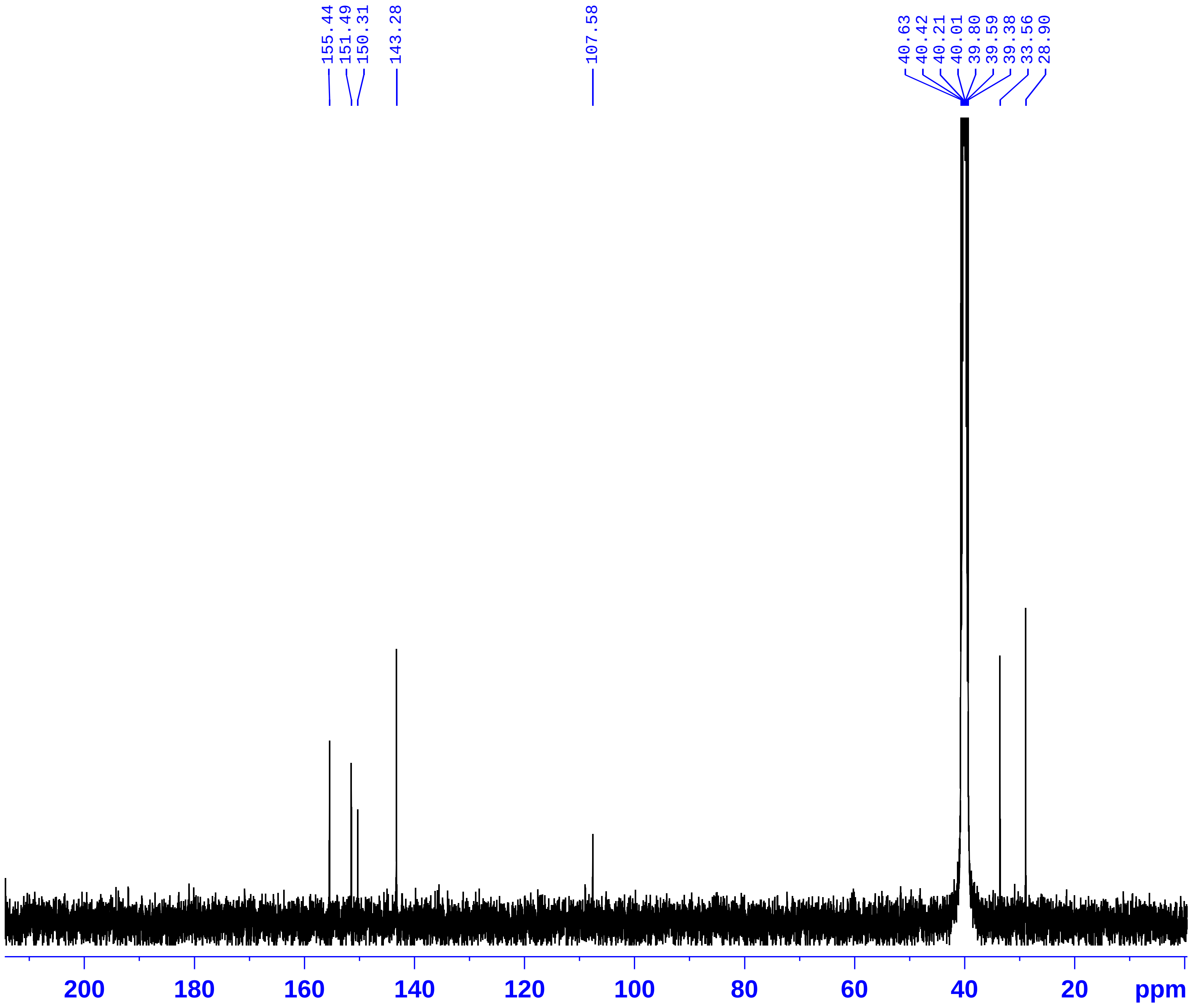

BRUKER

Faculty of Pharmacy

Current Data Parameters

NAME	Ibraheim Eissa-T2-carbon-DMSO-AS
EXPNO	10
PROCNO	1

F2 - Acquisition Parameters

Date_	20230409
Time	15.25 h
INSTRUM	spect
PROBHD	Z108618_0945 (
PULPROG	zgpg30
TD	65536
SOLVENT	DMSO
NS	2100
DS	4
SWH	24038.461 Hz
FIDRES	0.733596 Hz
AQ	1.3631488 sec
RG	197.77
DW	20.800 usec
DE	6.50 usec
TE	298.7 K
D1	2.00000000 sec
D11	0.03000000 sec
TD0	1
SFO1	100.6404331 MHz
NUC1	13C
P1	10.00 usec
PLW1	47.00000000 W
SFO2	400.2016008 MHz
NUC2	1H
CPDPRG[2	waltz16
PCPD2	90.00 usec
PLW2	13.00000000 W
PLW12	0.29249999 W
PLW13	0.14713000 W

F2 - Processing parameters

SI		32768
SF		100.6303700 MHz
WDW		EM
SSB	0	
LB		1.00 Hz
GB	0	
PC		1.40

Ibraheim Eissa-T2-carbon-DMSO-AS

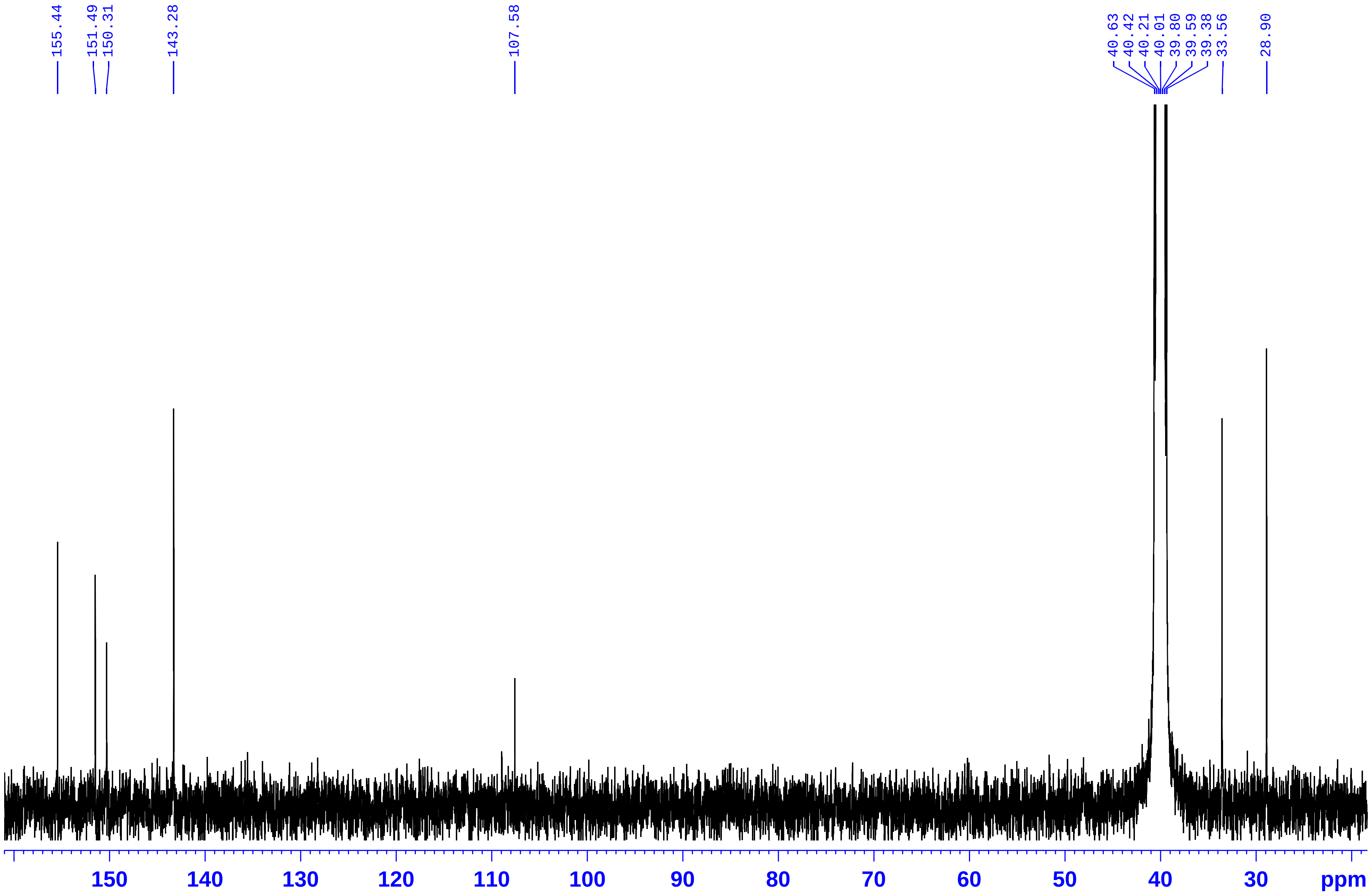

Ibraheim Eissa-T2-carbon-DMSO-AS

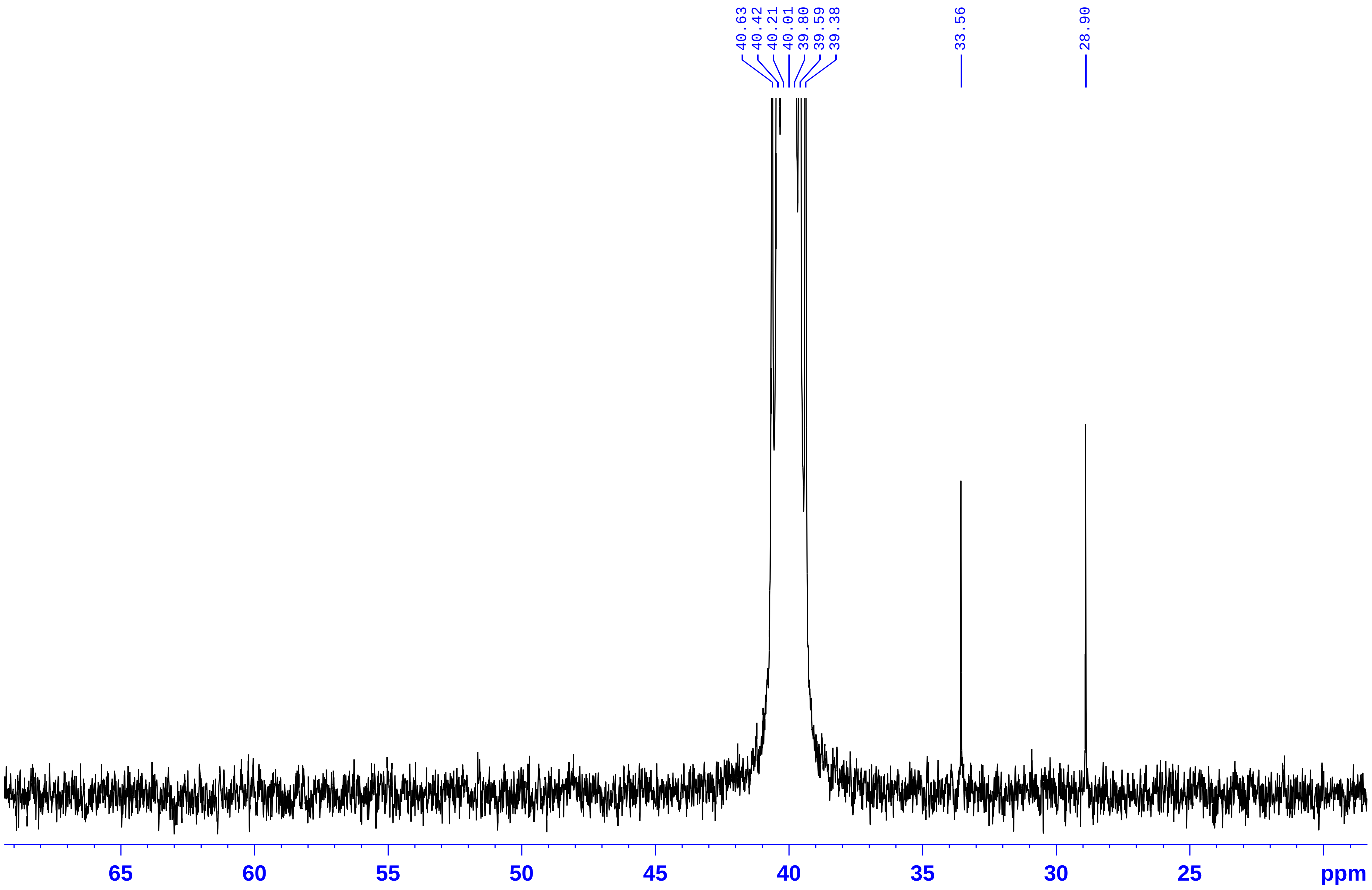

Ibraheim Eissa-T2-carbon-DMSO-AS

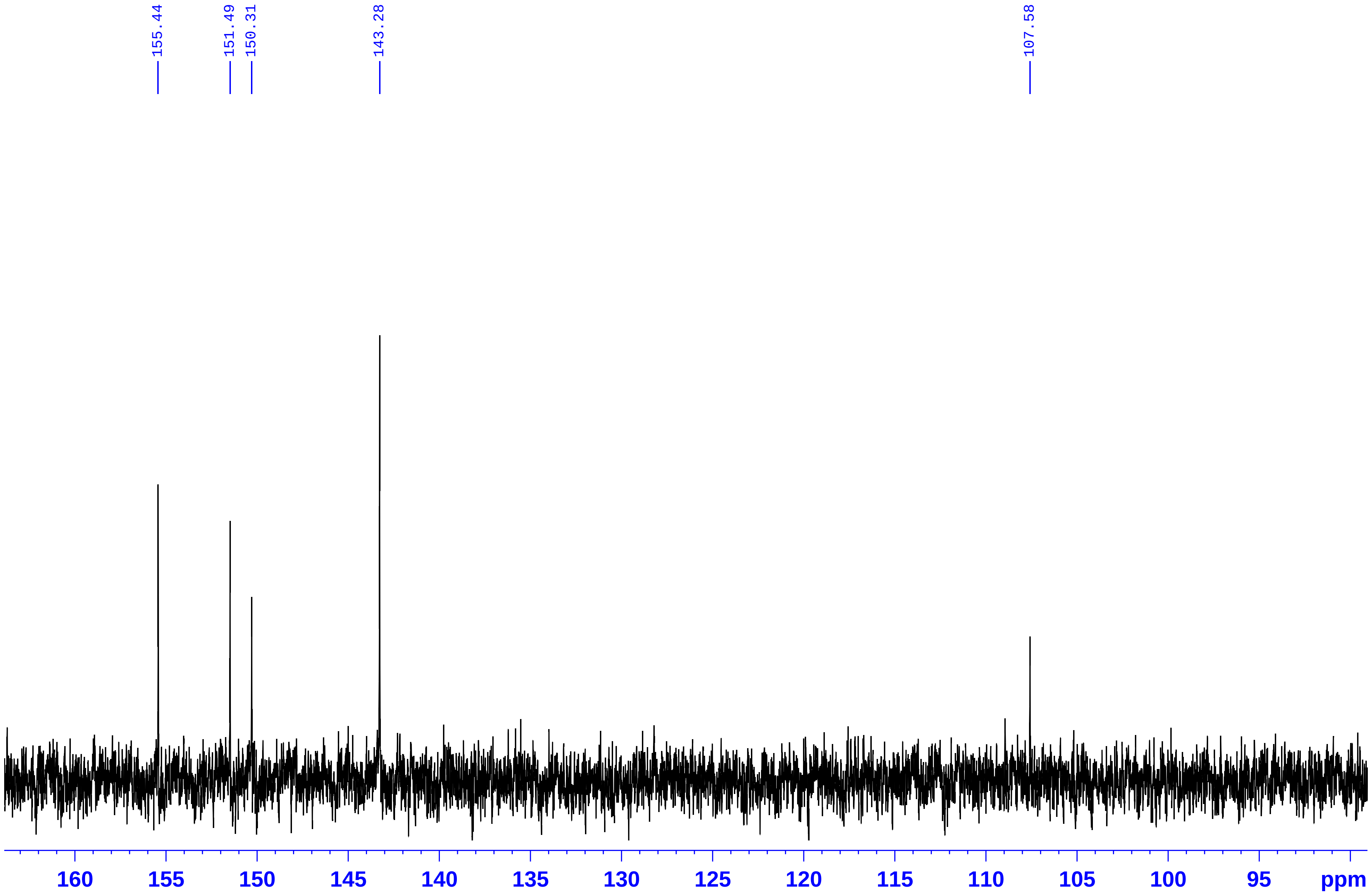

Spectra of intermediate (4)

IR of compound 4

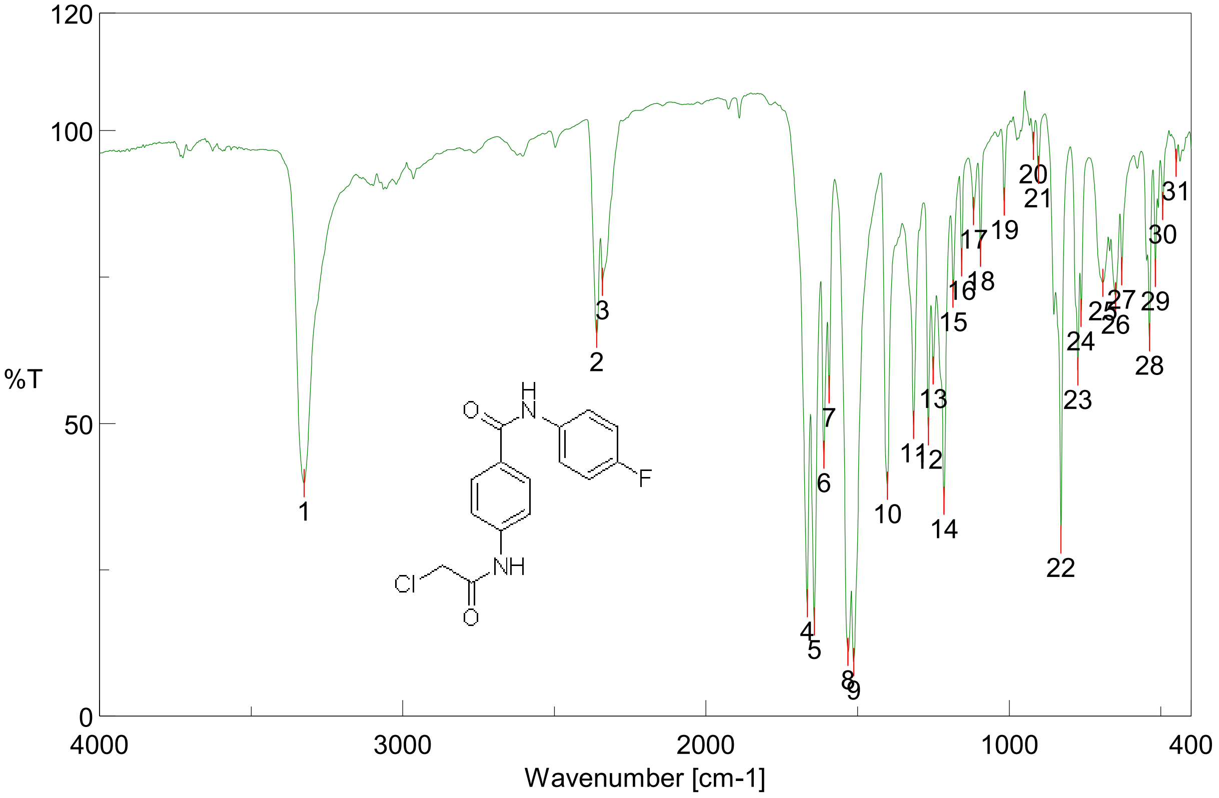

[Comments]

Sample name	A27
Comment	
User	
Division	
Company	KSU

[Detailed Information]

Creation date	9/15/2020 4:58 AM
Data array type	Linear data array
Horizontal axis	Wavenumber [cm-1]
Vertical axis	%T
Start	399.193 cm-1
End	4000.6 cm-1
Data interval	0.964233 cm-1
Data points	3736

[Measurement Information]

Model Name	FT/IR-6600typeA
Serial Number	A014661790
Measurement Date	9/15/2020 4:57 AM
Light Source	Standard
Detector	TGS
Accumulation	Auto (15)
Resolution	4 cm-1
Zero Filling	On
Apodization	Cosine
Gain	Auto (1)
Aperture	Auto (7.1 mm)
Scanning Speed	Auto (2 mm/sec)
Filter	Auto (10000 Hz)

[Result of Peak Picking]

No.	Position	Intensity	No.	Position	Intensity
1	3325.64	39.7597	2	2360.44	65.2491

[Result of Peak Picking]

No.	Position	Intensity	No.	Position	Intensity
3	2341.16	74.1223	4	1666.2	19.2991
5	1643.05	16.1865	6	1611.23	44.6329
7	1593.88	55.7822	8	1531.2	10.959
9	1512.88	9.25398	10	1402	39.3057
11	1315.21	49.7137	12	1267	48.6261
13	1250.61	58.9645	14	1214.93	36.7777
15	1185.04	72.0892	16	1157.08	77.4682
17	1117.55	86.1791	18	1094.4	79.0945
19	1016.3	87.8543	20	920.843	97.3507
21	903.487	93.2202	22	829.241	30.1686
23	773.315	58.8499	24	762.709	68.846
25	691.355	73.9718	26	648.929	71.6788
27	628.68	75.9033	28	537.078	64.6761
29	517.793	75.6658	30	493.688	87.0821
31	449.333	94.4481			

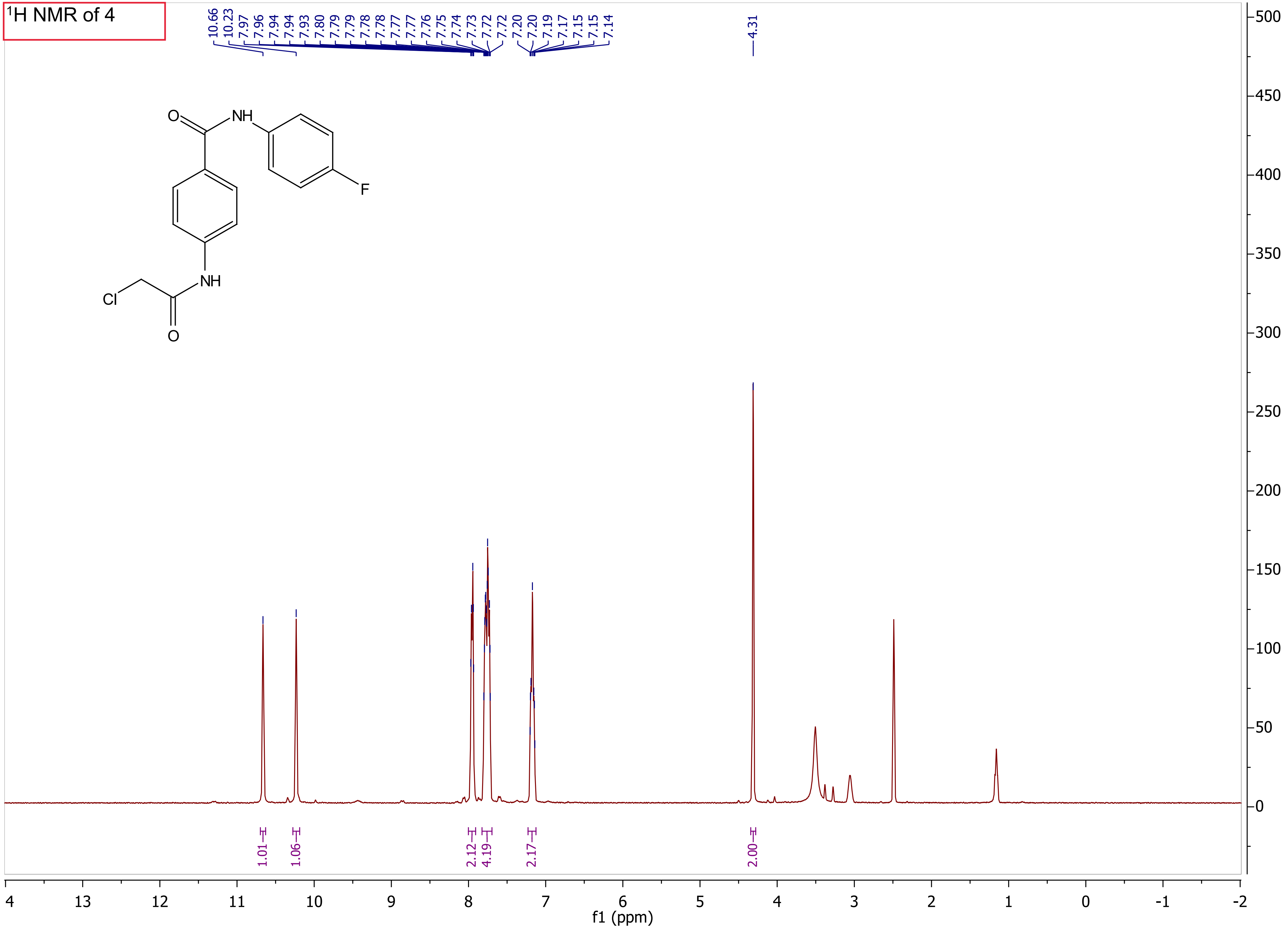

1H NMR of 4
10.66
10.23
7.97
7.96
7.94
7.94
7.93
7.80
7.79
7.79
7.78
7.78
7.77
7.77
7.76
7.75
7.74
7.73
7.72
7.72
7.20
7.20
7.19
7.17
7.15
7.15
7.14
4.31
O
NH
F
NH
Cl
O
1.01
1.06
2.12
4.19
2.17
2.00
f1 (ppm)

^{1}H NMR of 4

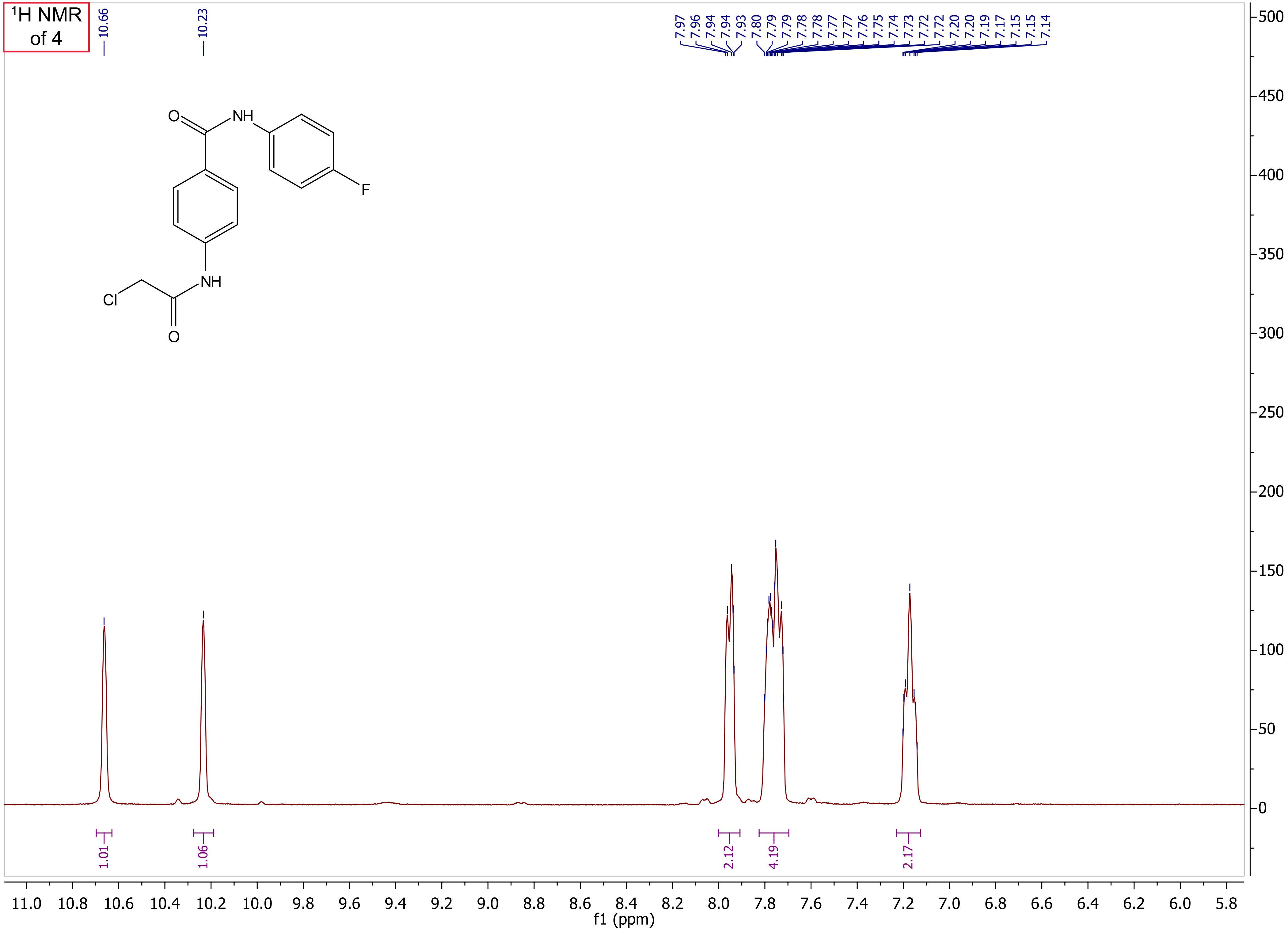

10.66
10.23
7.97
7.96
7.94
7.94
7.93
7.80
7.79
7.79
7.78
7.78
7.77
7.77
7.76
7.75
7.74
7.73
7.72
7.72
7.20
7.20
7.19
7.17
7.15
7.15
7.14
O
NH
F
NH
Cl
O
1.01
1.06
2.12
4.19
2.17
f1 (ppm)

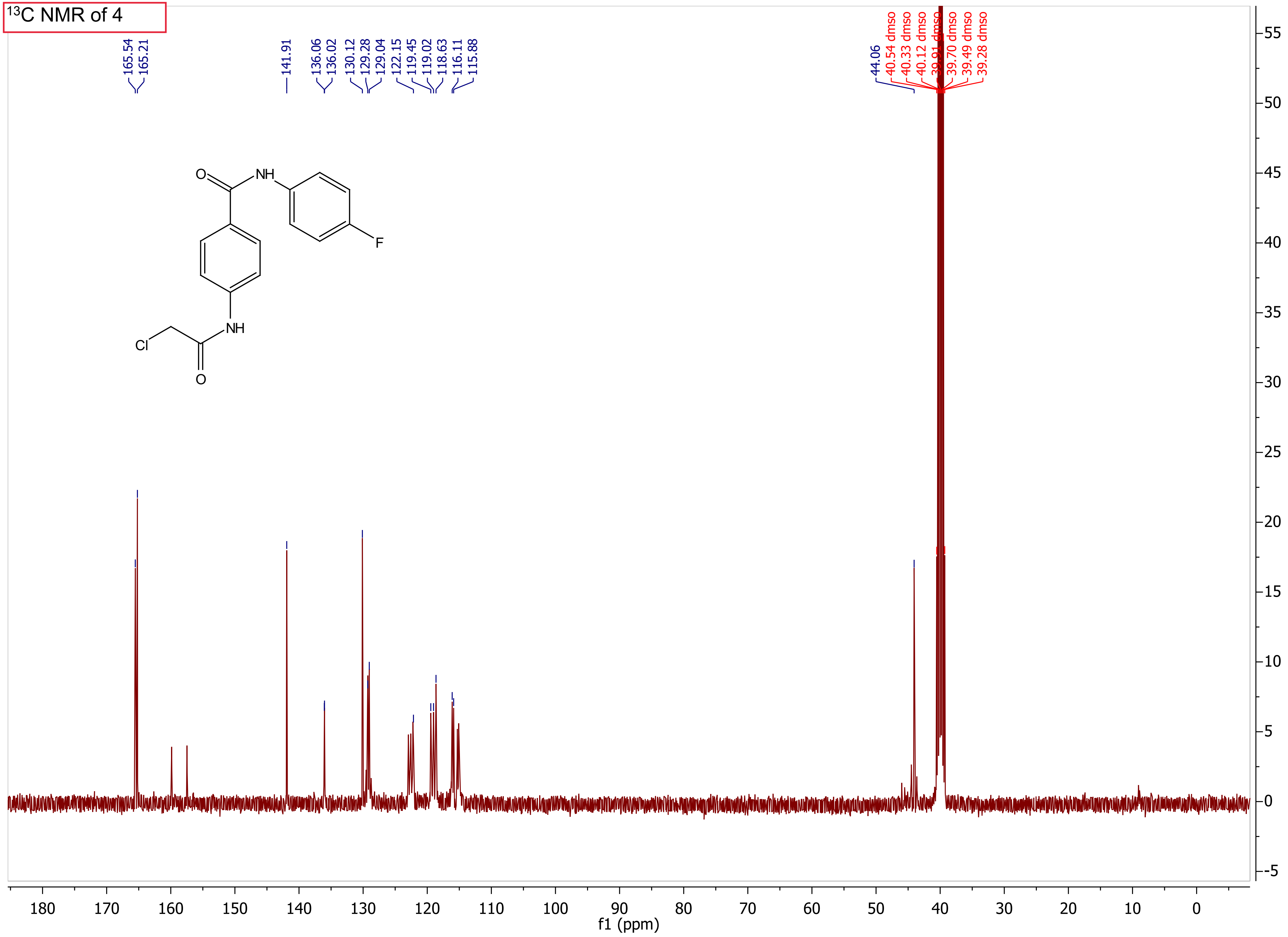

13C NMR of 4
165.54
165.21
141.91
136.06
136.02
130.12
129.28
129.04
122.15
119.45
119.02
118.63
116.11
115.88
44.06
40.54 dmso
40.33 dmso
40.12 dmso
39.91 dmso
39.70 dmso
39.49 dmso
39.28 dmso
O
NH
F
Cl
NH
O
f1 (ppm)

Spectral data

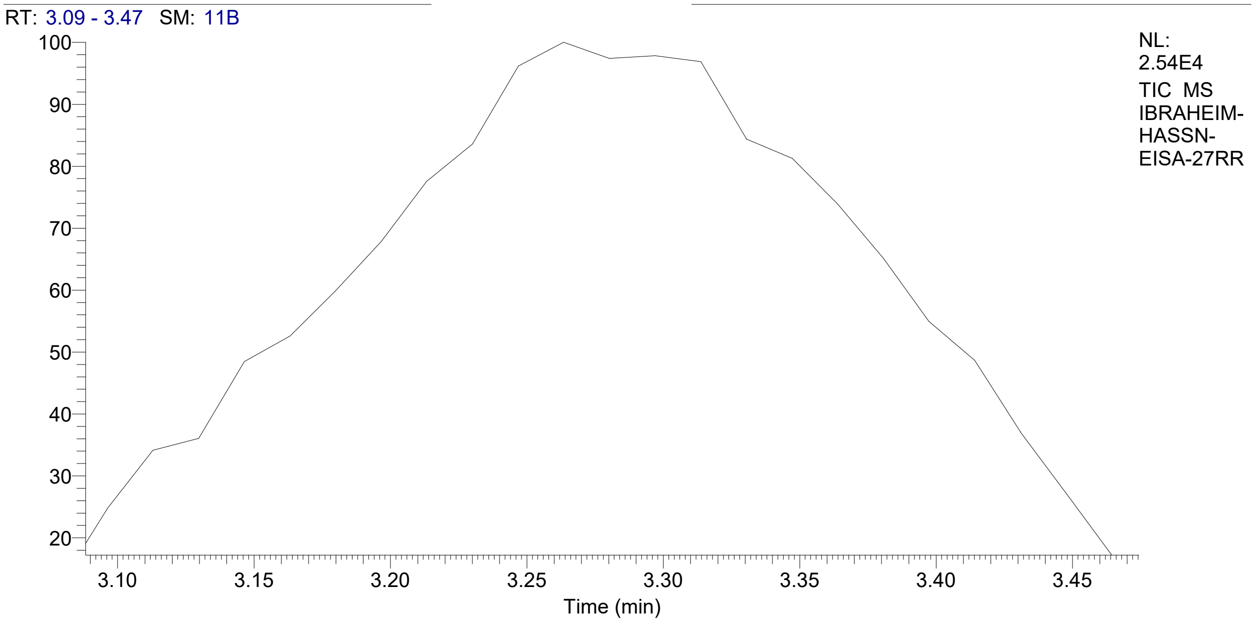

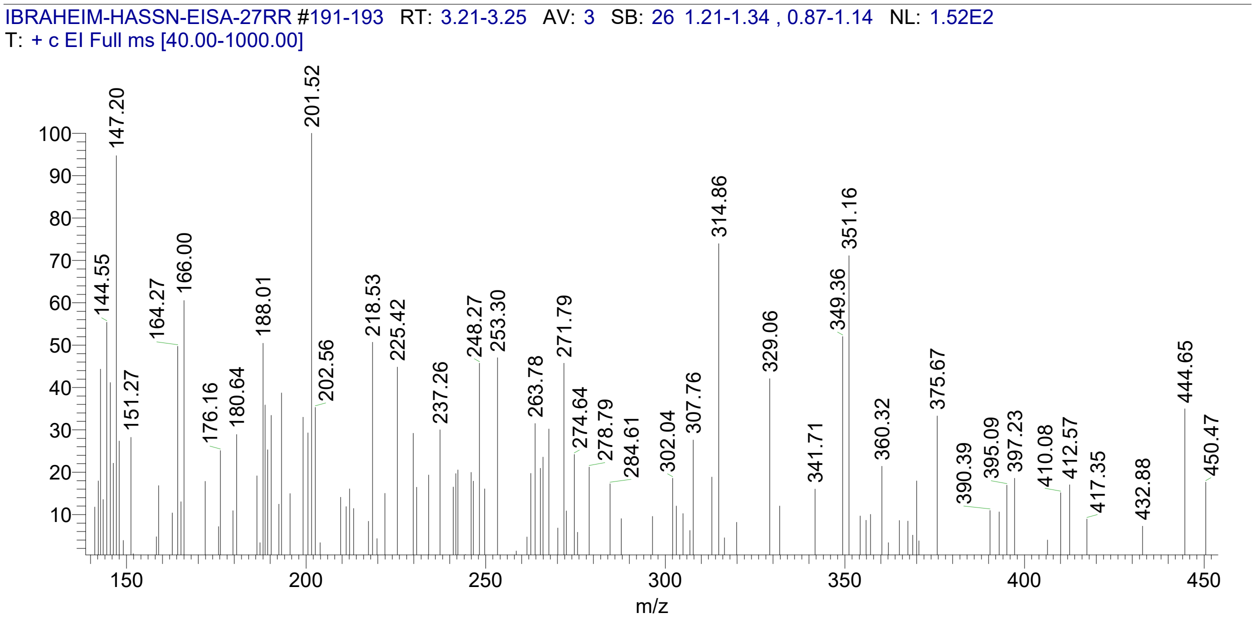

m/z	Intensity	Relative
141.16	17.9	11.77
142.13	27.3	17.96
142.75	67.4	44.32
143.53	20.6	13.55

144.55	84.2	55.37
145.47	62.6	41.16
146.33	33.6	22.12
147.20	144.0	94.72
147.95	41.6	27.34
149.10	5.9	3.89
151.27	42.9	28.21
151.88	1.2	0.76
158.32	7.3	4.78
158.95	25.6	16.84
162.75	15.8	10.39
164.27	75.6	49.72
165.20	19.8	13.04
166.00	92.0	60.55
171.88	27.1	17.86
175.63	10.9	7.16
176.16	38.2	25.11
179.63	16.6	10.93
180.64	43.9	28.88
186.35	29.1	19.14
187.15	5.1	3.34
188.01	76.6	50.42
188.57	54.5	35.87
189.33	38.5	25.30
190.28	50.8	33.41

192.44	18.9	12.47
193.16	58.9	38.73
195.57	22.7	14.95
199.17	50.2	33.01
200.53	44.5	29.27
201.52	152.0	100.00
202.56	53.7	35.31
203.90	5.1	3.37
209.61	21.4	14.10
211.17	18.0	11.85
212.14	24.4	16.04
213.24	17.4	11.46
217.38	12.8	8.40
218.53	77.0	50.66
219.73	6.6	4.34
221.94	22.8	15.00
225.42	68.1	44.81
229.83	44.4	29.20
230.76	25.0	16.43
234.11	29.4	19.33
237.26	45.6	30.03
240.99	25.1	16.50
241.72	29.9	19.66
242.28	31.2	20.55
244.31	0.8	0.50

245.97	30.3	19.96
246.58	27.2	17.86
248.27	69.6	45.75
249.75	24.4	16.06
253.30	71.5	47.05
258.52	2.1	1.40
261.47	7.1	4.69
262.56	30.0	19.73
263.78	47.9	31.52
265.22	31.8	20.90
265.95	35.8	23.55
267.63	45.9	30.20
270.08	10.4	6.81
271.79	69.5	45.73
272.47	16.5	10.86
274.64	36.8	24.19
275.60	8.9	5.83
278.79	32.2	21.21
284.61	26.3	17.28
287.73	13.7	9.03
296.42	14.5	9.56
302.04	28.2	18.55
303.05	18.2	12.00
304.91	15.6	10.26
306.84	9.5	6.26

307.76	41.9	27.59
312.94	28.6	18.82
314.86	112.4	73.94
316.44	6.8	4.50
319.85	12.5	8.19
329.06	64.0	42.10
331.86	18.3	12.04
341.71	24.3	16.00
349.36	79.1	52.02
351.16	108.1	71.11
354.26	14.7	9.65
355.88	13.2	8.67
357.16	15.2	10.03
360.32	32.5	21.37
362.12	5.1	3.37
365.19	13.1	8.59
367.52	12.8	8.44
368.89	7.8	5.16
370.00	27.2	17.92
370.59	5.7	3.78
375.67	50.6	33.27
390.39	16.7	10.96
392.95	16.2	10.64
395.09	25.8	16.94
397.23	28.2	18.58

406.39	6.1	4.00
410.08	23.1	15.16
412.57	25.9	17.01
417.35	13.6	8.92
432.88	11.0	7.25
444.65	53.1	34.95
450.47	26.8	17.64

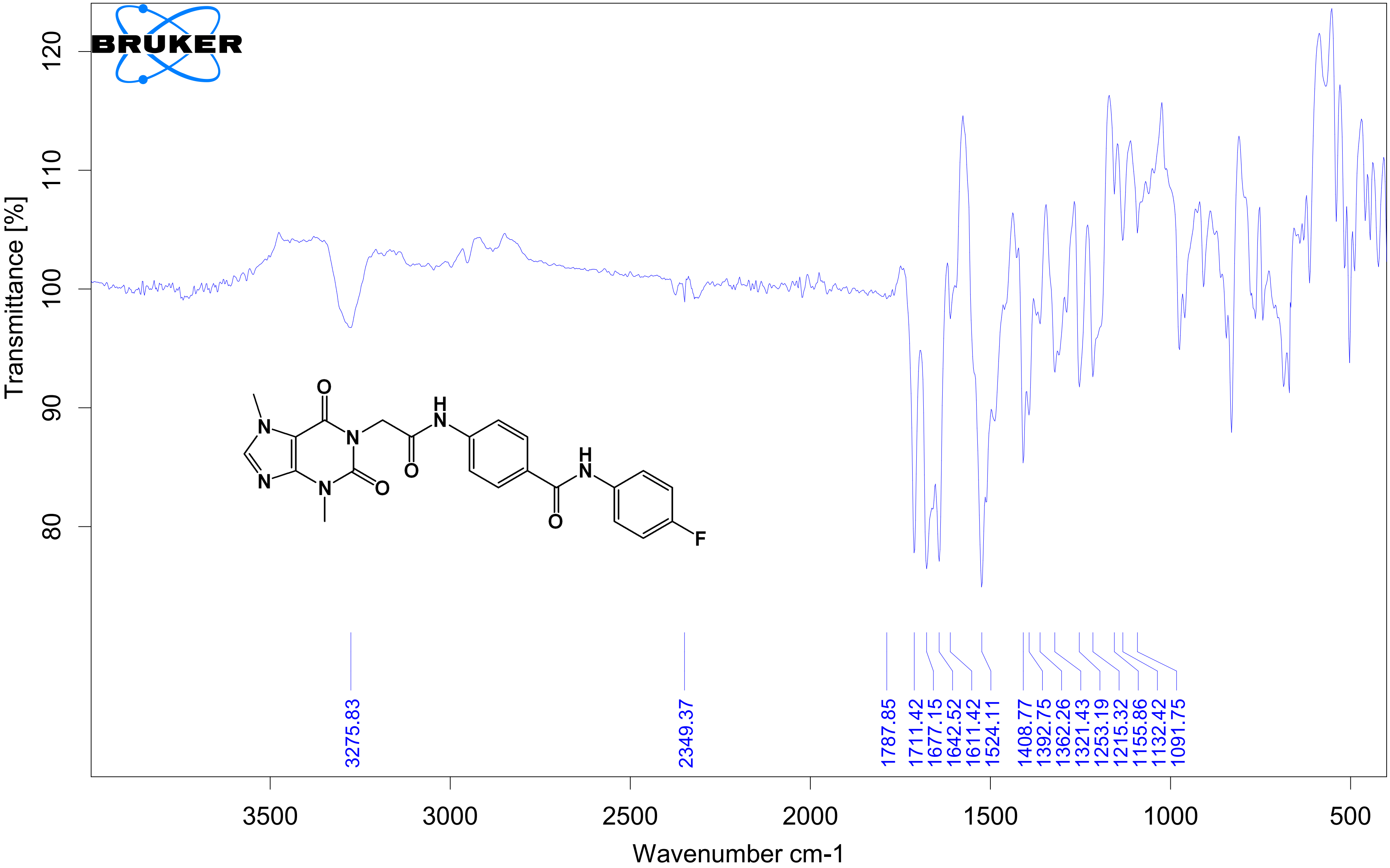

C:\Users\Public\Documents\Bruker\OPUS_8.1.29\DATA\MEAS\27RR.0	27RR	Instrument type and / or accessory	2/28/2023

Ibrahim Eissa-27RR-Hnmr-RR.10.fid
Ibrahim Eissa-27RR-Hnmr-RR

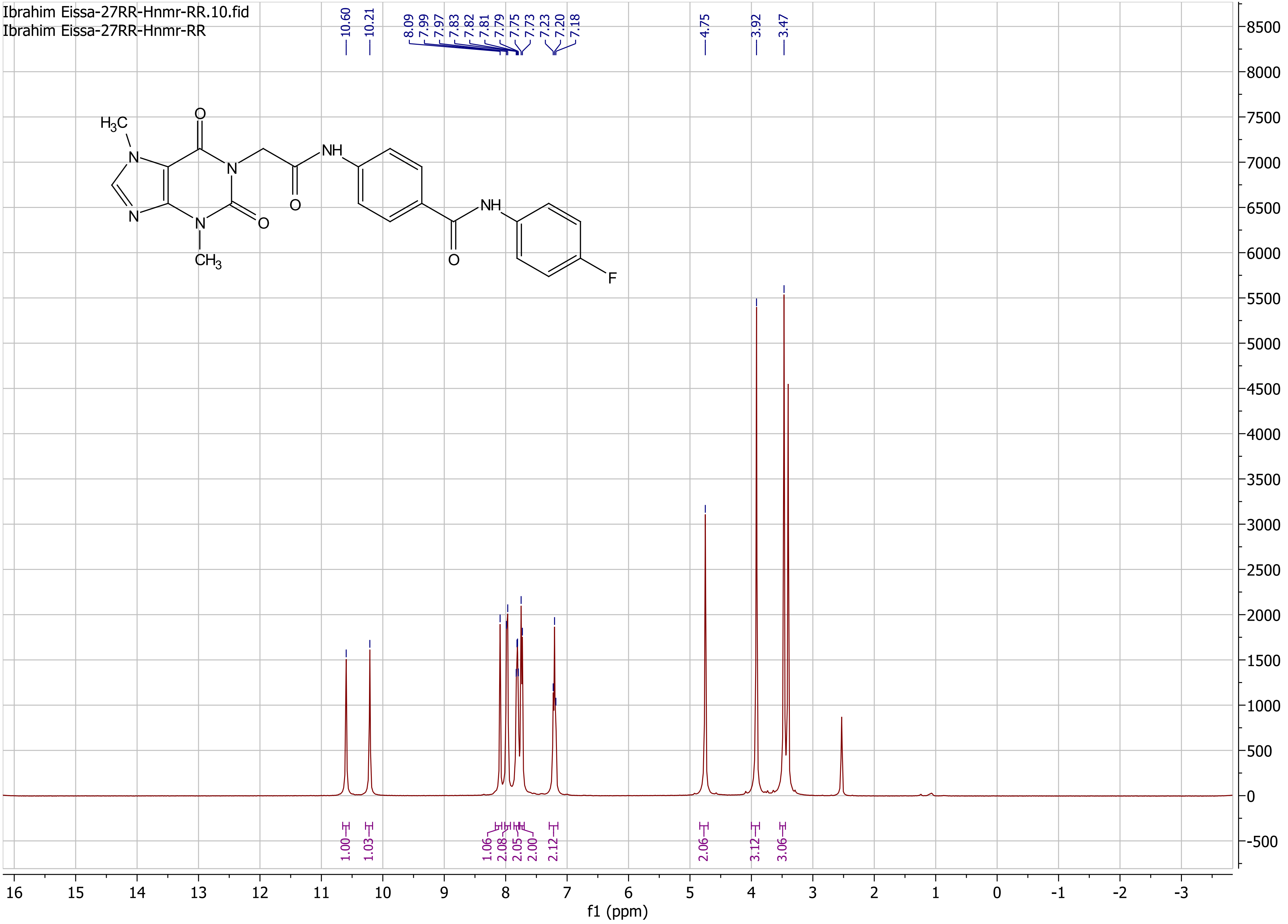

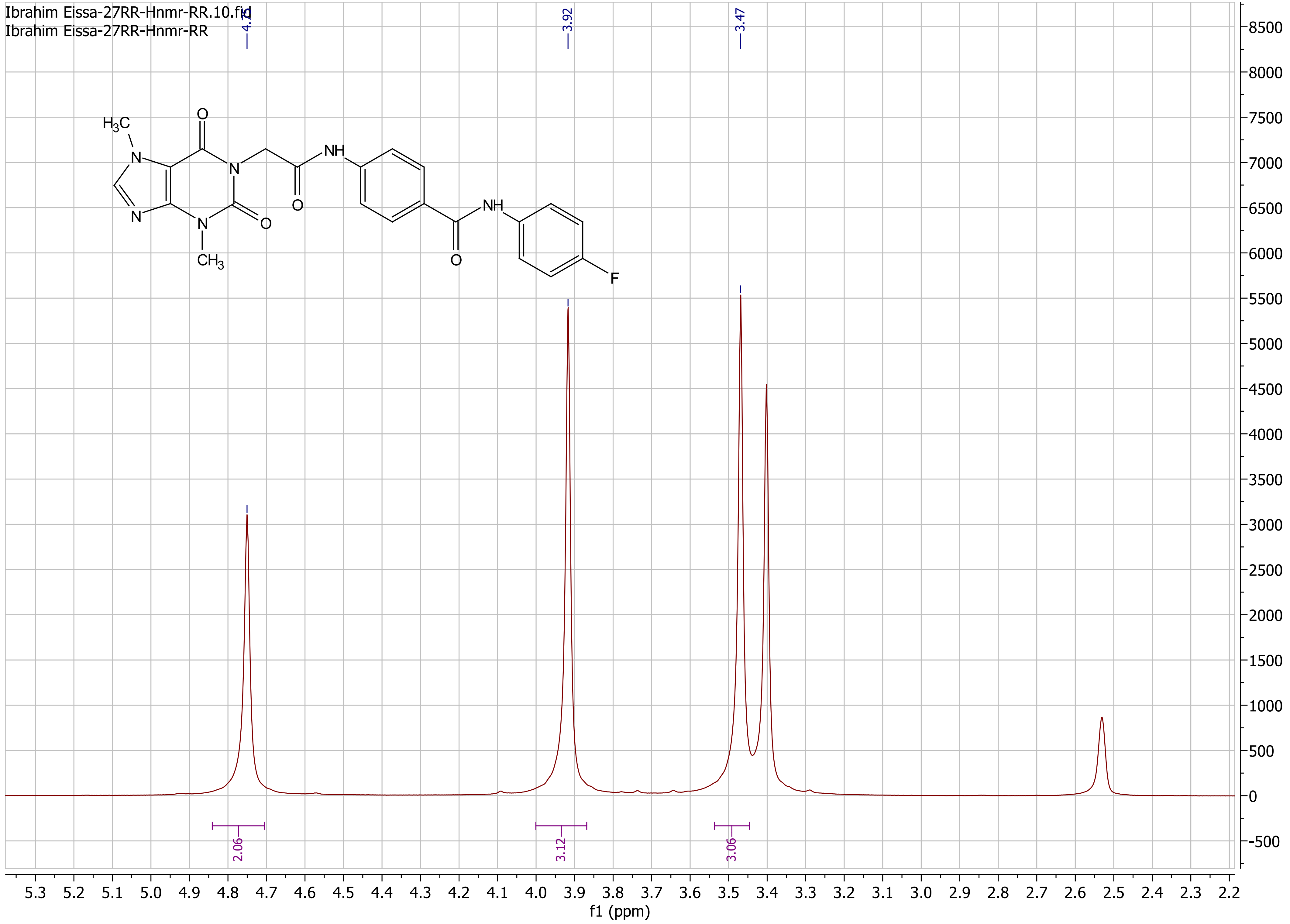
Ibrahim Eissa-27RR-Hnmr-RR.10.fid
Ibrahim Eissa-27RR-Hnmr-RR
4.75
3.92
3.47
2.06
3.12
3.06
f1 (ppm)

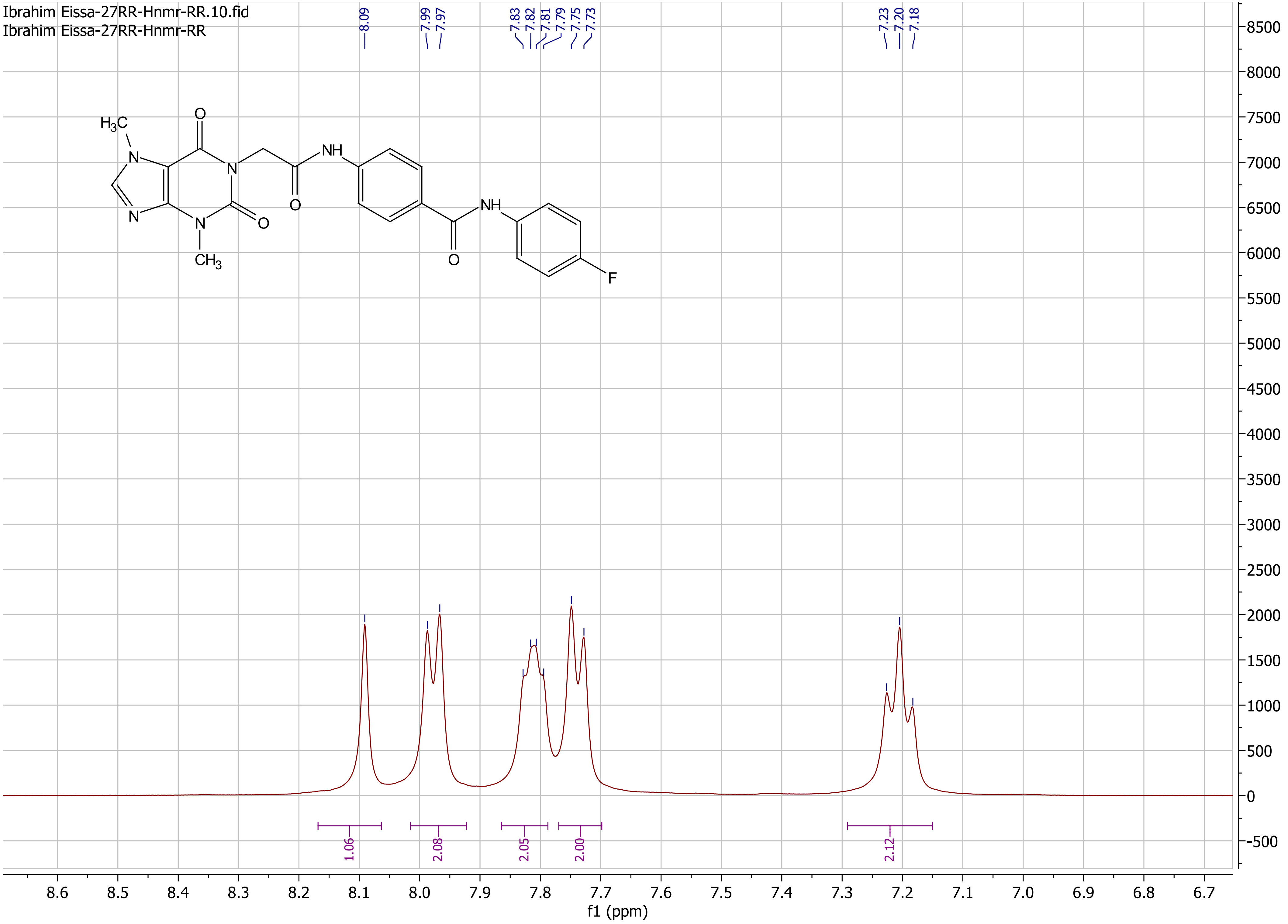
Ibrahim Eissa-27RR-Hnmr-RR.10.fid
Ibrahim Eissa-27RR-Hnmr-RR
8.09
7.99
7.97
7.83
7.82
7.81
7.79
7.75
7.73
7.23
7.20
7.18
H3C
O
N
N
N
N
O
CH3
O
NH
NH
O
F
1.06
2.08
2.05
2.00
2.12
f1 (ppm)

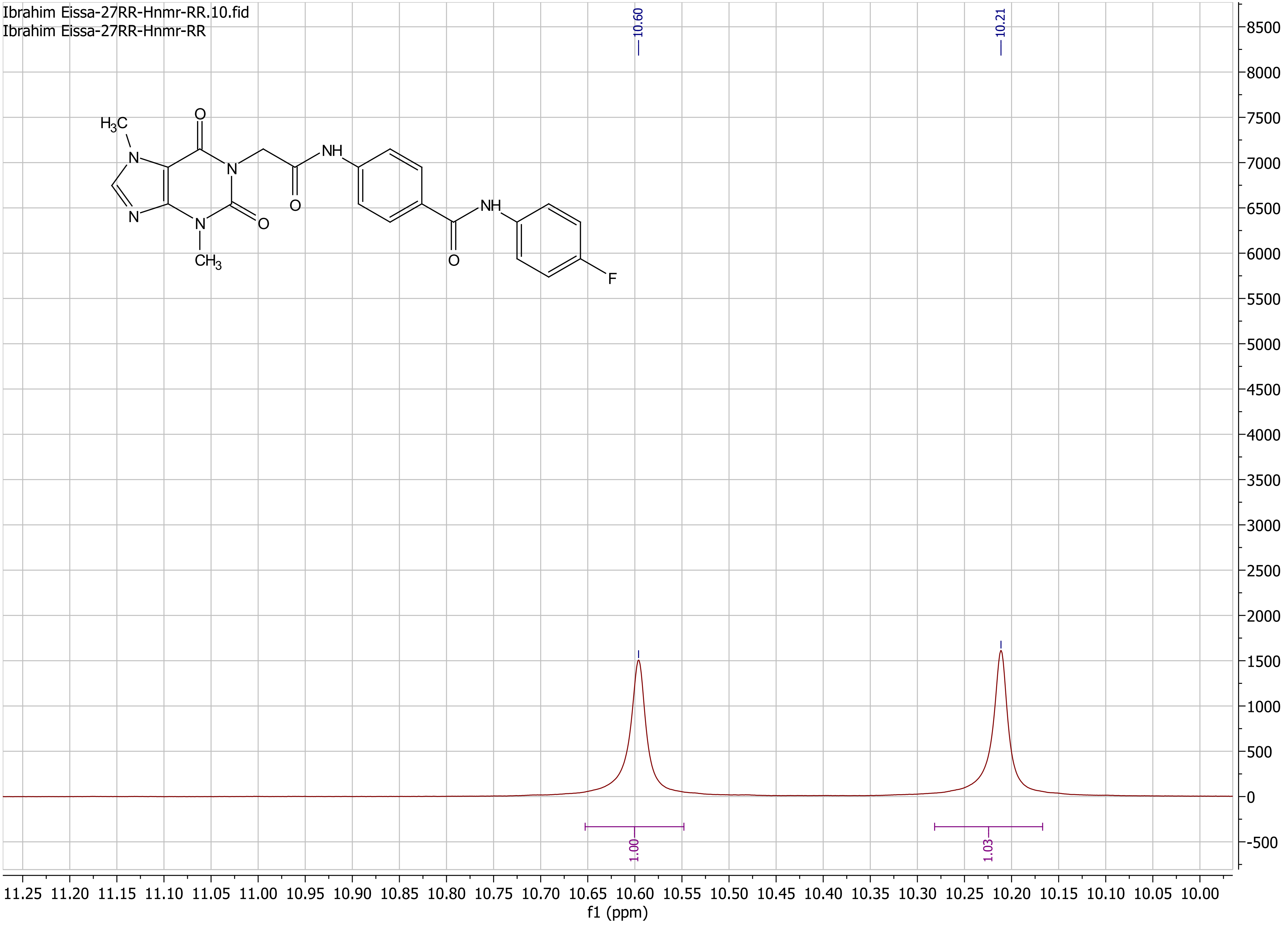
Ibrahim Eissa-27RR-Hnmr-RR.10.fid
Ibrahim Eissa-27RR-Hnmr-RR
10.60
10.21
1.00
1.03
11.25 11.20 11.15 11.10 11.05 11.00 10.95 10.90 10.85 10.80 10.75 10.70 10.65 10.60 10.55 10.50 10.45 10.40 10.35 10.30 10.25 10.20 10.15 10.10 10.05 10.00
f1 (ppm)
8500 8000 7500 7000 6500 6000 5500 5000 4500 4000 3500 3000 2500 2000 1500 1000 500 0 -500

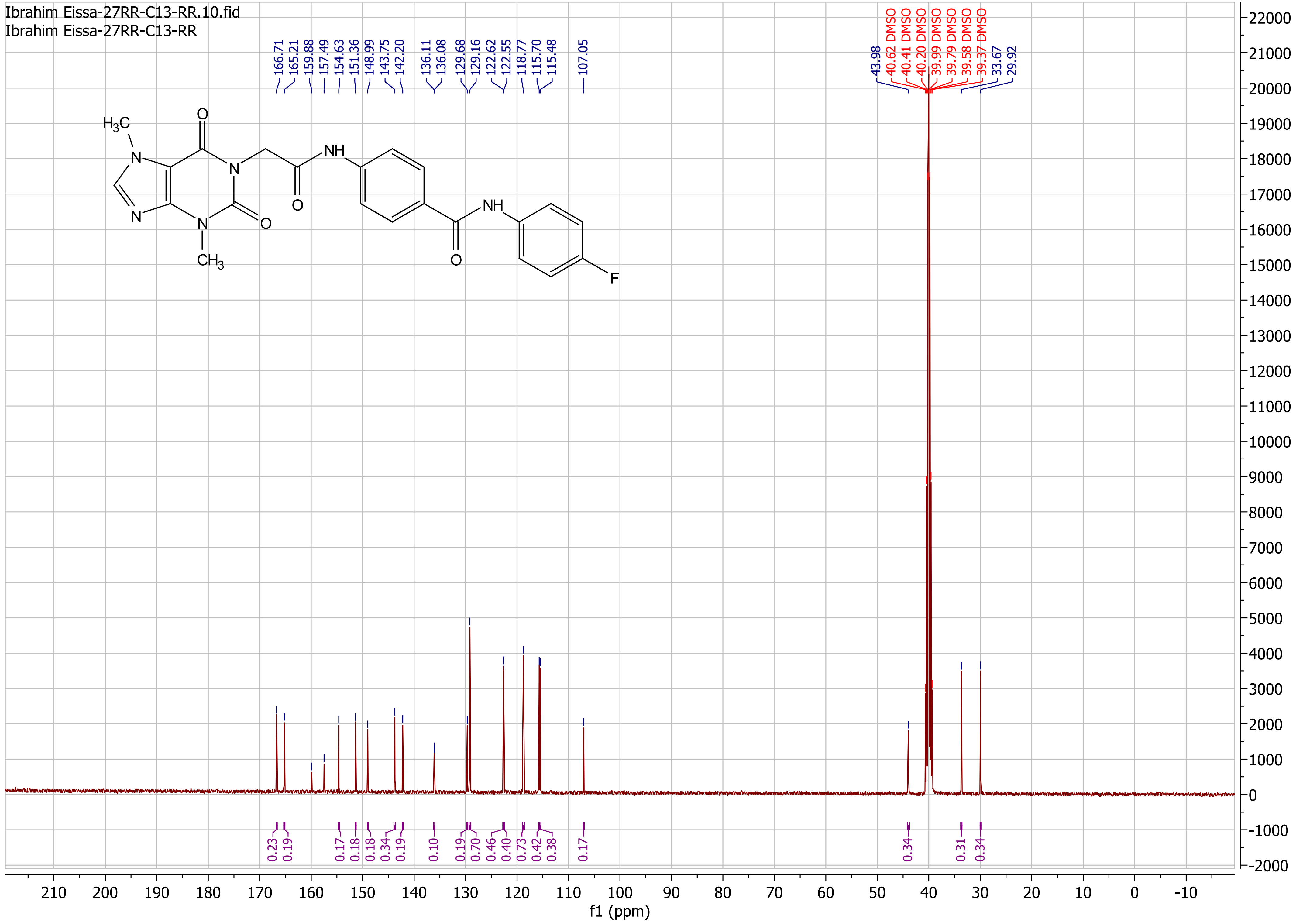

Ibrahim Eissa-27RR-C13-RR.10.fid
Ibrahim Eissa-27RR-C13-RR
166.71
165.21
159.88
157.49
154.63
151.36
148.99
143.75
142.20
136.11
136.08
129.68
129.16
122.62
122.55
118.77
115.70
115.48
107.05
43.98
40.62 DMSO
40.41 DMSO
40.20 DMSO
39.99 DMSO
39.79 DMSO
39.58 DMSO
39.37 DMSO
33.67
29.92
f1 (ppm)

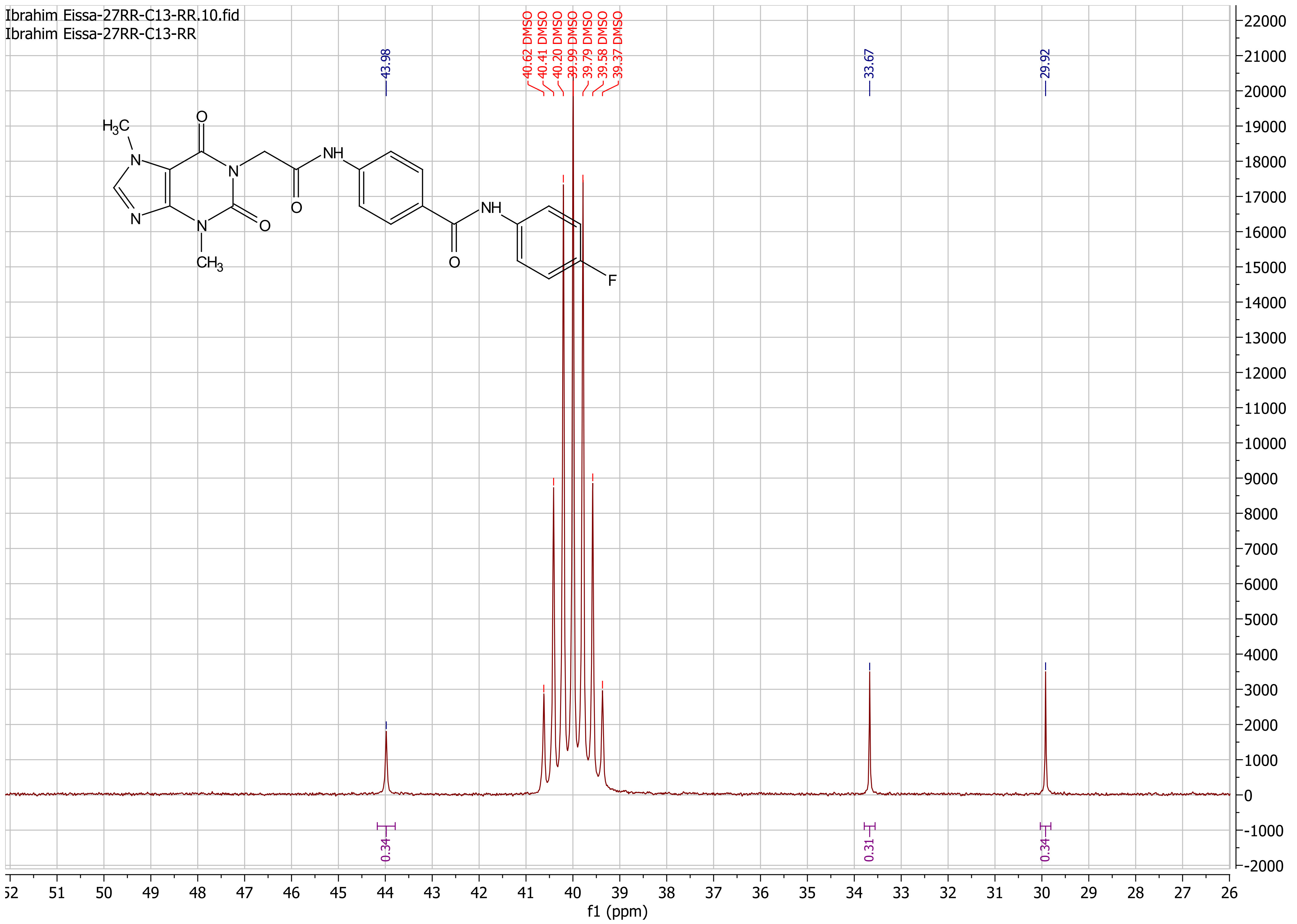
Ibrahim Eissa-27RR-C13-RR.10.fid
Ibrahim Eissa-27RR-C13-RR
43.98
40.62 DMSO
40.41 DMSO
40.20 DMSO
39.99 DMSO
39.79 DMSO
39.58 DMSO
39.37 DMSO
33.67
29.92
0.34
0.31
0.34
f1 (ppm)

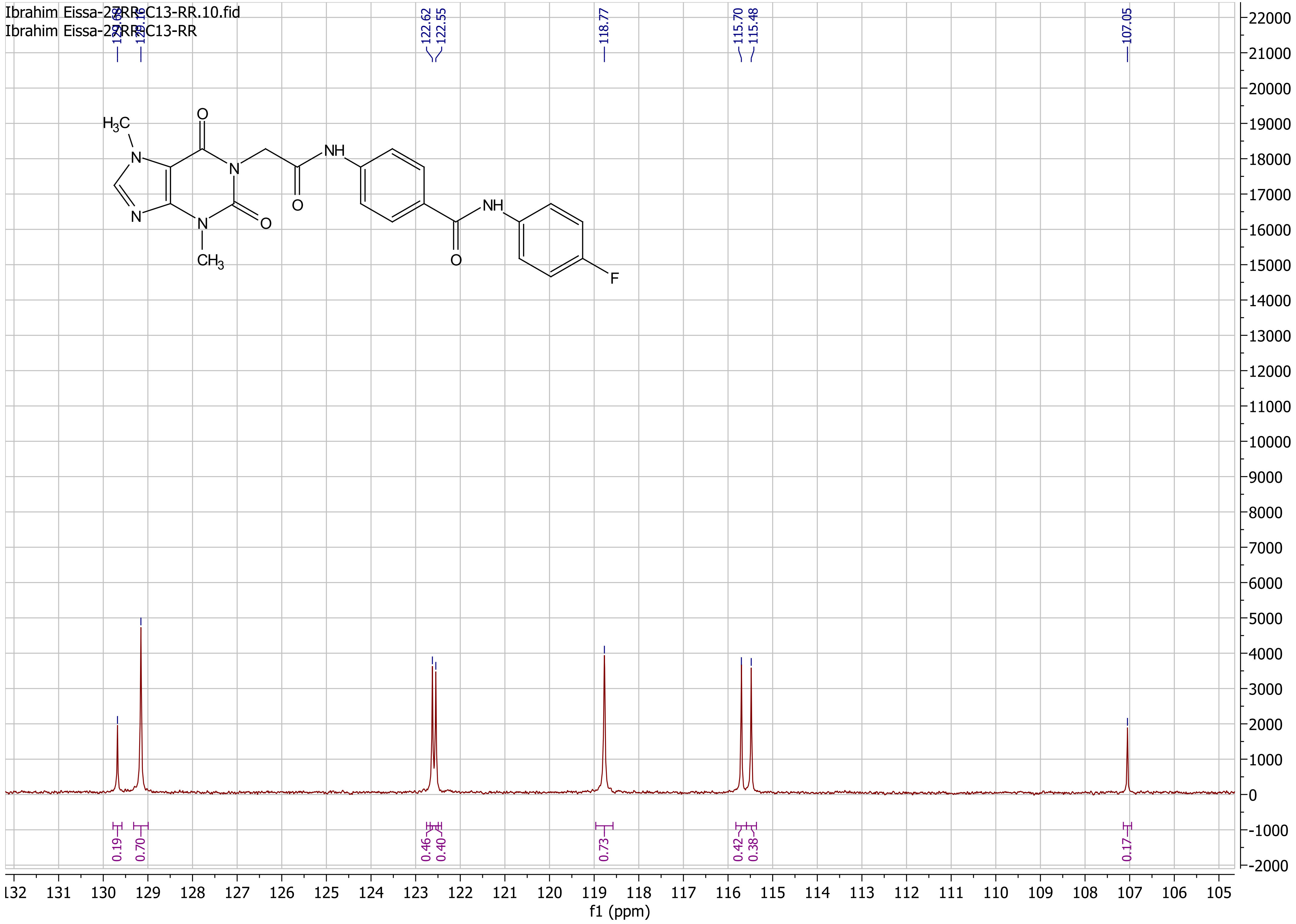
Ibrahim Eissa-27RR-C13-RR.10.fid
Ibrahim Eissa-27RR-C13-RR
129.68
129.16
122.62
122.55
118.77
115.70
115.48
107.05
0.19
0.70
0.46
0.40
0.73
0.42
0.38
0.17
f1 (ppm)

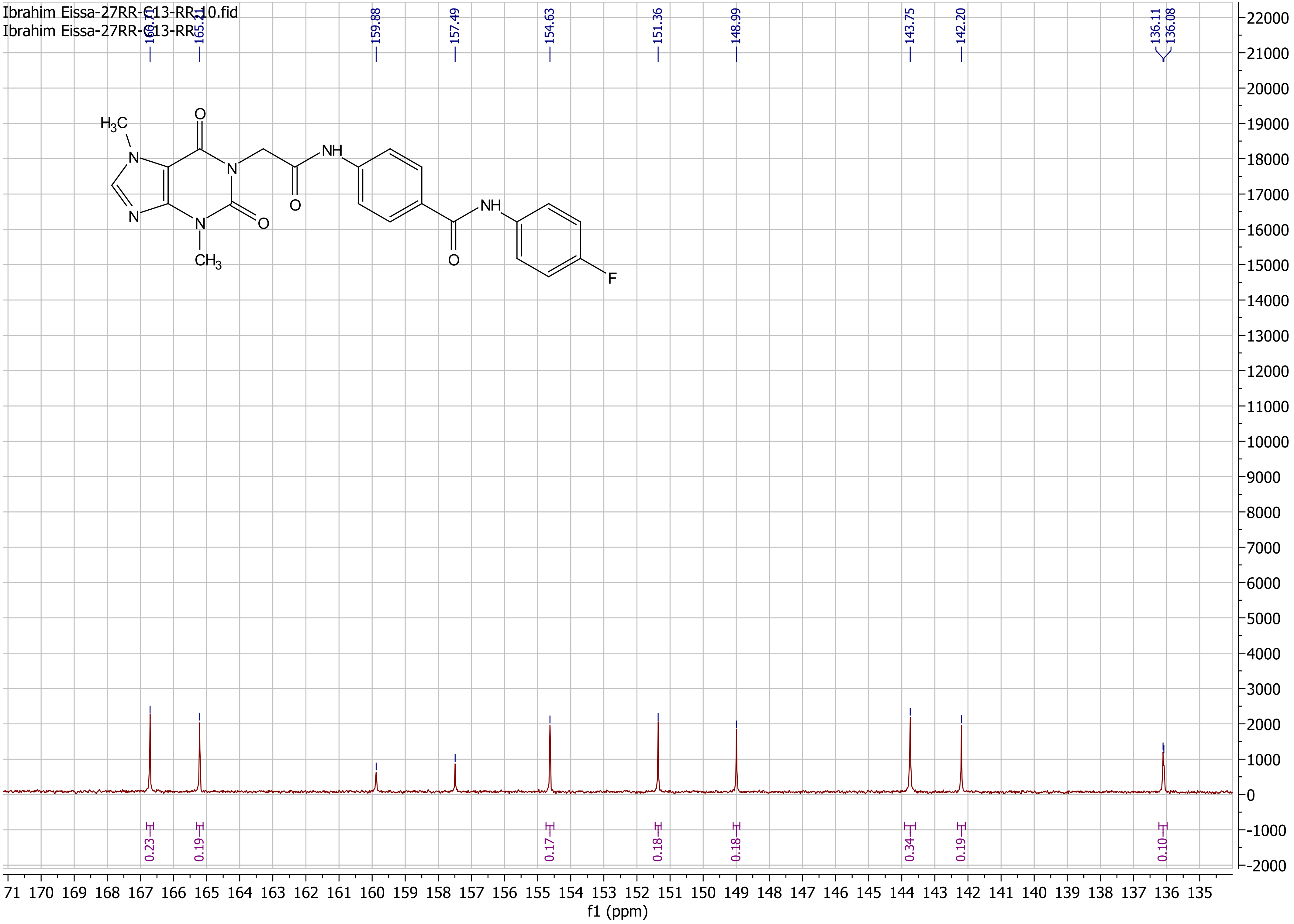

Ibrahim Eissa-27RR-C13-RR-10.fid
Ibrahim Eissa-27RR-C13-RR
159.88
157.49
154.63
151.36
148.99
143.75
142.20
136.11
136.08
H_3C
N
O
N
NH
O
N
N
O
CH_3
NH
O
F
0.23
0.19
0.17
0.18
0.18
0.34
0.19
0.10
f1 (ppm)

Toxicity Report

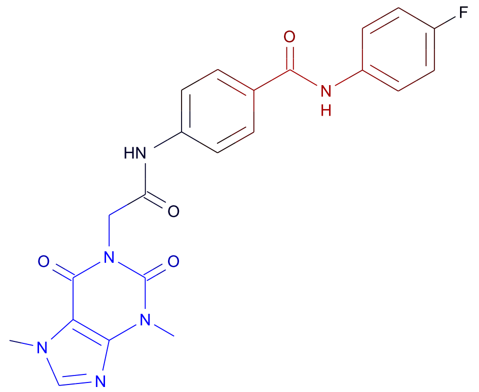

$C_{22}H_{19}FN_6O_4$
Molecular Weight: 450.42246
ALogP: 1.526
Rotatable Bonds: 5
Acceptors: 5
Donors: 2

Structural Similar Compounds

Name	67450-45-7	Delavirdine	34433-31-3
Structure	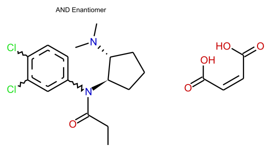	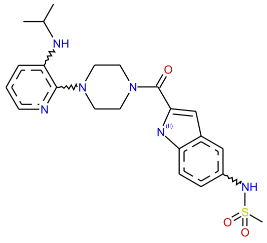	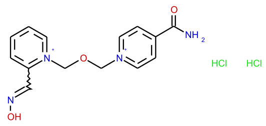
Actual Endpoint	Non-Mutagen	Non-Mutagen	Non-Mutagen
Predicted Endpoint	Non-Mutagen	Non-Mutagen	Non-Mutagen
Distance	0.568	0.616	0.630
Reference	Kazius et. al., J. Med. Chem. (2005) 48, 312-320	Contrera, J.F., Matthews, E.J., Kruhlak, N.L., and Benz, R.D., Regulatory Toxicology and Pharmacology 2005, 313-323.	Kazius et. al., J. Med. Chem. (2005) 48, 312-320

Model Prediction

Prediction: Non-Mutagen
Probability: 0.421
Enrichment: 0.754
Bayesian Score: -9.24
Mahalanobis Distance: 13.8
Mahalanobis Distance p-value: 3.03e-008

Prediction: Positive if the Bayesian score is above the estimated best cutoff value from minimizing the false positive and false negative rate.
Probability: The esimated probability that the sample is in the positive category. This assumes that the Bayesian score follows a normal distribution and is different from the prediction using a cutoff.
Enrichment: An estimate of enrichment, that is, the increased likelihood (versus random) of this sample being in the category.
Bayesian Score: The standard Laplacian-modified Bayesian score.
Mahalanobis Distance: The Mahalanobis distance (MD) is the distance to the center of the training data. The larger the MD, the less trustworthy the prediction.
Mahalanobis Distance p-value: The p-value gives the fraction of training data with an MD greater than or equal to the one for the given sample, assuming normally distributed data. The smaller the p-value, the less trustworthy the prediciton. For highly non-normal X properties (e.g., fingerprints), the MD p-value is wildly inaccurate.

Model Applicability

Unknown features are fingerprint features in the query molecule, but not found or appearing too infrequently in the training set.

1. All properties and OPS components are within expected ranges.

Feature Contribution

Top features for positive contribution				
**Fingerprint**	**Bit/Smiles**	**Feature Structure**	**Score**	**Mutagen in training set**
SCFP_12	136358998	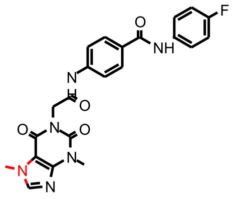 [*]:n(:[*])C	0.455	142 out of 157

SCFP_12	555539852	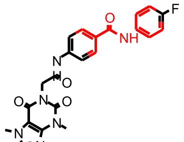 [*]:[cH]:[c](:[cH]:[*])C(=O)N[c]1:[cH]:[cH]:[*]:[cH]:[cH]:1	0.447	22 out of 24
SCFP_12	818445224	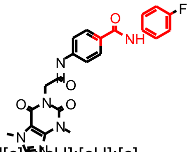 [*][c]1:[cH]:[cH]:[c](NC(=O)[c](:[*]):[*]):[cH]:[cH]:1	0.434	12 out of 13
**Top Features for negative contribution**				
**Fingerprint**	**Bit/Smiles**	**Feature Structure**	**Score**	**Mutagen in training set**
SCFP_12	1205795299	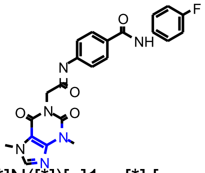 [*]N([*])[c]1:n:[*]:[*]:[c]:1[*]	-1.22	2 out of 16
SCFP_12	1731225349	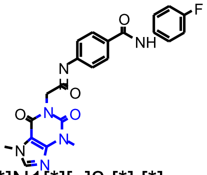 [*]N1[*][c]2:[*]:[*]:n:[c]:2N(C)C1=O	-1.19	0 out of 4
SCFP_12	1445006032	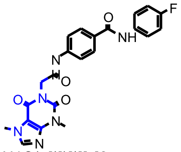 [*]CN1C(=[*])[*][c]2:[*]:[*]:n([*]):[c]:2C1=O	-1.19	0 out of 4

Sorafenib

TOPKAT_Ames_Mutagenicity

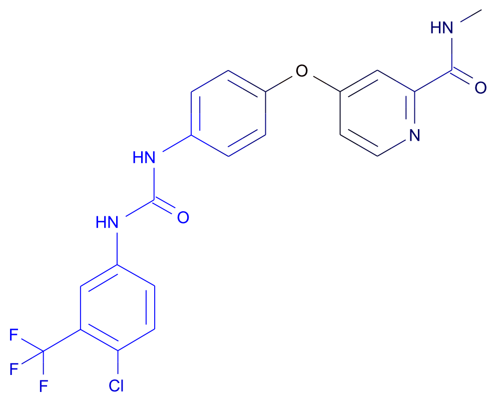

$C_{21}H_{16}ClF_3N_4O_3$
Molecular Weight: 464.82494
ALogP: 4.175
Rotatable Bonds: 6
Acceptors: 4
Donors: 3

Structural Similar Compounds

Name	GLYBURIDE	38914-96-4	93957-54-1
Structure	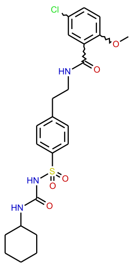	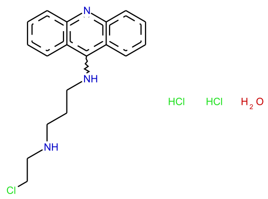	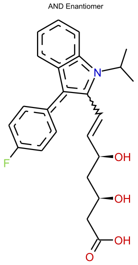
Actual Endpoint	Non-Mutagen	Mutagen	Non-Mutagen
Predicted Endpoint	Non-Mutagen	Mutagen	Non-Mutagen
Distance	0.590	0.592	0.600
Reference	PDR 1994	Kazius et. al., J. Med. Chem. (2005) 48, 312-320	US Environmental Protection Agency at http://www.epa.gov/NCCT/dsstox/sdf_isscan_external.html

Model Prediction

Prediction: Non-Mutagen
Probability: 0.0531
Enrichment: 0.0951
Bayesian Score: -19.7
Mahalanobis Distance: 13.1
Mahalanobis Distance p-value: 2.73e-006

Prediction: Positive if the Bayesian score is above the estimated best cutoff value from minimizing the false positive and false negative rate.
Probability: The esimated probability that the sample is in the positive category. This assumes that the Bayesian score follows a normal distribution and is different from the prediction using a cutoff.
Enrichment: An estimate of enrichment, that is, the increased likelihood (versus random) of this sample being in the category.
Bayesian Score: The standard Laplacian-modified Bayesian score.
Mahalanobis Distance: The Mahalanobis distance (MD) is the distance to the center of the training data. The larger the MD, the less trustworthy the prediction.
Mahalanobis Distance p-value: The p-value gives the fraction of training data with an MD greater than or equal to the one for the given sample, assuming normally distributed data. The smaller the p-value, the less trustworthy the prediciton. For highly non-normal X properties (e.g., fingerprints), the MD p-value is wildly inaccurate.

Model Applicability

Unknown features are fingerprint features in the query molecule, but not found or appearing too infrequently in the training set.

1. All properties and OPS components are within expected ranges.

Feature Contribution

Top features for positive contribution				
**Fingerprint**	**Bit/Smiles**	**Feature Structure**	**Score**	**Mutagen in training set**
SCFP_12	-347281112	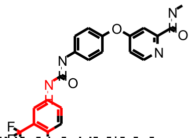 [*]N[c]1:[cH]:[*]:[c]([*]):[c](:[cH]:1)C([*])([*])[*]	0.337	18 out of 22

SCFP_12	1208843554	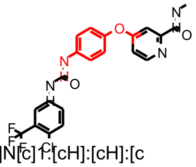 [*]N[c]1:[cH]:[cH]:[c](O[c](:[*]):[*]):[cH]:[cH]:1	0.337	6 out of 7
SCFP_12	-1943080297	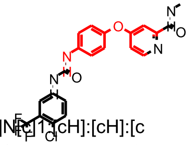 [*]N[c]1:[cH]:[cH]:[c](O[c]2:[cH]:[cH]:[*]:[c]([*]):[cH]:2):[cH]:[cH]:1	0.304	5 out of 6

Top Features for negative contribution

Fingerprint	Bit/Smiles	Feature Structure	Score	Mutagen in training set
SCFP_12	816802409	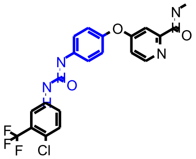 [*]NC(=O)N[c]1:[cH]:[cH]:[c]([*]):[cH]:[cH]:1	-1.82	0 out of 9
SCFP_12	-300280774	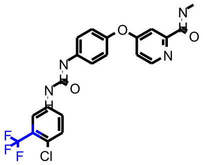 [*]:[c](:[*])C(F)(F)F	-1.51	3 out of 30
SCFP_12	-1903175541	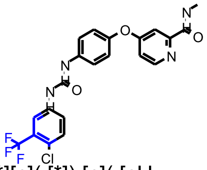 [*][c](:[*]):[c](:[cH]:[*])C(F)(F)F	-1.51	3 out of 30

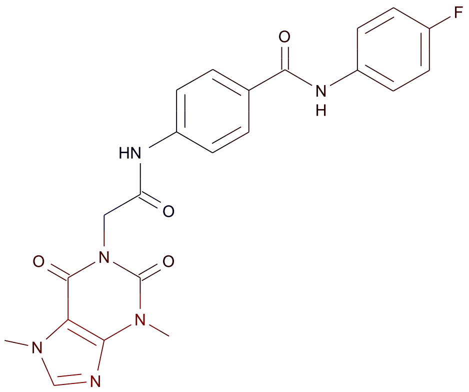

$C_{22}H_{19}FN_6O_4$

Molecular Weight: 450.42246

ALogP: 1.526

Rotatable Bonds: 5

Acceptors: 5

Donors: 2

Structural Similar Compounds

Name	Tiaramide .HCl (Free base form)	Citreoviridin	Domperidone
Structure	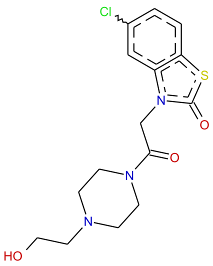	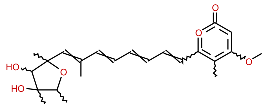	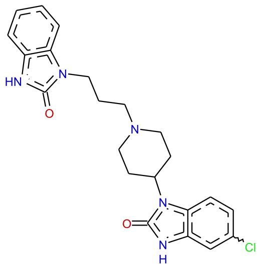
Actual Endpoint	Toxic	Toxic	Toxic
Predicted Endpoint	Toxic	Toxic	Toxic
Distance	0.641	0.649	0.651
Reference	Arzneimittelforschung 23(4):504-8; 1973	Food Chem Toxicol 24(12):1315-20; 1986	Yakuri to Chiryo 8:4125-4136; 1980

Model Prediction

Prediction: Toxic

Probability: 0.714

Enrichment: 1.36

Bayesian Score: 4.27

Mahalanobis Distance: 8.13

Mahalanobis Distance p-value: 0.545

Prediction: Positive if the Bayesian score is above the estimated best cutoff value from minimizing the false positive and false negative rate.
Probability: The esimated probability that the sample is in the positive category. This assumes that the Bayesian score follows a normal distribution and is different from the prediction using a cutoff.
Enrichment: An estimate of enrichment, that is, the increased likelihood (versus random) of this sample being in the category.
Bayesian Score: The standard Laplacian-modified Bayesian score.
Mahalanobis Distance: The Mahalanobis distance (MD) is the distance to the center of the training data. The larger the MD, the less trustworthy the prediction.
Mahalanobis Distance p-value: The p-value gives the fraction of training data with an MD greater than or equal to the one for the given sample, assuming normally distributed data. The smaller the p-value, the less trustworthy the prediciton. For highly non-normal X properties (e.g., fingerprints), the MD p-value is wildly inaccurate.

Model Applicability

Unknown features are fingerprint features in the query molecule, but not found or appearing too infrequently in the training set.

1. All properties and OPS components are within expected ranges.

Feature Contribution

Top features for positive contribution

Fingerprint	Bit/Smiles	Feature Structure	Score	Toxic in training set
SCFP_6	282594097	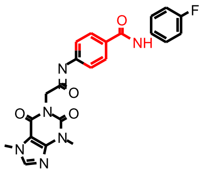 [*]NC(=O)[c]1:[cH]:[cH]:[*]:[cH]:[cH]:1	0.441	3 out of 3

SCFP_6	1257084377	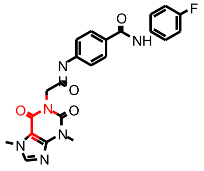 [*]N([*])C(=O)[c](:[*]):[*]	0.362	14 out of 18
SCFP_6	-783770208	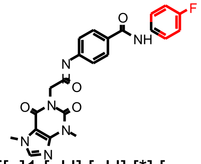 F[c]1:[cH]:[cH]:[*]:[cH]:[cH]:1	0.322	4 out of 5

Top Features for negative contribution

Fingerprint	Bit/Smiles	Feature Structure	Score	Toxic in training set
SCFP_6	136358998	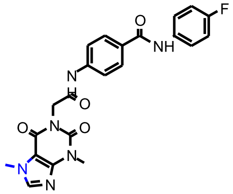 [*]:n(:[*])C	-0.55	2 out of 8
SCFP_6	399659969	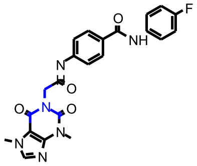 [*]CN(C(=[*])[*])C(=[*])[*]	-0.526	3 out of 11
SCFP_6	2097618059	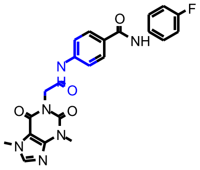 [*]CC(=O)N[c](:[cH]:[*]):[cH]:[*]	-0.422	0 out of 1

Sorafenib

TOPKAT_Developmental_Toxicity_Potential

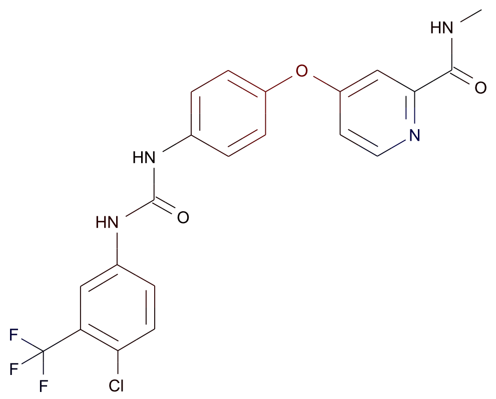

$C_{21}H_{16}ClF_3N_4O_3$

Molecular Weight: 464.82494

ALogP: 4.175

Rotatable Bonds: 6

Acceptors: 4

Donors: 3

Model Prediction

Prediction: Toxic

Probability: 0.592

Enrichment: 1.13

Bayesian Score: 1.15

Mahalanobis Distance: 12.6

Mahalanobis Distance p-value: 2.07e-006

Prediction: Positive if the Bayesian score is above the estimated best cutoff value from minimizing the false positive and false negative rate.
Probability: The esimated probability that the sample is in the positive category. This assumes that the Bayesian score follows a normal distribution and is different from the prediction using a cutoff.
Enrichment: An estimate of enrichment, that is, the increased likelihood (versus random) of this sample being in the category.
Bayesian Score: The standard Laplacian-modified Bayesian score.
Mahalanobis Distance: The Mahalanobis distance (MD) is the distance to the center of the training data. The larger the MD, the less trustworthy the prediction.
Mahalanobis Distance p-value: The p-value gives the fraction of training data with an MD greater than or equal to the one for the given sample, assuming normally distributed data. The smaller the p-value, the less trustworthy the prediciton. For highly non-normal X properties (e.g., fingerprints), the MD p-value is wildly inaccurate.

Structural Similar Compounds

Name	Chenodiol	Amsacrine	Ochratoxin a
Structure	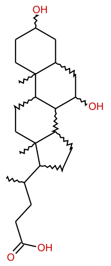	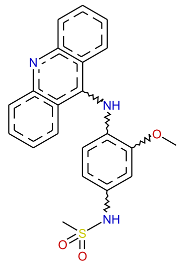	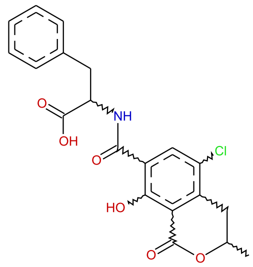
Actual Endpoint	Toxic	Toxic	Toxic
Predicted Endpoint	Toxic	Toxic	Toxic
Distance	0.631	0.637	0.644
Reference	Arch Int Pharm 246:149-158; 1980	Fundam Appl Toxicol 7(2):214-20; 1986	Toxicol Appl Pharmacol 37(2):331-8; 1976

Model Applicability

Unknown features are fingerprint features in the query molecule, but not found or appearing too infrequently in the training set.

1. All properties and OPS components are within expected ranges.

Feature Contribution

Top features for positive contribution

Fingerprint	Bit/Smiles	Feature Structure	Score	Toxic in training set
SCFP_6	1559190850	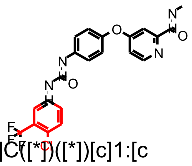 [*]C([*])([*])[c]1:[cH]:[*]:[cH]:[cH]:[c]:1Cl	0.441	3 out of 3

SCFP_6	-488587948	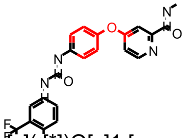 [*]:[c](:[*])O[c]1:[c<br>H]:[cH]:[*]:[cH]:[cH<br>]:1	0.381	2 out of 2
SCFP_6	-975241316	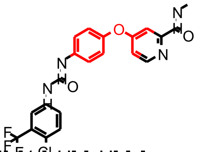 [*][c]1:[cH]:[cH]:[c]<br>(O[c](:[cH]:[*]):[cH<br>]:[*]):[cH]:[cH]:1	0.381	2 out of 2
**Top Features for negative contribution**				
**Fingerprint**	**Bit/Smiles**	**Feature Structure**	**Score**	**Toxic in training set**
SCFP_6	-1794974220	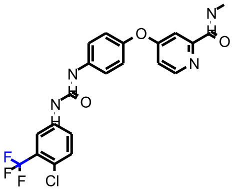 [*]C([*])([*])F	-0.55	2 out of 8
SCFP_6	-937094999	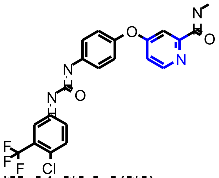 [*][c]1:[*]:[c]([*]):<br>n:[cH]:[cH]:1	-0.358	3 out of 9
SCFP_6	-496201075	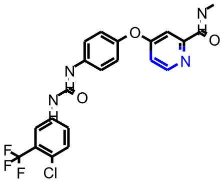 [*]:[cH]:[cH]:n:[*]	-0.289	8 out of 21

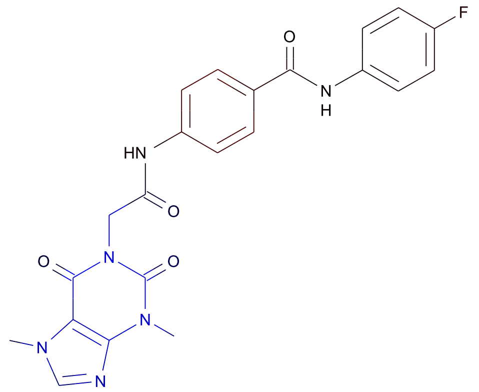

$C_{22}H_{19}FN_6O_4$
Molecular Weight: 450.42246
ALogP: 1.526
Rotatable Bonds: 5
Acceptors: 5
Donors: 2

Model Prediction

Prediction: Non-Carcinogen
Probability: 0.205
Enrichment: 0.64
Bayesian Score: -5.76
Mahalanobis Distance: 11.8
Mahalanobis Distance p-value: 0.0162

Prediction: Positive if the Bayesian score is above the estimated best cutoff value from minimizing the false positive and false negative rate.
Probability: The esimated probability that the sample is in the positive category. This assumes that the Bayesian score follows a normal distribution and is different from the prediction using a cutoff.
Enrichment: An estimate of enrichment, that is, the increased likelihood (versus random) of this sample being in the category.
Bayesian Score: The standard Laplacian-modified Bayesian score.
Mahalanobis Distance: The Mahalanobis distance (MD) is the distance to the center of the training data. The larger the MD, the less trustworthy the prediction.
Mahalanobis Distance p-value: The p-value gives the fraction of training data with an MD greater than or equal to the one for the given sample, assuming normally distributed data. The smaller the p-value, the less trustworthy the prediciton. For highly non-normal X properties (e.g., fingerprints), the MD p-value is wildly inaccurate.

Structural Similar Compounds

Name	Bicalutamide	Glipizide	Acetohexamide
Structure	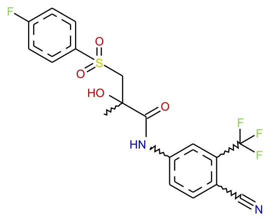	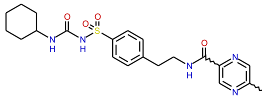	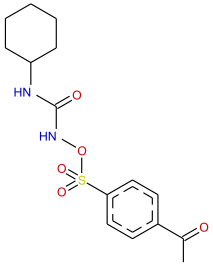
Actual Endpoint	Non-Carcinogen	Non-Carcinogen	Non-Carcinogen
Predicted Endpoint	Non-Carcinogen	Non-Carcinogen	Non-Carcinogen
Distance	0.577	0.674	0.677
Reference	US FDA (Centre for Drug Eval.& Res./Off. Testing & Res.) Sept. 1997	US FDA (Centre for Drug Eval.& Res./Off. Testing & Res.) Sept. 1997	US FDA (Centre for Drug Eval.& Res./Off. Testing & Res.) Sept. 1997

Model Applicability

Unknown features are fingerprint features in the query molecule, but not found or appearing too infrequently in the training set.

1. All properties and OPS components are within expected ranges.

Feature Contribution

Top features for positive contribution

Fingerprint	Bit/Smiles	Feature Structure	Score	Carcinogen in training set
ECFP_6	738938915	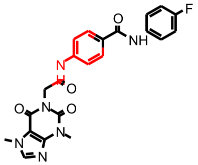 [*]C(=[*])N[c]1:[cH]:[cH]:[*]:[cH]:[cH]:1	0.617	2 out of 2

ECFP_6	-407983022	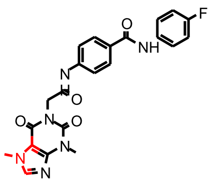<br>[*][c]1:[*]:[*]:[cH]:n:1C	0.442	2 out of 3
ECFP_6	-223149939	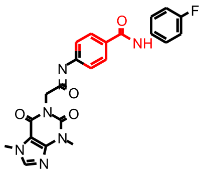<br>[*]NC(=O)[c]1:[cH]:[cH]:[*]:[cH]:[cH]:1	0.442	2 out of 3

Top Features for negative contribution

Fingerprint	Bit/Smiles	Feature Structure	Score	Carcinogen in training set
ECFP_6	-661097313	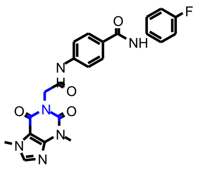<br>[*]CN(C(=[*])[*])C(=[*])[*]	-1.55	0 out of 12
ECFP_6	1731843802	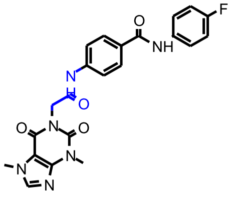<br>[*]CC(=O)N[*]	-0.657	0 out of 3
ECFP_6	-813242890	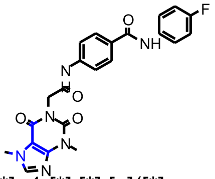<br>[*]n1:[*]:[*]:[c]([*]):[c]:1C(=[*])[*]	-0.482	0 out of 2

Sorafenib

TOPKAT_Mouse_Female_FDA_None_vs_Carcinogen

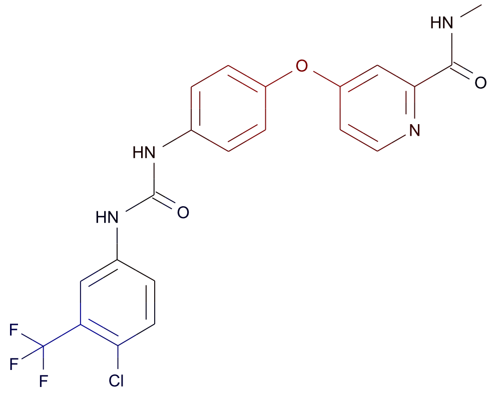

$C_{21}H_{16}ClF_3N_4O_3$
Molecular Weight: 464.82494
ALogP: 4.175
Rotatable Bonds: 6
Acceptors: 4
Donors: 3

Model Prediction

Prediction: Carcinogen
Probability: 0.257
Enrichment: 0.801
Bayesian Score: -0.321
Mahalanobis Distance: 14.9
Mahalanobis Distance p-value: 4.21e-007

Prediction: Positive if the Bayesian score is above the estimated best cutoff value from minimizing the false positive and false negative rate.
Probability: The esimated probability that the sample is in the positive category. This assumes that the Bayesian score follows a normal distribution and is different from the prediction using a cutoff.
Enrichment: An estimate of enrichment, that is, the increased likelihood (versus random) of this sample being in the category.
Bayesian Score: The standard Laplacian-modified Bayesian score.
Mahalanobis Distance: The Mahalanobis distance (MD) is the distance to the center of the training data. The larger the MD, the less trustworthy the prediction.
Mahalanobis Distance p-value: The p-value gives the fraction of training data with an MD greater than or equal to the one for the given sample, assuming normally distributed data. The smaller the p-value, the less trustworthy the prediciton. For highly non-normal X properties (e.g., fingerprints), the MD p-value is wildly inaccurate.

Structural Similar Compounds

Name	Glimepride	Glyburide	Fluvastatin
Structure	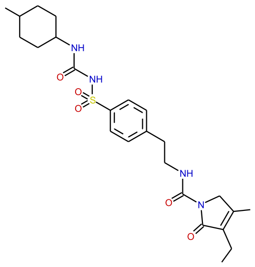	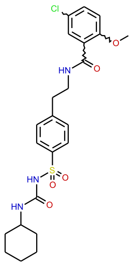	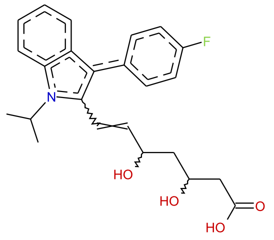
Actual Endpoint	Carcinogen	Non-Carcinogen	Non-Carcinogen
Predicted Endpoint	Carcinogen	Non-Carcinogen	Non-Carcinogen
Distance	0.605	0.615	0.625
Reference	US FDA (Centre for Drug Eval.& Res./Off. Testing & Res.) Sept. 1997	US FDA (Centre for Drug Eval.& Res./Off. Testing & Res.) Sept. 1997	US FDA (Centre for Drug Eval.& Res./Off. Testing & Res.) Sept. 1997

Model Applicability

Unknown features are fingerprint features in the query molecule, but not found or appearing too infrequently in the training set.

1. OPS PC20 out of range. Value: -3.3309. Training min, max, SD, explained variance: -3.1862, 4.4571, 1.28, 0.0167.

Feature Contribution

Top features for positive contribution

Fingerprint	Bit/Smiles	Feature Structure	Score	Carcinogen in training set
ECFP_6	738938915	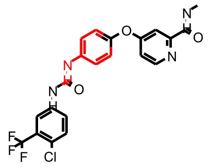 [*]C(=[*])N[c]1:[cH]:[cH]:[*]:[cH]:[cH]:1	0.617	2 out of 2

ECFP_6	1338334141	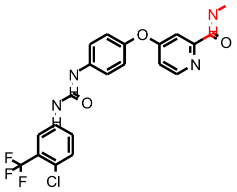 [*]C(=[*])NC	0.442	2 out of 3
ECFP_6	1305253718	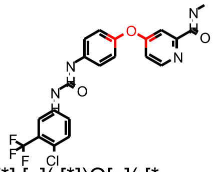 [*]:[c](:[*])O[c](:[*]):[*]	0.424	1 out of 1

Top Features for negative contribution

Fingerprint	Bit/Smiles	Feature Structure	Score	Carcinogen in training set
ECFP_6	1335691903	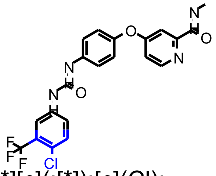 [*][c](:[*]):[c](Cl):[cH]:[*]	-0.669	3 out of 22
ECFP_6	1336678434	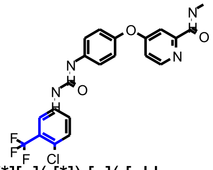 [*][c](:[*]):[c](:[cH]:[*])C([*])([*])[*]	-0.657	0 out of 3
ECFP_6	-1952889961	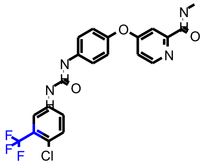 [*]:[c](:[*])C(F)(F)F	-0.657	0 out of 3

Sorafenib

TOPKAT_Mouse_Female_FDA_Single_vs_Multiple

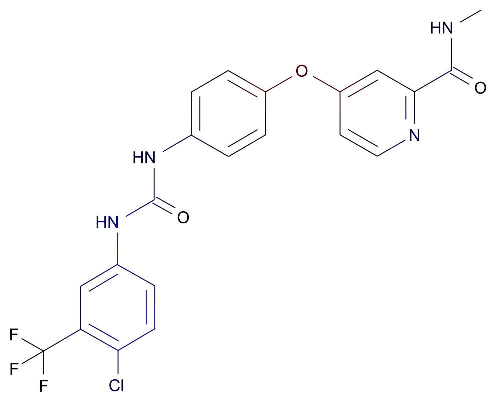

$C_{21}H_{16}ClF_3N_4O_3$
Molecular Weight: 464.82494
ALogP: 4.175
Rotatable Bonds: 6
Acceptors: 4
Donors: 3

Model Prediction

Prediction: Single-Carcinogen
Probability: 0.283
Enrichment: 0.691
Bayesian Score: -3.89
Mahalanobis Distance: 11.1
Mahalanobis Distance p-value: 0.00221

Prediction: Positive if the Bayesian score is above the estimated best cutoff value from minimizing the false positive and false negative rate.
Probability: The esimated probability that the sample is in the positive category. This assumes that the Bayesian score follows a normal distribution and is different from the prediction using a cutoff.
Enrichment: An estimate of enrichment, that is, the increased likelihood (versus random) of this sample being in the category.
Bayesian Score: The standard Laplacian-modified Bayesian score.
Mahalanobis Distance: The Mahalanobis distance (MD) is the distance to the center of the training data. The larger the MD, the less trustworthy the prediction.
Mahalanobis Distance p-value: The p-value gives the fraction of training data with an MD greater than or equal to the one for the given sample, assuming normally distributed data. The smaller the p-value, the less trustworthy the prediciton. For highly non-normal X properties (e.g., fingerprints), the MD p-value is wildly inaccurate.

Structural Similar Compounds

Name	Glimepride	Labetalol	Lansoprazole
Structure	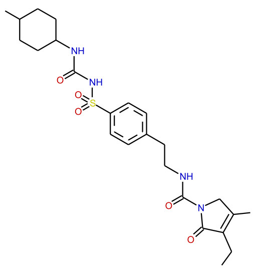	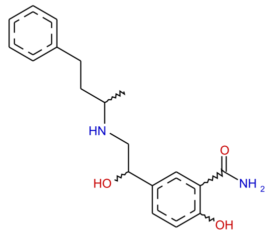	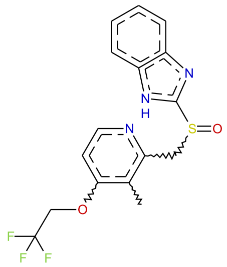
Actual Endpoint	Single-Carcinogen	Single-Carcinogen	Single-Carcinogen
Predicted Endpoint	Single-Carcinogen	Single-Carcinogen	Single-Carcinogen
Distance	0.599	0.808	0.820
Reference	US FDA (Centre for Drug Eval.& Res./Off. Testing & Res.) Sept. 1997	US FDA (Centre for Drug Eval.& Res./Off. Testing & Res.) Sept. 1997	US FDA (Centre for Drug Eval.& Res./Off. Testing & Res.) Sept. 1997

Model Applicability

Unknown features are fingerprint features in the query molecule, but not found or appearing too infrequently in the training set.

1. All properties and OPS components are within expected ranges.
2. Unknown ECFP_2 feature: 1336678434: [*][c](:[*]):[c](C([*])([*])[*]):c:[*]
3. Unknown ECFP_2 feature: -1952889961: [*]:[c](:[*])C(F)(F)F

Feature Contribution

Top features for positive contribution

Fingerprint	Bit/Smiles	Feature Structure	Score	Multiple-Carcinogen in training set
ECFP_4	-834094296	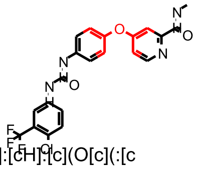 [*]:[cH]:[c](O[c](:[cH]:[*]):[cH]:[*]):[cH]:[*]	0.351	1 out of 1

ECFP_4	1407472008	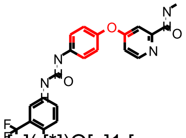 [*]:[c](:[*])O[c]1:[cH]:[cH]:[*]:[cH]:[cH]:1	0.351	1 out of 1
ECFP_4	143734695	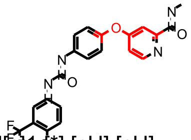 [*][c]1:[*]:[cH]:[cH]:[c](O[c](:[*]):[*]):[cH]:1	0.351	1 out of 1
**Top Features for negative contribution**				
**Fingerprint**	**Bit/Smiles**	**Feature Structure**	**Score**	**Multiple-Carcinogen in training set**
ECFP_4	888054369	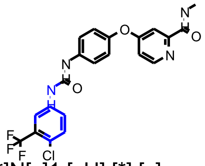 [*]N[c]1:[cH]:[*]:[c]([*]):[cH]:[cH]:1	-0.8	0 out of 3
ECFP_4	1335691903	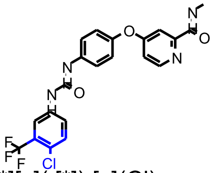 [*][c](:[*]):[c](Cl):[cH]:[*]	-0.8	0 out of 3
ECFP_4	1338334141	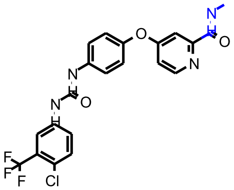 [*]C(=[*])NC	-0.597	0 out of 2

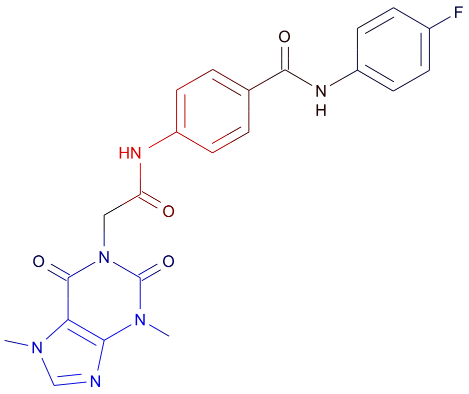

$C_{22}H_{19}FN_6O_4$
Molecular Weight: 450.42246
ALogP: 1.526
Rotatable Bonds: 5
Acceptors: 5
Donors: 2

Model Prediction

Prediction: Non-Carcinogen
Probability: 0.225
Enrichment: 0.765
Bayesian Score: -3.52
Mahalanobis Distance: 13.4
Mahalanobis Distance p-value: 5.92e-005

Prediction: Positive if the Bayesian score is above the estimated best cutoff value from minimizing the false positive and false negative rate.
Probability: The esimated probability that the sample is in the positive category. This assumes that the Bayesian score follows a normal distribution and is different from the prediction using a cutoff.
Enrichment: An estimate of enrichment, that is, the increased likelihood (versus random) of this sample being in the category.
Bayesian Score: The standard Laplacian-modified Bayesian score.
Mahalanobis Distance: The Mahalanobis distance (MD) is the distance to the center of the training data. The larger the MD, the less trustworthy the prediction.
Mahalanobis Distance p-value: The p-value gives the fraction of training data with an MD greater than or equal to the one for the given sample, assuming normally distributed data. The smaller the p-value, the less trustworthy the prediciton. For highly non-normal X properties (e.g., fingerprints), the MD p-value is wildly inaccurate.

Structural Similar Compounds

Name	Bicalutamide	Glipizide	Indapamide
Structure	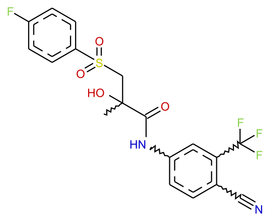	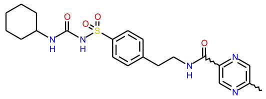	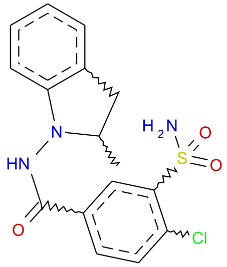
Actual Endpoint	Carcinogen	Non-Carcinogen	Non-Carcinogen
Predicted Endpoint	Carcinogen	Non-Carcinogen	Non-Carcinogen
Distance	0.548	0.666	0.674
Reference	US FDA (Centre for Drug Eval.& Res./Off. Testing & Res.) Sept. 1997	US FDA (Centre for Drug Eval.& Res./Off. Testing & Res.) Sept. 1997	US FDA (Centre for Drug Eval.& Res./Off. Testing & Res.) Sept. 1997

Model Applicability

Unknown features are fingerprint features in the query molecule, but not found or appearing too infrequently in the training set.

1. All properties and OPS components are within expected ranges.

Feature Contribution

Top features for positive contribution

Fingerprint	Bit/Smiles	Feature Structure	Score	Carcinogen in training set
FCFP_6	-581879738	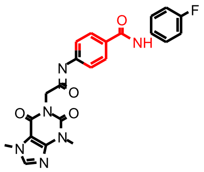 [*]NC(=O)[c]1:[cH]:[cH]:[*]:[cH]:[cH]:1	0.77	4 out of 5

FCFP_6	-451043714	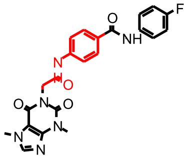 [*]CC(=O)N[c]1:[cH]:[cH]:[c]([*]):[cH]:[cH]:1	0.676	2 out of 2
FCFP_6	1175665944	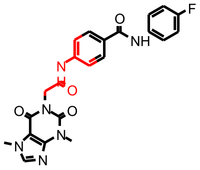 [*]CC(=O)N[c](:[cH]:[*]):[cH]:[*]	0.655	7 out of 12

Top Features for negative contribution

Fingerprint	Bit/Smiles	Feature Structure	Score	Carcinogen in training set
FCFP_6	-124685461	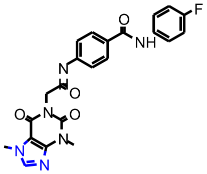 [*]n1:[*]:[*]:n:[cH]:1	-0.731	1 out of 12
FCFP_6	-1553874037	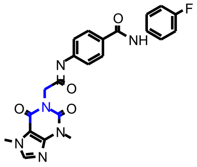 [*]CN(C(=[*])[*])C(=[*])[*]	-0.45	5 out of 32
FCFP_6	551850122	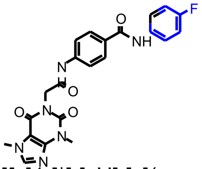 [*][c]1:[*]:[cH]:[c](F):[cH]:[cH]:1	-0.433	8 out of 49

Sorafenib

TOPKAT_Mouse_Male_FDA_None_vs_Carcinogen

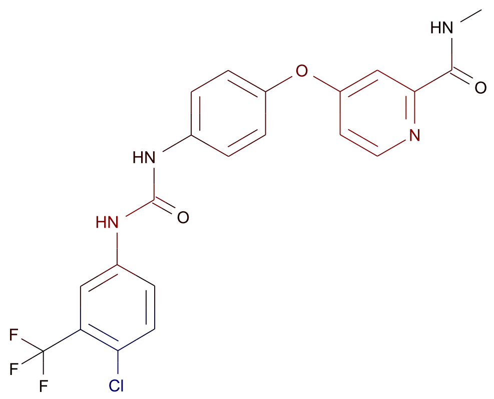

$C_{21}H_{16}ClF_3N_4O_3$

Molecular Weight: 464.82494

ALogP: 4.175

Rotatable Bonds: 6

Acceptors: 4

Donors: 3

Model Prediction

Prediction: Carcinogen

Probability: 0.444

Enrichment: 1.51

Bayesian Score: 4.21

Mahalanobis Distance: 20.3

Mahalanobis Distance p-value: 1.28e-019

Prediction: Positive if the Bayesian score is above the estimated best cutoff value from minimizing the false positive and false negative rate.
Probability: The esimated probability that the sample is in the positive category. This assumes that the Bayesian score follows a normal distribution and is different from the prediction using a cutoff.
Enrichment: An estimate of enrichment, that is, the increased likelihood (versus random) of this sample being in the category.
Bayesian Score: The standard Laplacian-modified Bayesian score.
Mahalanobis Distance: The Mahalanobis distance (MD) is the distance to the center of the training data. The larger the MD, the less trustworthy the prediction.
Mahalanobis Distance p-value: The p-value gives the fraction of training data with an MD greater than or equal to the one for the given sample, assuming normally distributed data. The smaller the p-value, the less trustworthy the prediciton. For highly non-normal X properties (e.g., fingerprints), the MD p-value is wildly inaccurate.

Structural Similar Compounds

Name	Glyburide	Glimepride	Fluvastatin
Structure	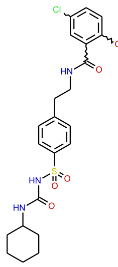	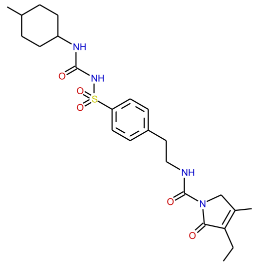	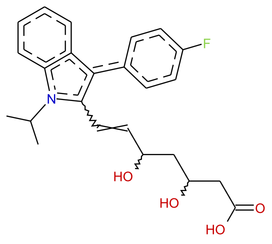
Actual Endpoint	Non-Carcinogen	Carcinogen	Non-Carcinogen
Predicted Endpoint	Non-Carcinogen	Carcinogen	Non-Carcinogen
Distance	0.594	0.599	0.603
Reference	US FDA (Centre for Drug Eval.& Res./Off. Testing & Res.) Sept. 1997	US FDA (Centre for Drug Eval.& Res./Off. Testing & Res.) Sept. 1997	US FDA (Centre for Drug Eval.& Res./Off. Testing & Res.) Sept. 1997

Model Applicability

Unknown features are fingerprint features in the query molecule, but not found or appearing too infrequently in the training set.

1. All properties and OPS components are within expected ranges.

Feature Contribution

Top features for positive contribution

Fingerprint	Bit/Smiles	Feature Structure	Score	Carcinogen in training set
FCFP_6	71953198	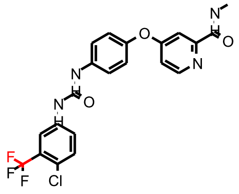 [*]C([*])([*])F	0.612	12 out of 23

FCFP_6	-1838187238	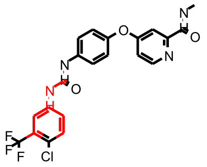 [*]C(=[*])N[c]1:[cH]:[cH]:[*]:[cH]:[cH]:1	0.565	4 out of 7
FCFP_6	-1270820019	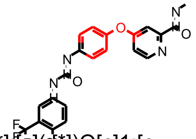 [*]:[c](:[*])O[c]1:[cH]:[cH]:[*]:[cH]:[cH]:1	0.46	1 out of 1

Top Features for negative contribution

Fingerprint	Bit/Smiles	Feature Structure	Score	Carcinogen in training set
FCFP_6	2104062943	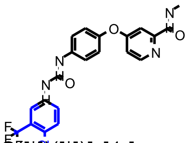 [*]C([*])([*])[c]1:[cH]:[*]:[cH]:[cH]:[c]:1Cl	-1.01	1 out of 17
FCFP_6	551850122	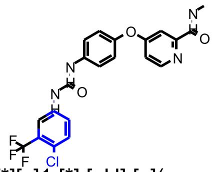 [*][c]1:[*]:[cH]:[c](F):[cH]:[cH]:1	-0.433	8 out of 49
FCFP_6	71476542	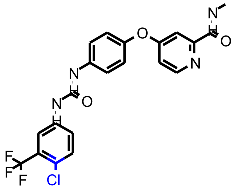 [*]:[c](:[*])F	-0.406	10 out of 59

Sorafenib

TOPKAT_Mouse_Male_FDA_Single_vs_Multiple

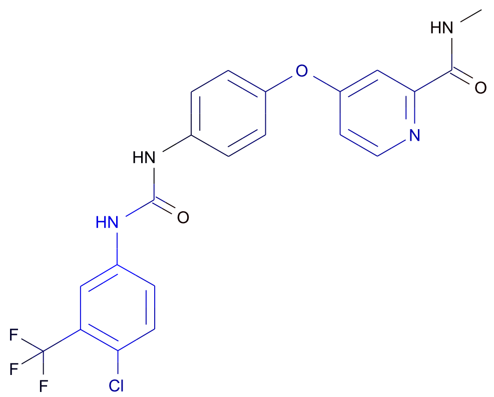

$C_{21}H_{16}ClF_3N_4O_3$
Molecular Weight: 464.82494
ALogP: 4.175
Rotatable Bonds: 6
Acceptors: 4
Donors: 3

Model Prediction

Prediction: Single-Carcinogen
Probability: 0.139
Enrichment: 0.461
Bayesian Score: -14.7
Mahalanobis Distance: 21.3
Mahalanobis Distance p-value: 4.93e-011

Prediction: Positive if the Bayesian score is above the estimated best cutoff value from minimizing the false positive and false negative rate.
Probability: The esimated probability that the sample is in the positive category. This assumes that the Bayesian score follows a normal distribution and is different from the prediction using a cutoff.
Enrichment: An estimate of enrichment, that is, the increased likelihood (versus random) of this sample being in the category.
Bayesian Score: The standard Laplacian-modified Bayesian score.
Mahalanobis Distance: The Mahalanobis distance (MD) is the distance to the center of the training data. The larger the MD, the less trustworthy the prediction.
Mahalanobis Distance p-value: The p-value gives the fraction of training data with an MD greater than or equal to the one for the given sample, assuming normally distributed data. The smaller the p-value, the less trustworthy the prediciton. For highly non-normal X properties (e.g., fingerprints), the MD p-value is wildly inaccurate.

Structural Similar Compounds

Name	Glimepride	Bicalutamide	Lansoprazole
Structure	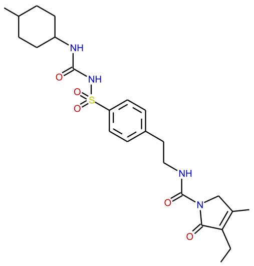	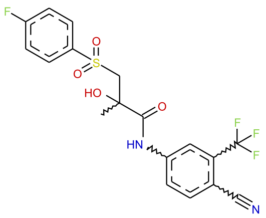	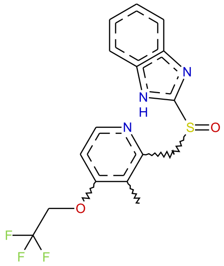
Actual Endpoint	Single-Carcinogen	Single-Carcinogen	Single-Carcinogen
Predicted Endpoint	Single-Carcinogen	Single-Carcinogen	Single-Carcinogen
Distance	0.626	0.700	0.866
Reference	US FDA (Centre for Drug Eval.& Res./Off. Testing & Res.) Sept. 1997	US FDA (Centre for Drug Eval.& Res./Off. Testing & Res.) Sept. 1997	US FDA (Centre for Drug Eval.& Res./Off. Testing & Res.) Sept. 1997

Model Applicability

Unknown features are fingerprint features in the query molecule, but not found or appearing too infrequently in the training set.

1. All properties and OPS components are within expected ranges.

Feature Contribution

Top features for positive contribution

Fingerprint	Bit/Smiles	Feature Structure	Score	Multiple-Carcinogen in training set
FCFP_12	1499521844	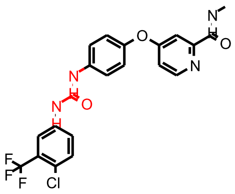 [*]NC(=O)N[*]	0.39	5 out of 9

FCFP_12	-904785030	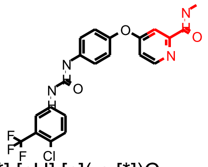 [*]:[cH]:[c](:n:[*])C(=O)NC	0.174	1 out of 2
FCFP_12	-1549103449	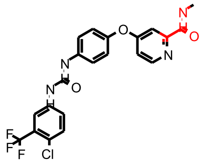 [*]NC(=O)[c](:[*]):[*]	0.168	3 out of 7

Top Features for negative contribution

Fingerprint	Bit/Smiles	Feature Structure	Score	Multiple-Carcinogen in training set
FCFP_12	1294255210	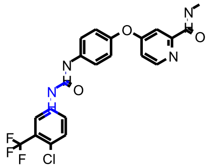 [*]C(=[*])N[c](:[*]):[*]	-1.63	0 out of 12
FCFP_12	590925877	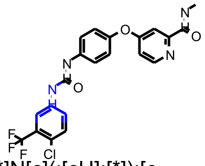 [*]N[c](:[cH]:[*]):[cH]:[*]	-0.998	1 out of 13
FCFP_12	-1462709112	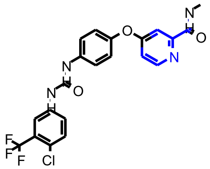 [*]C(=[*])[c]1:[cH]:[*]:[cH]:[cH]:n:1	-0.994	0 out of 5

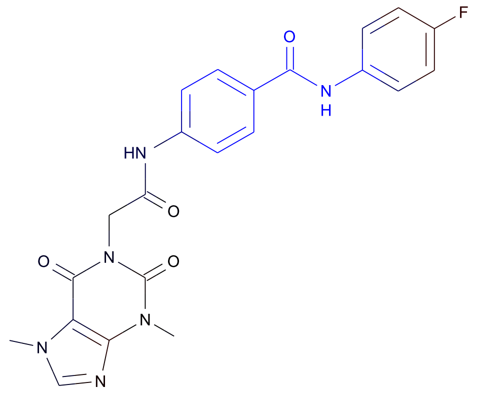

$C_{22}H_{19}FN_6O_4$
Molecular Weight: 450.42246
ALogP: 1.526
Rotatable Bonds: 5
Acceptors: 5
Donors: 2

Model Prediction

Prediction: Mild
Probability: 0.238
Enrichment: 0.345
Bayesian Score: -8.87
Mahalanobis Distance: 10.6
Mahalanobis Distance p-value: 0.0161

Prediction: Positive if the Bayesian score is above the estimated best cutoff value from minimizing the false positive and false negative rate.
Probability: The esimated probability that the sample is in the positive category. This assumes that the Bayesian score follows a normal distribution and is different from the prediction using a cutoff.
Enrichment: An estimate of enrichment, that is, the increased likelihood (versus random) of this sample being in the category.
Bayesian Score: The standard Laplacian-modified Bayesian score.
Mahalanobis Distance: The Mahalanobis distance (MD) is the distance to the center of the training data. The larger the MD, the less trustworthy the prediction.
Mahalanobis Distance p-value: The p-value gives the fraction of training data with an MD greater than or equal to the one for the given sample, assuming normally distributed data. The smaller the p-value, the less trustworthy the prediciton. For highly non-normal X properties (e.g., fingerprints), the MD p-value is wildly inaccurate.

Structural Similar Compounds

Name	5-NORBORNENE-2;3-DICARBOXYLIC ACID; 1;4;5;6;7;7-HEXACHLORO-	1-AMINO-4-BENZOYLAMINO-ANTHRAQUINONE	ANTHRAQUINONE; 1-AMINO-4-HYDROXY-2-PHENOXY-
Structure	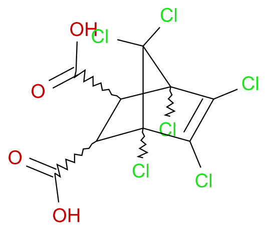	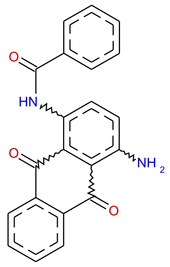	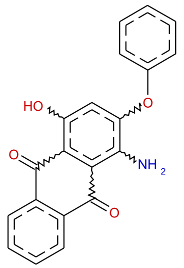
Actual Endpoint	Moderate_Severe	Mild	Mild
Predicted Endpoint	Moderate_Severe	Mild	Mild
Distance	0.676	0.701	0.766
Reference	28ZPAK-;92;72	28ZPAK-;124;72	28ZPAK 239;72

Model Applicability

Unknown features are fingerprint features in the query molecule, but not found or appearing too infrequently in the training set.

1. All properties and OPS components are within expected ranges.
2. Unknown FCFP_2 feature: -124685461: [*]n1:[*]:[*]:n:[cH]:1
3. Unknown FCFP_2 feature: 136150461: [*]:n(:[*])C

Feature Contribution

Top features for positive contribution

Fingerprint	Bit/Smiles	Feature Structure	Score	Moderate_Severe in training set
FCFP_10	-1508180856	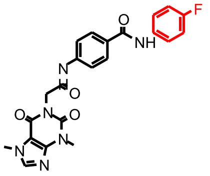 [*][c]1:[cH]:[cH]:[c](F):[cH]:[cH]:1	0.329	16 out of 17

FCFP_10	-745491832	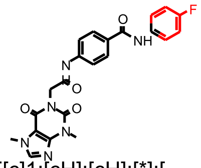 F[c]1:[cH]:[cH]:[*]:[cH]:[cH]:1	0.304	29 out of 32
FCFP_10	-1410049896	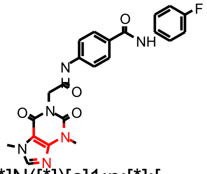 [*]N([*])[c]1:n:[*]:[*]:[c]:1[*]	0.256	2 out of 2
**Top Features for negative contribution**				
**Fingerprint**	**Bit/Smiles**	**Feature Structure**	**Score**	**Moderate_Severe in training set**
FCFP_10	1175232969	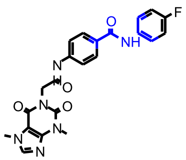 [*]:[cH]:[c](NC(=O)[c](:[*]):[*]):[cH]:[*]	-1.29	0 out of 4
FCFP_10	-581879738	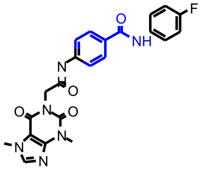 [*]NC(=O)[c]1:[cH]:[c H]:[*]:[cH]:[cH]:1	-1.29	0 out of 4
FCFP_10	-1925475824	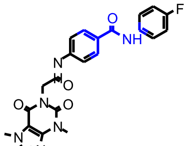 [*]:[cH]:[c](:[cH]:[*])C(=O)N[c](:[*]):[*]	-1.29	0 out of 4

Sorafenib

TOPKAT_Ocular_Irritancy_Mild_vs_Moderate_Severe

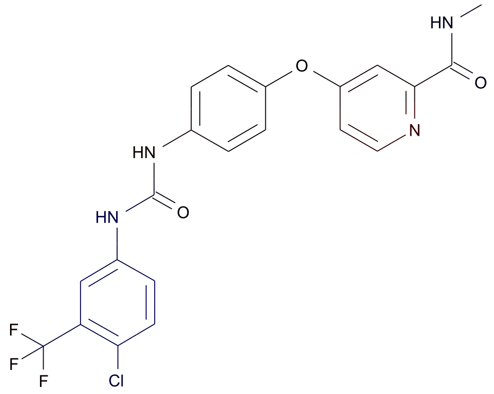

$C_{21}H_{16}ClF_3N_4O_3$

Molecular Weight: 464.82494

ALogP: 4.175

Rotatable Bonds: 6

Acceptors: 4

Donors: 3

Model Prediction

Prediction: Mild

Probability: 0.776

Enrichment: 1.13

Bayesian Score: -1.8

Mahalanobis Distance: 8.95

Mahalanobis Distance p-value: 0.537

Prediction: Positive if the Bayesian score is above the estimated best cutoff value from minimizing the false positive and false negative rate.
Probability: The esimated probability that the sample is in the positive category. This assumes that the Bayesian score follows a normal distribution and is different from the prediction using a cutoff.
Enrichment: An estimate of enrichment, that is, the increased likelihood (versus random) of this sample being in the category.
Bayesian Score: The standard Laplacian-modified Bayesian score.
Mahalanobis Distance: The Mahalanobis distance (MD) is the distance to the center of the training data. The larger the MD, the less trustworthy the prediction.
Mahalanobis Distance p-value: The p-value gives the fraction of training data with an MD greater than or equal to the one for the given sample, assuming normally distributed data. The smaller the p-value, the less trustworthy the prediciton. For highly non-normal X properties (e.g., fingerprints), the MD p-value is wildly inaccurate.

Structural Similar Compounds

Name	4;4'-DIAMINO-1;1'-DIANTHRIMIDE	5-NORBORNENE-2;3-DICARBOXYLIC ACID; 1;4;5;6;7;7-HEXACHLORO-	METHANE;TRIS(4-AMINOPHENYL)-
Structure	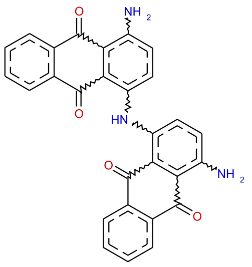	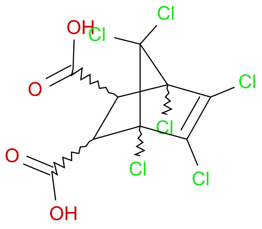	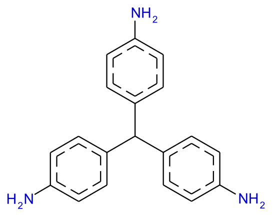
Actual Endpoint	Mild	Moderate_Severe	Moderate_Severe
Predicted Endpoint	Mild	Moderate_Severe	Moderate_Severe
Distance	0.799	0.816	0.827
Reference	28ZPAK-;125;72	28ZPAK-;92;72	28ZPAK-;73;72

Model Applicability

Unknown features are fingerprint features in the query molecule, but not found or appearing too infrequently in the training set.

1. All properties and OPS components are within expected ranges.

Feature Contribution

Top features for positive contribution				
**Fingerprint**	**Bit/Smiles**	**Feature Structure**	**Score**	**Moderate_Severe in training set**
FCFP_10	-1695756380	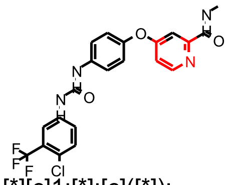 [*][c]1:[*]:[c]([*]):n:[cH]:[cH]:1	0.285	10 out of 11

FCFP_10	-124655670	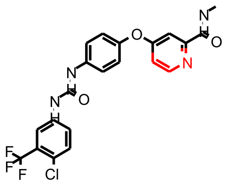 [*]:[cH]:[cH]:n:[*]	0.259	14 out of 16
FCFP_10	-885550502	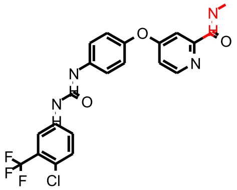 [*]C(=[*])NC	0.239	54 out of 64
**Top Features for negative contribution**				
**Fingerprint**	**Bit/Smiles**	**Feature Structure**	**Score**	**Moderate_Severe in training set**
FCFP_10	2104062943	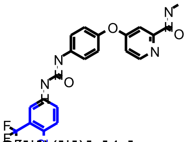 [*]C([*])([*])[c]1:[cH]:[*]:[cH]:[cH]:[c]:1Cl	-0.745	7 out of 24
FCFP_10	-174293376	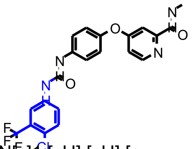 [*]N[c]1:[cH]:[cH]:[c](Cl):[c](:[cH]:1)C([*])([*])[*]	-0.507	0 out of 1
FCFP_10	-1549103449	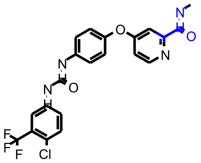 [*]NC(=O)[c](:[*]):[*]	-0.504	2 out of 6

27RR

TOPKAT_Ocular_Irritancy_None_vs_Irritant

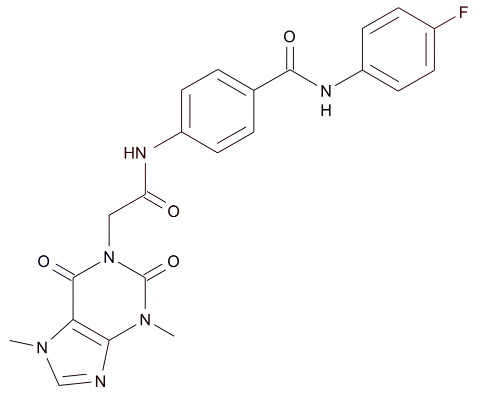

$C_{22}H_{19}FN_6O_4$
Molecular Weight: 450.42246
ALogP: 1.526
Rotatable Bonds: 5
Acceptors: 5
Donors: 2

Model Prediction

Prediction: Irritant
Probability: 1
Enrichment: 1.18
Bayesian Score: 2.71
Mahalanobis Distance: 8.83
Mahalanobis Distance p-value: 0.606

Prediction: Positive if the Bayesian score is above the estimated best cutoff value from minimizing the false positive and false negative rate.
Probability: The esimated probability that the sample is in the positive category. This assumes that the Bayesian score follows a normal distribution and is different from the prediction using a cutoff.
Enrichment: An estimate of enrichment, that is, the increased likelihood (versus random) of this sample being in the category.
Bayesian Score: The standard Laplacian-modified Bayesian score.
Mahalanobis Distance: The Mahalanobis distance (MD) is the distance to the center of the training data. The larger the MD, the less trustworthy the prediction.
Mahalanobis Distance p-value: The p-value gives the fraction of training data with an MD greater than or equal to the one for the given sample, assuming normally distributed data. The smaller the p-value, the less trustworthy the prediciton. For highly non-normal X properties (e.g., fingerprints), the MD p-value is wildly inaccurate.

Structural Similar Compounds

Name	5-NORBORNENE-2;3-DICARBOXYLIC ACID; 1;4;5;6;7;7-HEXACHLORO-	1-AMINO-4-BENZOYLAMINO-ANTHRAQUINONE	ANTHRAQUINONE; 1-AMINO-4-HYDROXY-2-PHENOXY-
Structure	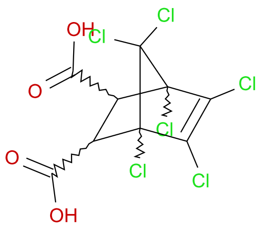	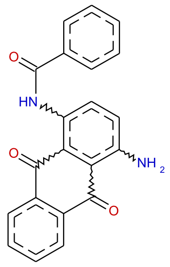	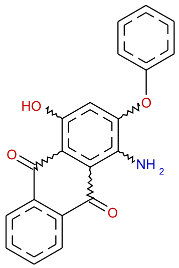
Actual Endpoint	Irritant	Irritant	Irritant
Predicted Endpoint	Irritant	Irritant	Irritant
Distance	0.662	0.688	0.743
Reference	28ZPAK-;92;72	28ZPAK-;124;72	28ZPAK 239;72

Model Applicability

Unknown features are fingerprint features in the query molecule, but not found or appearing too infrequently in the training set.

1. All properties and OPS components are within expected ranges.
2. Unknown FCFP_2 feature: -124685461: [*]n1:[*]:[*]:n:[cH]:1
3. Unknown FCFP_2 feature: 136150461: [*]:n(:[*])C

Feature Contribution

Top features for positive contribution				
**Fingerprint**	**Bit/Smiles**	**Feature Structure**	**Score**	**Irritant in training set**
FCFP_12	1747237384	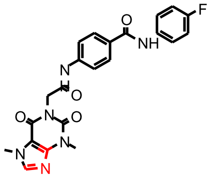 [*][c]1:[*]:[*]:[cH]:n:1	0.208	44 out of 44

FCFP_12	-1508180856	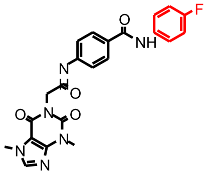<br>[*][c]1:[cH]:[cH]:[c](F):[cH]:[cH]:1	0.2	17 out of 17
FCFP_12	1175665944	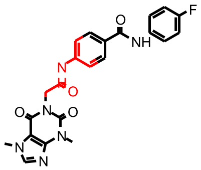<br>[*]CC(=O)N[c](:[cH]:[*]):[cH]:[*]	0.198	14 out of 14

Top Features for negative contribution

Fingerprint	Bit/Smiles	Feature Structure	Score	Irritant in training set
FCFP_12	-1549163031	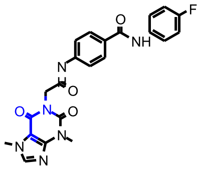<br>[*]N([*])C(=O)[c](:[*]):[*]	-0.623	16 out of 38
FCFP_12	0	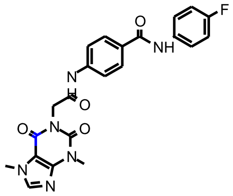<br>[*]C(=[*])[*]	0	1184 out of 1397
FCFP_12	1872154524	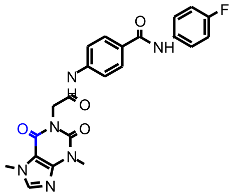<br>[*]C(=O)[*]	0	563 out of 690

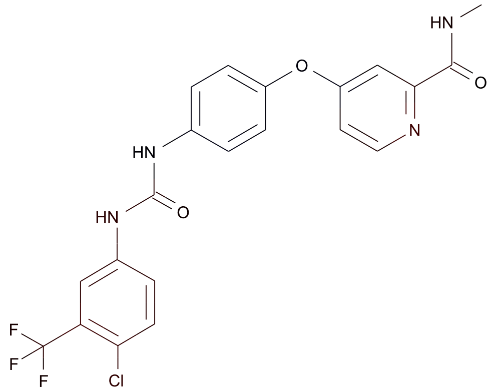

$C_{21}H_{16}ClF_3N_4O_3$
Molecular Weight: 464.82494
ALogP: 4.175
Rotatable Bonds: 6
Acceptors: 4
Donors: 3

Model Prediction

Prediction: Irritant
Probability: 1
Enrichment: 1.18
Bayesian Score: 3.04
Mahalanobis Distance: 6.28
Mahalanobis Distance p-value: 1

Prediction: Positive if the Bayesian score is above the estimated best cutoff value from minimizing the false positive and false negative rate.
Probability: The esimated probability that the sample is in the positive category. This assumes that the Bayesian score follows a normal distribution and is different from the prediction using a cutoff.
Enrichment: An estimate of enrichment, that is, the increased likelihood (versus random) of this sample being in the category.
Bayesian Score: The standard Laplacian-modified Bayesian score.
Mahalanobis Distance: The Mahalanobis distance (MD) is the distance to the center of the training data. The larger the MD, the less trustworthy the prediction.
Mahalanobis Distance p-value: The p-value gives the fraction of training data with an MD greater than or equal to the one for the given sample, assuming normally distributed data. The smaller the p-value, the less trustworthy the prediciton. For highly non-normal X properties (e.g., fingerprints), the MD p-value is wildly inaccurate.

Structural Similar Compounds

Name	BENZANILIDE;2';2'''-DITHIOBIS-	4;4'-DIAMINO-1;1'-DIANTHRIMIDE	5-NORBORNENE-2;3-DICARBOXYLIC ACID; 1;4;5;6;7;7-HEXACHLORO-
Structure	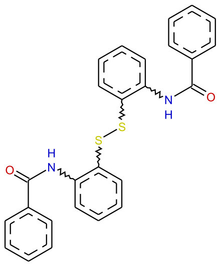	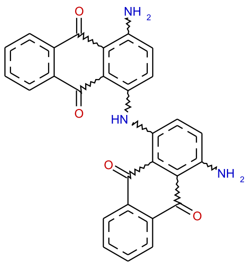	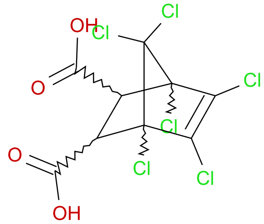
Actual Endpoint	Non-Irritant	Irritant	Irritant
Predicted Endpoint	Non-Irritant	Irritant	Irritant
Distance	0.743	0.791	0.801
Reference	28ZPAK-;173;72	28ZPAK-;125;72	28ZPAK-;92;72

Model Applicability

Unknown features are fingerprint features in the query molecule, but not found or appearing too infrequently in the training set.

1. All properties and OPS components are within expected ranges.

Feature Contribution

Top features for positive contribution				
**Fingerprint**	**Bit/Smiles**	**Feature Structure**	**Score**	**Irritant in training set**
FCFP_12	1747237384	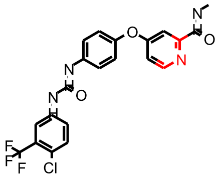 [*][c]1:[*]:[*]:[cH]:n:1	0.208	44 out of 44

FCFP_12	-124655670	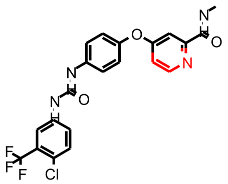<br>[*]:[cH]:[cH]:n:[*]	0.2	16 out of 16
FCFP_12	-1539132615	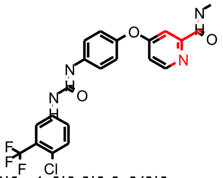<br>[*]n1:[*]:[*]:[c]([*]):[c]:1C(=[*])[*]	0.197	13 out of 13

Top Features for negative contribution

Fingerprint	Bit/Smiles	Feature Structure	Score	Irritant in training set
FCFP_12	-747629521	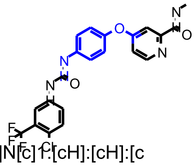<br>[*]N[c]1:[cH]:[cH]:[c](O[c](:[*]):[*]):[cH]:[cH]:1	-0.268	1 out of 2
FCFP_12	702861189	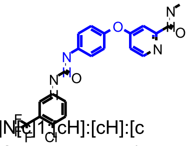<br>[*]N[c]1:[cH]:[cH]:[c](O[c]2:[cH]:[cH]:[*]:[c]([*]):[cH]:2):[cH]:[cH]:1	-0.268	1 out of 2
FCFP_12	-215363676	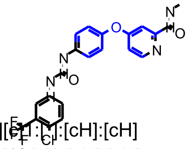<br>[*][c]1:[*]:[cH]:[cH]:[c](O[c]2:[cH]:[cH]:[*]:[cH]:[cH]:2):[cH]:1	0	4 out of 5

27RR

TOPKAT_Rat_Female_FDA_None_vs_Carcinogen

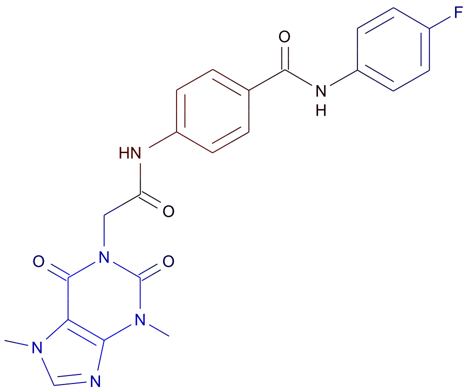

$C_{22}H_{19}FN_6O_4$

Molecular Weight: 450.42246

ALogP: 1.526

Rotatable Bonds: 5

Acceptors: 5

Donors: 2

Model Prediction

Prediction: Non-Carcinogen

Probability: 0.216

Enrichment: 0.672

Bayesian Score: -5.44

Mahalanobis Distance: 10.9

Mahalanobis Distance p-value: 0.083

Prediction: Positive if the Bayesian score is above the estimated best cutoff value from minimizing the false positive and false negative rate.

Probability: The esimated probability that the sample is in the positive category. This assumes that the Bayesian score follows a normal distribution and is different from the prediction using a cutoff.

Enrichment: An estimate of enrichment, that is, the increased likelihood (versus random) of this sample being in the category.

Bayesian Score: The standard Laplacian-modified Bayesian score.

Mahalanobis Distance: The Mahalanobis distance (MD) is the distance to the center of the training data. The larger the MD, the less trustworthy the prediction.

Mahalanobis Distance p-value: The p-value gives the fraction of training data with an MD greater than or equal to the one for the given sample, assuming normally distributed data. The smaller the p-value, the less trustworthy the prediciton. For highly non-normal X properties (e.g., fingerprints), the MD p-value is wildly inaccurate.

Structural Similar Compounds

Name	Bicalutamide	Polythiazide	Glipizide
Structure	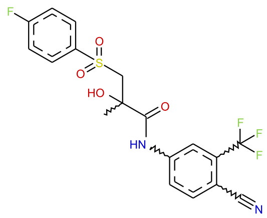	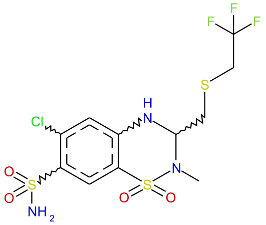	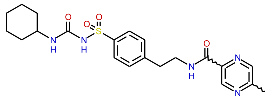
Actual Endpoint	Carcinogen	Non-Carcinogen	Non-Carcinogen
Predicted Endpoint	Carcinogen	Non-Carcinogen	Non-Carcinogen
Distance	0.605	0.681	0.692
Reference	US FDA (Centre for Drug Eval.& Res./Off. Testing & Res.) Sept. 1997	US FDA (Centre for Drug Eval.& Res./Off. Testing & Res.) Sept. 1997	US FDA (Centre for Drug Eval.& Res./Off. Testing & Res.) Sept. 1997

Model Applicability

Unknown features are fingerprint features in the query molecule, but not found or appearing too infrequently in the training set.

1. All properties and OPS components are within expected ranges.

Feature Contribution

Top features for positive contribution

Fingerprint	Bit/Smiles	Feature Structure	Score	Carcinogen in training set
ECFP_12	-223149939	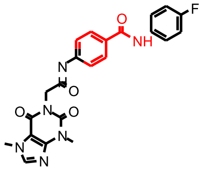 [*]NC(=O)[c]1:[cH]:[cH]:[*]:[cH]:[cH]:1	0.613	2 out of 2

ECFP_12	-177077903	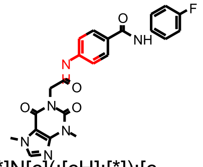 [*]N[c](:[cH]:[*]):[cH]:[*]	0.529	6 out of 10
ECFP_12	-1236483485	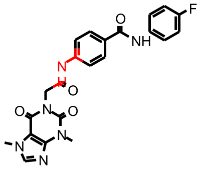 [*]C(=[*])N[c](:[*]):[*]	0.46	9 out of 17
**Top Features for negative contribution**				
**Fingerprint**	**Bit/Smiles**	**Feature Structure**	**Score**	**Carcinogen in training set**
ECFP_12	-1659169698	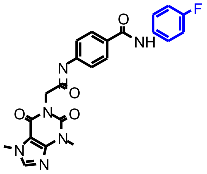 [*][c]1:[cH]:[cH]:[c](F):[cH]:[cH]:1	-0.56	1 out of 8
ECFP_12	-296909061	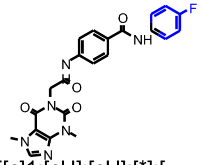 F[c]1:[cH]:[cH]:[*]:[cH]:[cH]:1	-0.56	1 out of 8
ECFP_12	-813242890	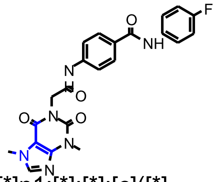 [*]n1:[*]:[*]:[c]([*]):[c]:1C(=[*])[*]	-0.485	0 out of 2

Sorafenib

TOPKAT_Rat_Female_FDA_None_vs_Carcinogen

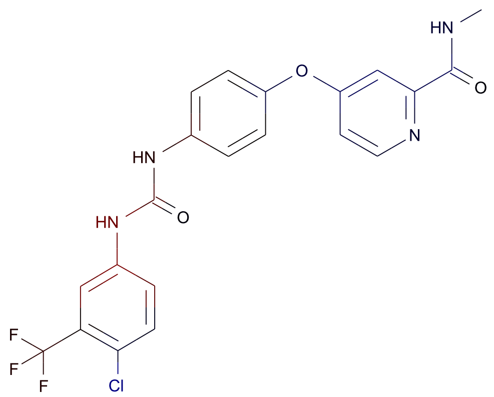

$C_{21}H_{16}ClF_3N_4O_3$
Molecular Weight: 464.82494
ALogP: 4.175
Rotatable Bonds: 6
Acceptors: 4
Donors: 3

Model Prediction

Prediction: Non-Carcinogen
Probability: 0.236
Enrichment: 0.734
Bayesian Score: -3.76
Mahalanobis Distance: 12.2
Mahalanobis Distance p-value: 0.00229

Prediction: Positive if the Bayesian score is above the estimated best cutoff value from minimizing the false positive and false negative rate.
Probability: The esimated probability that the sample is in the positive category. This assumes that the Bayesian score follows a normal distribution and is different from the prediction using a cutoff.
Enrichment: An estimate of enrichment, that is, the increased likelihood (versus random) of this sample being in the category.
Bayesian Score: The standard Laplacian-modified Bayesian score.
Mahalanobis Distance: The Mahalanobis distance (MD) is the distance to the center of the training data. The larger the MD, the less trustworthy the prediction.
Mahalanobis Distance p-value: The p-value gives the fraction of training data with an MD greater than or equal to the one for the given sample, assuming normally distributed data. The smaller the p-value, the less trustworthy the prediciton. For highly non-normal X properties (e.g., fingerprints), the MD p-value is wildly inaccurate.

Structural Similar Compounds

Name	Glimepride	Glyburide	Fluvastatin
Structure	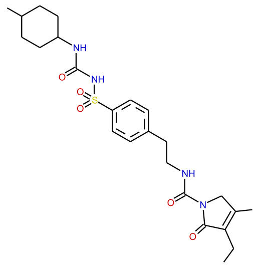	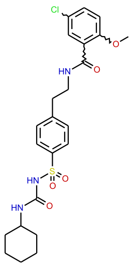	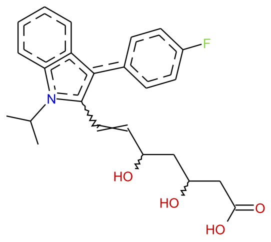
Actual Endpoint	Non-Carcinogen	Non-Carcinogen	Non-Carcinogen
Predicted Endpoint	Non-Carcinogen	Non-Carcinogen	Non-Carcinogen
Distance	0.620	0.635	0.635
Reference	US FDA (Centre for Drug Eval.& Res./Off. Testing & Res.) Sept. 1997	US FDA (Centre for Drug Eval.& Res./Off. Testing & Res.) Sept. 1997	US FDA (Centre for Drug Eval.& Res./Off. Testing & Res.) Sept. 1997

Model Applicability

Unknown features are fingerprint features in the query molecule, but not found or appearing too infrequently in the training set.

1. All properties and OPS components are within expected ranges.

Feature Contribution

Top features for positive contribution

Fingerprint	Bit/Smiles	Feature Structure	Score	Carcinogen in training set
ECFP_12	-970385855	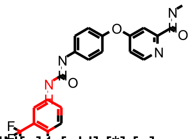 [*]N[c]1:[cH]:[*]:[c]([*]):[c](:[cH]:1)C([*])([*])[*]	0.613	2 out of 2

ECFP_12	-177077903	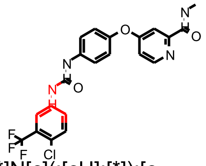 [*]N[c](:[cH]:[*]):[cH]:[*]	0.529	6 out of 10
ECFP_12	-1236483485	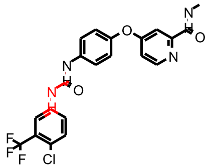 [*]C(=[*])N[c](:[*]):[*]	0.46	9 out of 17

Top Features for negative contribution

Fingerprint	Bit/Smiles	Feature Structure	Score	Carcinogen in training set
ECFP_12	1335691903	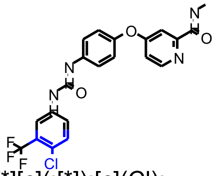 [*][c](:[*]):[c](Cl):[cH]:[*]	-1.11	2 out of 26
ECFP_12	99947387	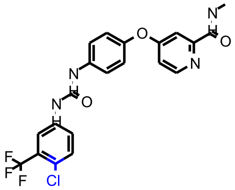 [*]:[c](:[*])Cl	-0.817	8 out of 62
ECFP_12	1413420509	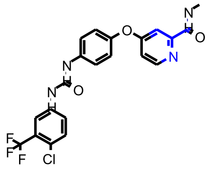 [*]C(=[*])[c](:[cH]:[*]):n:[*]	-0.661	0 out of 3

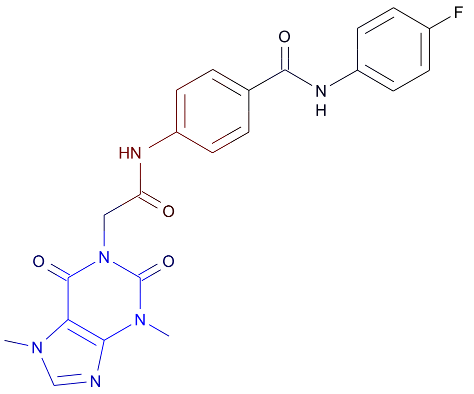

$C_{22}H_{19}FN_6O_4$
Molecular Weight: 450.42246
ALogP: 1.526
Rotatable Bonds: 5
Acceptors: 5
Donors: 2

Structural Similar Compounds

Name	Bicalutamide	Glipizide	Polythiazide
Structure	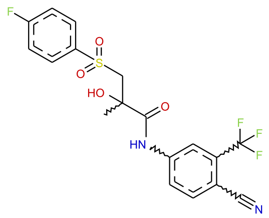	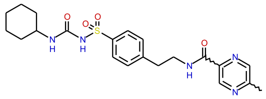	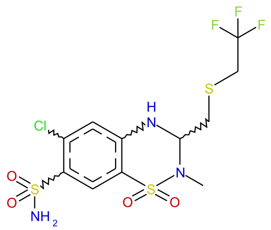
Actual Endpoint	Carcinogen	Non-Carcinogen	Non-Carcinogen
Predicted Endpoint	Carcinogen	Non-Carcinogen	Non-Carcinogen
Distance	0.567	0.669	0.671
Reference	US FDA (Centre for Drug Eval.& Res./Off. Testing & Res.) Sept. 1997	US FDA (Centre for Drug Eval.& Res./Off. Testing & Res.) Sept. 1997	US FDA (Centre for Drug Eval.& Res./Off. Testing & Res.) Sept. 1997

Model Prediction

Prediction: Non-Carcinogen
Probability: 0.236
Enrichment: 0.706
Bayesian Score: -5.13
Mahalanobis Distance: 14.6
Mahalanobis Distance p-value: 3.22e-006

Prediction: Positive if the Bayesian score is above the estimated best cutoff value from minimizing the false positive and false negative rate.
Probability: The esimated probability that the sample is in the positive category. This assumes that the Bayesian score follows a normal distribution and is different from the prediction using a cutoff.
Enrichment: An estimate of enrichment, that is, the increased likelihood (versus random) of this sample being in the category.
Bayesian Score: The standard Laplacian-modified Bayesian score.
Mahalanobis Distance: The Mahalanobis distance (MD) is the distance to the center of the training data. The larger the MD, the less trustworthy the prediction.
Mahalanobis Distance p-value: The p-value gives the fraction of training data with an MD greater than or equal to the one for the given sample, assuming normally distributed data. The smaller the p-value, the less trustworthy the prediciton. For highly non-normal X properties (e.g., fingerprints), the MD p-value is wildly inaccurate.

Model Applicability

Unknown features are fingerprint features in the query molecule, but not found or appearing too infrequently in the training set.

1. All properties and OPS components are within expected ranges.

Feature Contribution

Top features for positive contribution

Fingerprint	Bit/Smiles	Feature Structure	Score	Carcinogen in training set
SCFP_6	-347048986	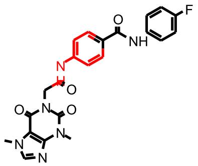 [*]C(=[*])N[c]1:[cH]:[cH]:[*]:[cH]:[cH]:1	0.615	5 out of 7

SCFP_6	814408713	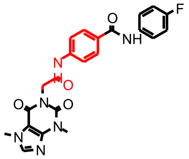 [*]CC(=O)N[c]1:[cH]:[cH]:[c]([*]):[cH]:[cH]:1	0.603	2 out of 2
SCFP_6	2097618059	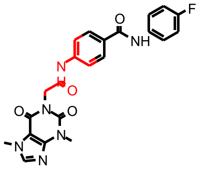 [*]CC(=O)N[c](:[cH]:[*]):[cH]:[*]	0.437	7 out of 13

Top Features for negative contribution

Fingerprint	Bit/Smiles	Feature Structure	Score	Carcinogen in training set
SCFP_6	399659969	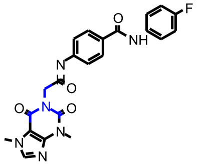 [*]CN(C(=[*])[*])C(=[*])[*]	-0.578	1 out of 8
SCFP_6	2144809592	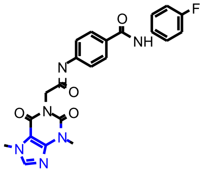 [*]N([*])[c]1:n:[cH]:n([*]):[c]:1[*]	-0.496	0 out of 2
SCFP_6	-2121589288	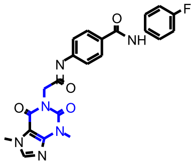 [*]CN1C(=[*])[*]:[c](:[*])N(C)C1=O	-0.496	0 out of 2

Sorafenib

TOPKAT_Rat_Male_FDA_None_vs_Carcinogen

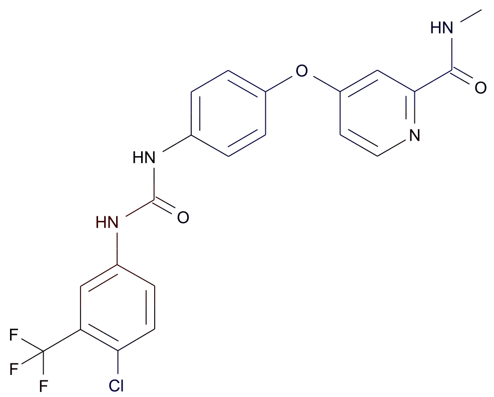

$C_{21}H_{16}ClF_3N_4O_3$

Molecular Weight: 464.82494

ALogP: 4.175

Rotatable Bonds: 6

Acceptors: 4

Donors: 3

Structural Similar Compounds

Name	Glyburide	Glimepride	Fluvastatin
Structure	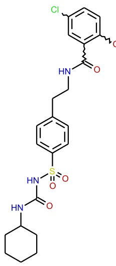	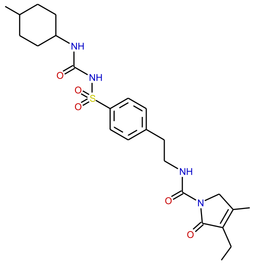	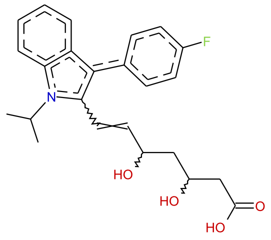
Actual Endpoint	Non-Carcinogen	Non-Carcinogen	Carcinogen
Predicted Endpoint	Non-Carcinogen	Non-Carcinogen	Carcinogen
Distance	0.593	0.600	0.615
Reference	US FDA (Centre for Drug Eval.& Res./Off. Testing & Res.) Sept. 1997	US FDA (Centre for Drug Eval.& Res./Off. Testing & Res.) Sept. 1997	US FDA (Centre for Drug Eval.& Res./Off. Testing & Res.) Sept. 1997

Model Prediction

Prediction: Non-Carcinogen

Probability: 0.293

Enrichment: 0.878

Bayesian Score: -2.4

Mahalanobis Distance: 17.6

Mahalanobis Distance p-value: 1.1e-012

Prediction: Positive if the Bayesian score is above the estimated best cutoff value from minimizing the false positive and false negative rate.
Probability: The esimated probability that the sample is in the positive category. This assumes that the Bayesian score follows a normal distribution and is different from the prediction using a cutoff.
Enrichment: An estimate of enrichment, that is, the increased likelihood (versus random) of this sample being in the category.
Bayesian Score: The standard Laplacian-modified Bayesian score.
Mahalanobis Distance: The Mahalanobis distance (MD) is the distance to the center of the training data. The larger the MD, the less trustworthy the prediction.
Mahalanobis Distance p-value: The p-value gives the fraction of training data with an MD greater than or equal to the one for the given sample, assuming normally distributed data. The smaller the p-value, the less trustworthy the prediciton. For highly non-normal X properties (e.g., fingerprints), the MD p-value is wildly inaccurate.

Model Applicability

Unknown features are fingerprint features in the query molecule, but not found or appearing too infrequently in the training set.

1. All properties and OPS components are within expected ranges.

Feature Contribution

Top features for positive contribution

Fingerprint	Bit/Smiles	Feature Structure	Score	Carcinogen in training set
SCFP_6	-347048986	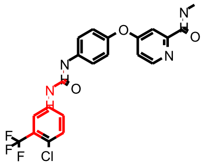 [*]C(=[*])N[c]1:[cH]:[cH]:[*]:[cH]:[cH]:1	0.615	5 out of 7

SCFP_6	-754059116	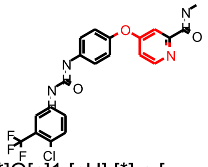<br>[*]O[c]1:[cH]:[*]:n:[cH]:[cH]:1	0.415	1 out of 1
SCFP_6	-531283893	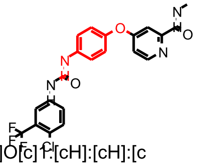<br>[*]O[c]1:[cH]:[cH]:[c](NC(=[*])[*]):[cH]:[cH]:1	0.273	2 out of 4
**Top Features for negative contribution**				
**Fingerprint**	**Bit/Smiles**	**Feature Structure**	**Score**	**Carcinogen in training set**
SCFP_6	-827073191	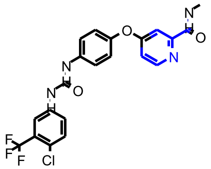<br>[*]C(=[*])[c]1:[cH]:[*]:[cH]:[cH]:n:1	-0.674	0 out of 3
SCFP_6	-975241316	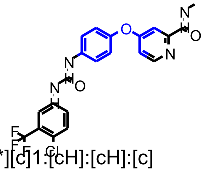<br>[*][c]1:[cH]:[cH]:[c](O[c](:[cH]:[*]):[cH]:[*]):[cH]:[cH]:1	-0.496	0 out of 2
SCFP_6	-488587948	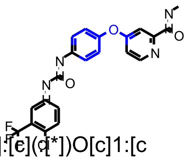<br>[*]:[c](:[*])O[c]1:[cH]:[cH]:[*]:[cH]:[cH]:1	-0.496	0 out of 2

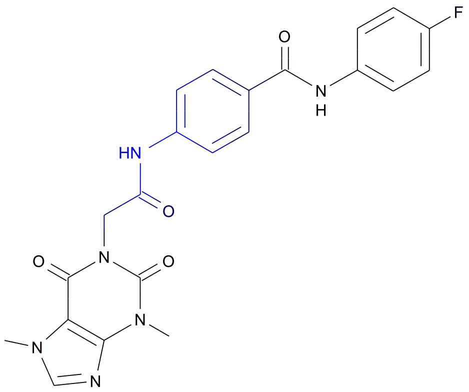

$C_{22}H_{19}FN_6O_4$
Molecular Weight: 450.42246
ALogP: 1.526
Rotatable Bonds: 5
Acceptors: 5
Donors: 2

Model Prediction

Prediction: Non-Irritant
Probability: 0.275
Enrichment: 0.298
Bayesian Score: -5.17
Mahalanobis Distance: 10.4
Mahalanobis Distance p-value: 0.018

Prediction: Positive if the Bayesian score is above the estimated best cutoff value from minimizing the false positive and false negative rate.
Probability: The esimated probability that the sample is in the positive category. This assumes that the Bayesian score follows a normal distribution and is different from the prediction using a cutoff.
Enrichment: An estimate of enrichment, that is, the increased likelihood (versus random) of this sample being in the category.
Bayesian Score: The standard Laplacian-modified Bayesian score.
Mahalanobis Distance: The Mahalanobis distance (MD) is the distance to the center of the training data. The larger the MD, the less trustworthy the prediction.
Mahalanobis Distance p-value: The p-value gives the fraction of training data with an MD greater than or equal to the one for the given sample, assuming normally distributed data. The smaller the p-value, the less trustworthy the prediciton. For highly non-normal X properties (e.g., fingerprints), the MD p-value is wildly inaccurate.

Structural Similar Compounds

Name	2-Anthracenesulfonic acid, 1-amino-9,10-dihydro-9,10-dioxo-4-(2,4,6 -trimethylanilino)-, monosodium salt	Pregna-1,4-diene-3,20-dione, 21-(acetyloxy)-11-hydroxy-6-methyl-17- (1-oxopropoxy)-, (6-alpha,11-beta)-	5-Norbornene-2,3-dicarboxylic acid, 1,4,5,6,7,7-hexachloro-
Structure	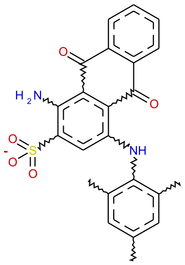	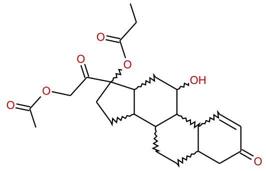	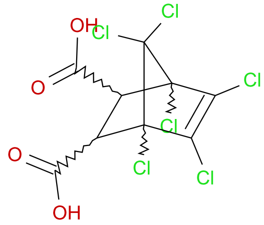
Actual Endpoint	Irritant	Irritant	Irritant
Predicted Endpoint	Non-Irritant	Irritant	Irritant
Distance	0.741	0.793	0.803
Reference	85JCAE "Prehled Prumyslove Toxikologie; Organicke Latky," Marhold, J., Prague , Czechoslovakia, Avicenum, 1986 Volume(issue)/page/year: -,1327,1986	YACHDS Yakuri to Chiryo. Pharmacology and Therapeutics. (Raifu Saiensu Shup pan K.K., 2-5-13, Yaesu, Chuo-ku, Tokyo 104, Japan) V.1-1972- Volume(issue) /page/year: 19,3103,1991	85JCAE "Prehled Prumyslove Toxikologie; Organicke Latky," Marhold, J., Prague , Czechoslovakia, Avicenum, 1986 Volume(issue)/page/year: -,581,1986

Model Applicability

Unknown features are fingerprint features in the query molecule, but not found or appearing too infrequently in the training set.

1. All properties and OPS components are within expected ranges.
2. Unknown FCFP_2 feature: 136150461: [*]:n(:[*])C

Feature Contribution

Top features for positive contribution

Fingerprint	Bit/Smiles	Feature Structure	Score	Irritant in training set

FCFP_12	-1986158408	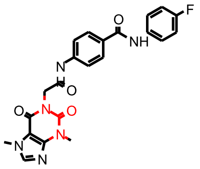 [*]N([*])C(=O)N([*])[*]	0.0821	13 out of 13
FCFP_12	-1539132615	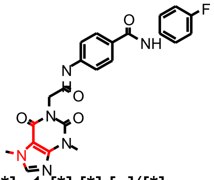 [*]n1:[*]:[*]:[c]([*]):[c]:1C(=[*])[*]	0.0795	9 out of 9
FCFP_12	-1410049896	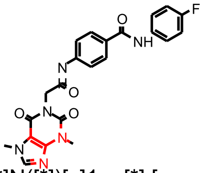 [*]N([*])[c]1:n:[*]:[*]:[c]:1[*]	0.0734	5 out of 5

Top Features for negative contribution

Fingerprint	Bit/Smiles	Feature Structure	Score	Irritant in training set
FCFP_12	1175665944	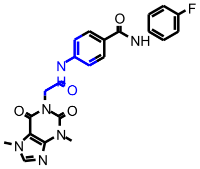 [*]CC(=O)N[c](:[cH]:[*]):[cH]:[*]	-1.02	2 out of 8
FCFP_12	-1838187238	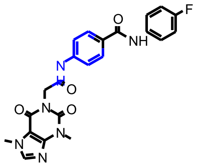 [*]C(=[*])N[c]1:[cH]: [cH]:[*]:[cH]:[cH]:1	-0.692	5 out of 12

FCFP_12	-451043714	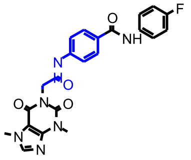 [*]CC(=O)N[c]1:[cH]:[cH]:[c]([*]):[cH]:[cH]:1	-0.65	0 out of 1

Sorafenib

TOPKAT_Skin_Irritancy_None_vs_Irritant

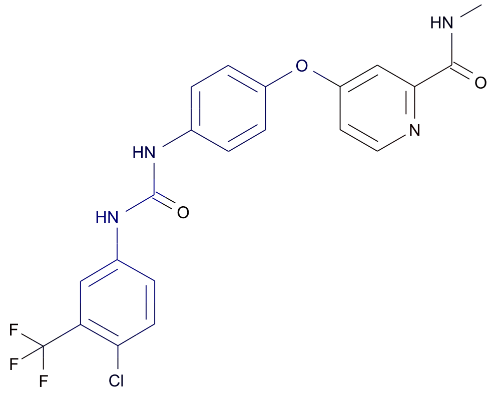

$C_{21}H_{16}ClF_3N_4O_3$

Molecular Weight: 464.82494

ALogP: 4.175

Rotatable Bonds: 6

Acceptors: 4

Donors: 3

Model Prediction

Prediction: Non-Irritant

Probability: 0.264

Enrichment: 0.287

Bayesian Score: -5.23

Mahalanobis Distance: 8.27

Mahalanobis Distance p-value: 0.791

Prediction: Positive if the Bayesian score is above the estimated best cutoff value from minimizing the false positive and false negative rate.
Probability: The esimated probability that the sample is in the positive category. This assumes that the Bayesian score follows a normal distribution and is different from the prediction using a cutoff.
Enrichment: An estimate of enrichment, that is, the increased likelihood (versus random) of this sample being in the category.
Bayesian Score: The standard Laplacian-modified Bayesian score.
Mahalanobis Distance: The Mahalanobis distance (MD) is the distance to the center of the training data. The larger the MD, the less trustworthy the prediction.
Mahalanobis Distance p-value: The p-value gives the fraction of training data with an MD greater than or equal to the one for the given sample, assuming normally distributed data. The smaller the p-value, the less trustworthy the prediciton. For highly non-normal X properties (e.g., fingerprints), the MD p-value is wildly inaccurate.

Structural Similar Compounds

Name	5-Norbornene-2,3-dicarboxylic acid, 1,4,5,6,7,7-hexachloro-	Benzenesulfonic acid, 2,2'-(4,4'-biphenylylenedivinylene)di-, disod ium salt	Sulfide, bis(4-t-butyl-m-cresyl)-
Structure	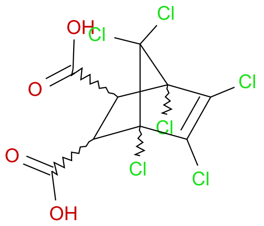	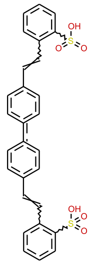	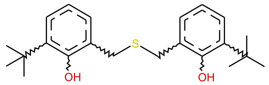
Actual Endpoint	Irritant	Irritant	Irritant
Predicted Endpoint	Irritant	Non-Irritant	Irritant
Distance	0.844	0.871	0.884
Reference	85JCAE "Prehled Prumyslove Toxikologie; Organicke Latky," Marhold, J., Prague , Czechoslovakia, Avicenum, 1986 Volume(issue)/page/year: -,581,1986	MVCRB3 MVC-Report. (Stockholm, Sweden) No.1-2, 1972-73. Discontinued. Volu me(issue)/page/year: 2,193,1973	AMIHBC AMA Archives of Industrial Hygiene and Occupational Medicine. (Chicago , IL) V.2-10, 1950-54. For publisher information, see AEHLAU. Volume(issue)/pag e/year: 5,311,1952

Model Applicability

Unknown features are fingerprint features in the query molecule, but not found or appearing too infrequently in the training set.

1. All properties and OPS components are within expected ranges.

Feature Contribution

Top features for positive contribution				
Fingerprint	Bit/Smiles	Feature Structure	Score	Irritant in training set

FCFP_12	-124655670	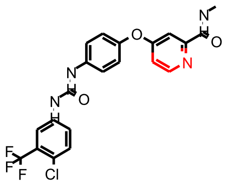<br>[*]:[cH]:[cH]:n:[*]	0.0821	13 out of 13
FCFP_12	-1539132615	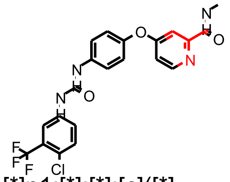<br>[*]n1:[*]:[*]:[c]([*]):[c]:1C(=[*])[*]	0.0795	9 out of 9
FCFP_12	-1695756380	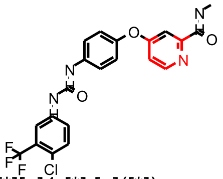<br>[*][c]1:[*]:[c]([*]):n:[cH]:[cH]:1	0.0772	7 out of 7

Top Features for negative contribution

**Fingerprint**	**Bit/Smiles**	**Feature Structure**	**Score**	**Irritant in training set**
FCFP_12	-789307649	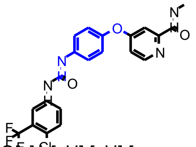<br>[*]O[c]1:[cH]:[cH]:[c](NC(=[*])[*]):[cH]:[cH]:1	-1.54	0 out of 4
FCFP_12	-1838187238	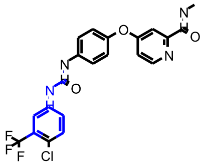<br>[*]C(=[*])N[c]1:[cH]:[cH]:[*]:[cH]:[cH]:1	-0.692	5 out of 12

FCFP_12	1294255210	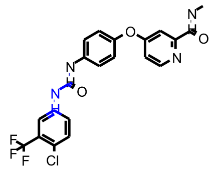 [*]C(=[*])N[c](:[*]):[*]	-0.486	12 out of 22

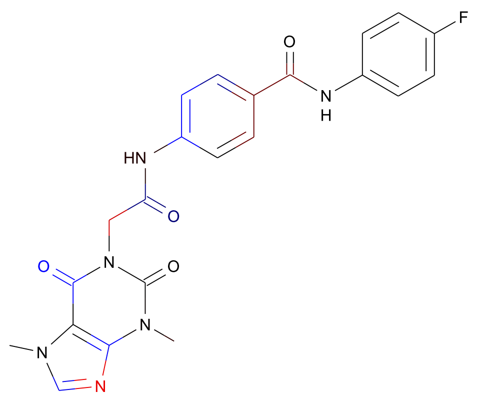

$C_{22}H_{19}FN_6O_4$
Molecular Weight: 450.42246
ALogP: 1.526
Rotatable Bonds: 5
Acceptors: 5
Donors: 2

Model Prediction

Prediction: 9.98
Unit: mg/kg_body_weight/day
Mahalanobis Distance: 11.5
Mahalanobis Distance p-value: 0.0002

Mahalanobis Distance: The Mahalanobis distance (MD) is a generalization of the Euclidean distance that accounts for correlations among the X properties. It is calculated as the distance to the center of the training data. The larger the MD, the less trustworthy the prediction.
Mahalanobis Distance p-value: The p-value gives the fraction of training data with an MD greater than or equal to the one for the given sample, assuming normally distributed data. The smaller the p-value, the less trustworthy the prediciton. For highly non-normal X properties (e.g., fingerprints), the MD p-value is wildly inaccurate.

Structural Similar Compounds

Name	Ochratoxin A	542	470
Structure	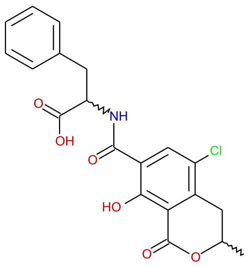	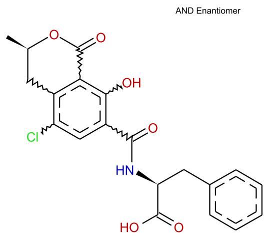	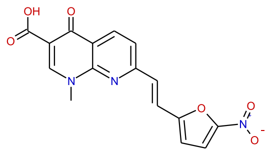
Actual Endpoint (-log C)	4.79932	4.79932	4.62839
Predicted Endpoint (-log C)	3.6353	3.6353	3.93264
Distance	0.736	0.736	0.768
Reference	CPDB	CPDB	CPDB

Model Applicability

Unknown features are fingerprint features in the query molecule, but not found or appearing too infrequently in the training set.

1. OPS PC20 out of range. Value: 4.0965. Training min, max, SD, explained variance: -4.3384, 3.4394, 1.14, 0.0162.
2. Unknown ECFP_2 feature: -960717516: [*]C(=[*])N(C)[c](:[*]):[*]
3. Unknown ECFP_2 feature: -661097313: [*]CN(C(=[*])[*])C(=[*])[*]
4. Unknown ECFP_2 feature: 1135573248: [*]N([*])C(=O)N([*])[*]
5. Unknown ECFP_2 feature: -37698365: [*]N([*])CC(=[*])[*]

Feature Contribution

Top features for positive contribution			
Fingerprint	Bit/Smiles	Feature Structure	Score
ECFP_6	655739385	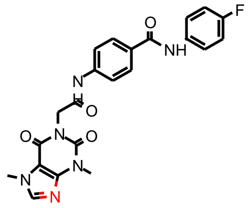 [*]:n:[*]	0.229

ECFP_6	1559650422	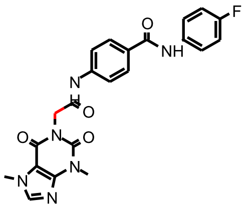 [*]C[*]	0.203
ECFP_6	-175146122	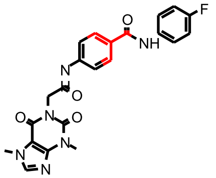 [*]C(=[*])[c](:[cH]:[*]):[cH]:[*]	0.107

Top Features for negative contribution

**Fingerprint**	**Bit/Smiles**	**Feature Structure**	**Score**
ECFP_6	2106656448	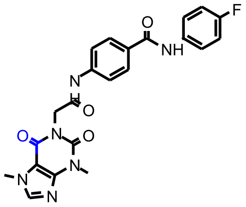 [*]C(=O)[*]	-0.275
ECFP_6	1996767644	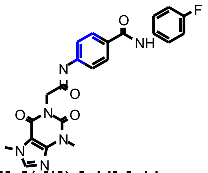 [*][c](:[*]):[cH]:[cH]:[*]	-0.251
ECFP_6	642810091	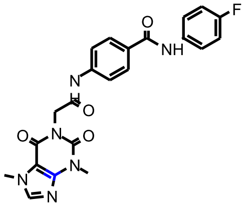 [*][c](:[*]):[*]	-0.247

Sorafenib

TOPKAT_Carcinogenic_Potency_TD50_Mouse

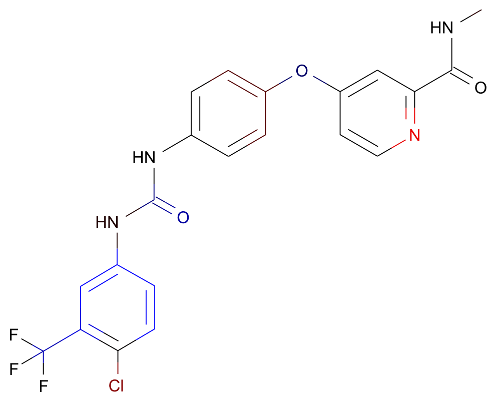

$C_{21}H_{16}ClF_3N_4O_3$

Molecular Weight: 464.82494

ALogP: 4.175

Rotatable Bonds: 6

Acceptors: 4

Donors: 3

Model Prediction

Prediction: 19.2

Unit: mg/kg_body_weight/day

Mahalanobis Distance: 12.4

Mahalanobis Distance p-value: 2.94e-006

Mahalanobis Distance: The Mahalanobis distance (MD) is a generalization of the Euclidean distance that accounts for correlations among the X properties. It is calculated as the distance to the center of the training data. The larger the MD, the less trustworthy the prediction.

Mahalanobis Distance p-value: The p-value gives the fraction of training data with an MD greater than or equal to the one for the given sample, assuming normally distributed data. The smaller the p-value, the less trustworthy the prediciton. For highly non-normal X properties (e.g., fingerprints), the MD p-value is wildly inaccurate.

Structural Similar Compounds

Name	Ochratoxin A	542	4-Chloro-6-(2,3-xylidino)-2-pyri-mi-dinylthio(N-b-hydroxy-ethyl) acetamide
Structure	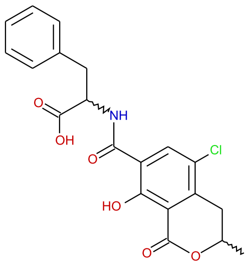	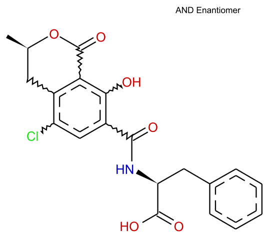	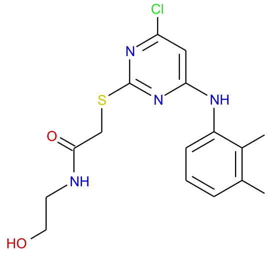
Actual Endpoint (-log C)	4.79932	4.79932	3.91517
Predicted Endpoint (-log C)	3.6353	3.6353	3.92186
Distance	0.718	0.718	0.738
Reference	CPDB	CPDB	CPDB

Model Applicability

Unknown features are fingerprint features in the query molecule, but not found or appearing too infrequently in the training set.

1. All properties and OPS components are within expected ranges.
2. Unknown ECFP_2 feature: 1338334141: [*]C(=[*])NC
3. Unknown ECFP_2 feature: 1413420509: [*]C(=[*])[c](:n:[*]):c:[*]

Feature Contribution

Top features for positive contribution

Fingerprint	Bit/Smiles	Feature Structure	Score
ECFP_6	655739385	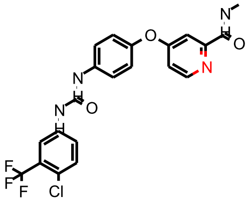 [*]:n:[*]	0.229

ECFP_6	-817402818	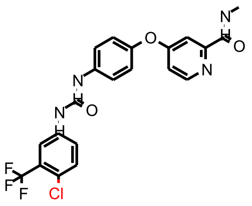 [*]Cl	0.129
ECFP_6	-176455838	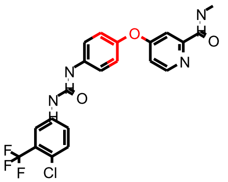 [*]O[c](:[cH]:[*]):[cH]:[*]	0.0818
**Top Features for negative contribution**			
**Fingerprint**	**Bit/Smiles**	**Feature Structure**	**Score**
ECFP_6	1996767644	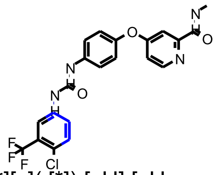 [*][c](:[*]):[cH]:[cH]:[*]	-0.251
ECFP_6	642810091	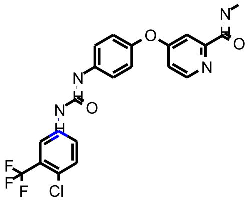 [*][c](:[*]):[*]	-0.247
ECFP_6	-182236392	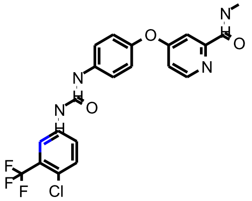 [*]:[cH]:[*]	-0.232

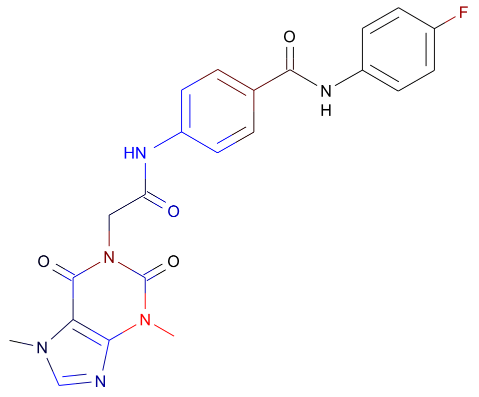

$C_{22}H_{19}FN_6O_4$

Molecular Weight: 450.42246

ALogP: 1.526

Rotatable Bonds: 5

Acceptors: 5

Donors: 2

Model Prediction

Prediction: 5.38

Unit: mg/kg_body_weight/day

Mahalanobis Distance: 16

Mahalanobis Distance p-value: 3.28e-014

Mahalanobis Distance: The Mahalanobis distance (MD) is a generalization of the Euclidean distance that accounts for correlations among the X properties. It is calculated as the distance to the center of the training data. The larger the MD, the less trustworthy the prediction.

Mahalanobis Distance p-value: The p-value gives the fraction of training data with an MD greater than or equal to the one for the given sample, assuming normally distributed data. The smaller the p-value, the less trustworthy the prediciton. For highly non-normal X properties (e.g., fingerprints), the MD p-value is wildly inaccurate.

Structural Similar Compounds

Name	Ochratoxin A	542	4-Bis(2-hydroxyethyl)amino-2-(5-nitro-2-thienyl)quinazoline
Structure	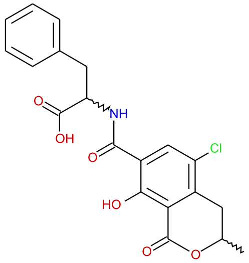	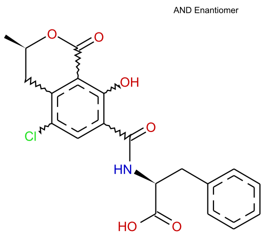	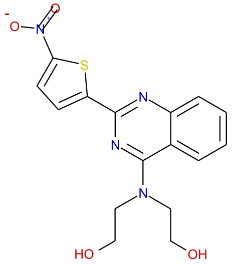
Actual Endpoint (-log C)	6.47264	6.59334	5.05984
Predicted Endpoint (-log C)	5.06501	5.06501	4.23808
Distance	0.732	0.732	0.735
Reference	CPDB	CPDB	CPDB

Model Applicability

Unknown features are fingerprint features in the query molecule, but not found or appearing too infrequently in the training set.

1. All properties and OPS components are within expected ranges.

Feature Contribution

Top features for positive contribution

Fingerprint	Bit/Smiles	Feature Structure	Score
FCFP_6	136627117	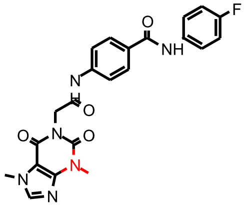 [*]N([*])C	0.69

FCFP_6	1	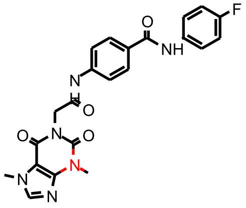 [*]N([*])[*]	0.234
FCFP_6	32	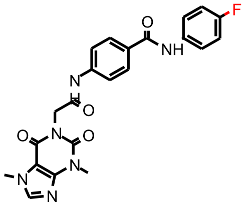 [*]F	0.154
**Top Features for negative contribution**			
**Fingerprint**	**Bit/Smiles**	**Feature Structure**	**Score**
FCFP_6	16	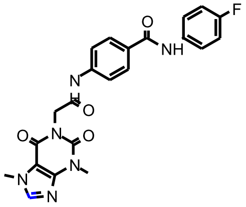 [*]:[cH]:[*]	-0.354
FCFP_6	590925877	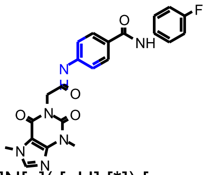 [*]N[c](:[cH]:[*]):[cH]:[*]	-0.323
FCFP_6	566058135	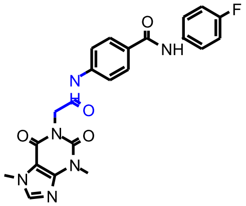 [*]CC(=O)N[*]	-0.182

Sorafenib

TOPKAT_Carcinogenic_Potency_TD50_Rat

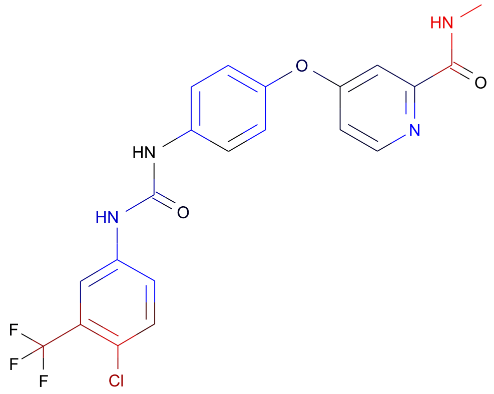

$C_{21}H_{16}ClF_3N_4O_3$
Molecular Weight: 464.82494
ALogP: 4.175
Rotatable Bonds: 6
Acceptors: 4
Donors: 3

Model Prediction

Prediction: 14.2
Unit: mg/kg_body_weight/day
Mahalanobis Distance: 20.4
Mahalanobis Distance p-value: 9.56e-031

Mahalanobis Distance: The Mahalanobis distance (MD) is a generalization of the Euclidean distance that accounts for correlations among the X properties. It is calculated as the distance to the center of the training data. The larger the MD, the less trustworthy the prediction.
Mahalanobis Distance p-value: The p-value gives the fraction of training data with an MD greater than or equal to the one for the given sample, assuming normally distributed data. The smaller the p-value, the less trustworthy the prediciton. For highly non-normal X properties (e.g., fingerprints), the MD p-value is wildly inaccurate.

Structural Similar Compounds

Name	Fluvastatin	913	Ochratoxin A
Structure	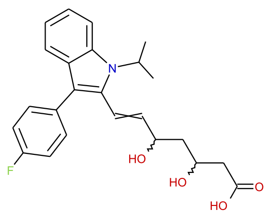	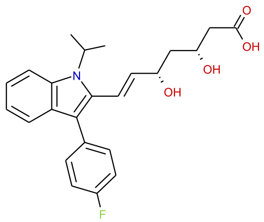	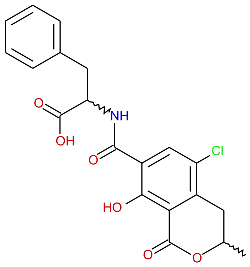
Actual Endpoint (-log C)	3.51742	3.51742	6.47264
Predicted Endpoint (-log C)	5.41573	5.41573	5.06501
Distance	0.597	0.597	0.666
Reference	CPDB	CPDB	CPDB

Model Applicability

Unknown features are fingerprint features in the query molecule, but not found or appearing too infrequently in the training set.

1. All properties and OPS components are within expected ranges.
2. Unknown FCFP_2 feature: -1029533685: [*]:[c](:[*])C(F)(F)F

Feature Contribution

Top features for positive contribution			
**Fingerprint**	**Bit/Smiles**	**Feature Structure**	**Score**
FCFP_6	1	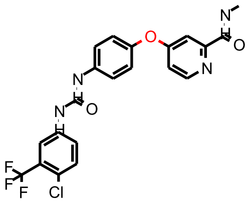 [*]N([*])[*]	0.234

FCFP_6	-885550502	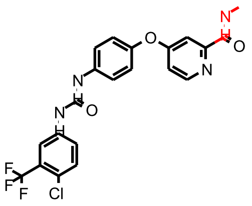 [*]C(=[*])NC	0.229
FCFP_6	32	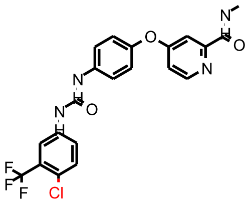 [*]F	0.154

Top Features for negative contribution

Fingerprint	Bit/Smiles	Feature Structure	Score
FCFP_6	16	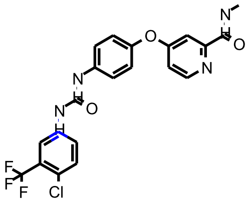 [*]:[cH]:[*]	-0.354
FCFP_6	590925877	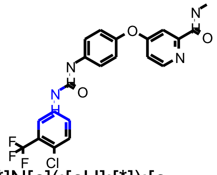 [*]N[c](:[cH]:[*]):[cH]:[*]	-0.323
FCFP_6	1674451008	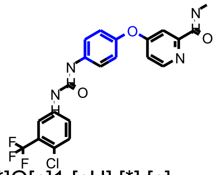 [*]O[c]1:[cH]:[*]:[c]([*]):[cH]:[cH]:1	-0.233

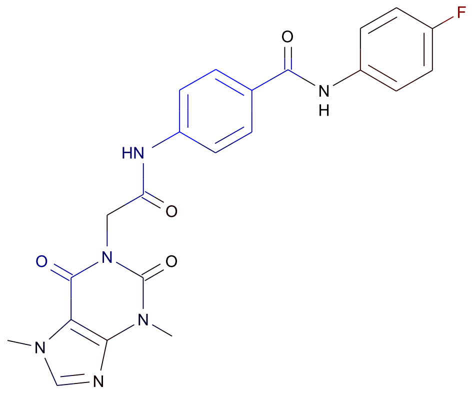

$C_{22}H_{19}FN_6O_4$

Molecular Weight: 450.42246

ALogP: 1.526

Rotatable Bonds: 5

Acceptors: 5

Donors: 2

Model Prediction

Prediction: 0.0141

Unit: g/kg_body_weight

Mahalanobis Distance: 29.3

Mahalanobis Distance p-value: 2.26e-023

Mahalanobis Distance: The Mahalanobis distance (MD) is a generalization of the Euclidean distance that accounts for correlations among the X properties. It is calculated as the distance to the center of the training data. The larger the MD, the less trustworthy the prediction.
Mahalanobis Distance p-value: The p-value gives the fraction of training data with an MD greater than or equal to the one for the given sample, assuming normally distributed data. The smaller the p-value, the less trustworthy the prediciton. For highly non-normal X properties (e.g., fingerprints), the MD p-value is wildly inaccurate.

Structural Similar Compounds

Name	GLIPIZIDE	CHLORSULFURON	DANTROLENE.NA
Structure	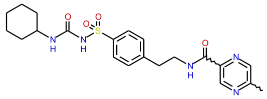	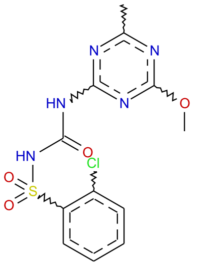	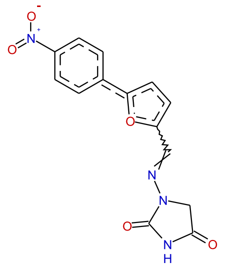
Actual Endpoint (-log C)	3.94991	4.15566	4.19625
Predicted Endpoint (-log C)	3.95594	3.79771	4.62637
Distance	0.660	0.707	0.723
Reference	NDA-17583	EPA COVER SHEET 0027;880301;(1)	NDA-17443

Model Applicability

Unknown features are fingerprint features in the query molecule, but not found or appearing too infrequently in the training set.

1. All properties and OPS components are within expected ranges.
2. Unknown ECFP_6 feature: 672362763: [*]n(:[*]):[*]
3. Unknown ECFP_6 feature: -1046436026: [*]F
4. Unknown ECFP_6 feature: -677309799: [*][c]1:[*]:[*]:[cH]:n:1
5. Unknown ECFP_6 feature: -708878603: [*]n1:[*]:[*]:n:[cH]:1
6. Unknown ECFP_6 feature: -407983022: [*][c]1:[*]:[*]:[cH]:n:1C
7. Unknown ECFP_6 feature: -960717516: [*]C(=[*])N(C)[c](:[*]):[*]
8. Unknown ECFP_6 feature: -509950643: [*]N([*])[c]1:n:[*]:[*]:[c]:1[*]
9. Unknown ECFP_6 feature: -813242890: [*]n1:[*]:[*]:[c]([*]):[c]:1C(=[*])[*]
10. Unknown ECFP_6 feature: 1945129186: [*]N([*])C(=O)[c](:[*]):[*]
11. Unknown ECFP_6 feature: -661097313: [*]CN(C(=[*])[*])C(=[*])[*]
12. Unknown ECFP_6 feature: 1135573248: [*]N([*])C(=O)N([*])[*]
13. Unknown ECFP_6 feature: -37698365: [*]N([*])CC(=[*])[*]
14. Unknown ECFP_6 feature: 1731843802: [*]CC(=O)N[*]
15. Unknown ECFP_6 feature: -177077903: [*]N[c](:[cH]:[*]):[cH]:[*]
16. Unknown ECFP_6 feature: 866343404: [*]N([*])C
17. Unknown ECFP_6 feature: 866450950: [*]:n(:[*])C
18. Unknown ECFP_6 feature: -175146122: [*]C(=[*])[c](:[cH]:[*]):[cH]:[*]
19. Unknown ECFP_6 feature: 1430169877: [*]NC(=O)[c](:[*]):[*]

20. Unknown ECFP_6 feature: -176686665: [*]:[cH]:[c](F):[cH]:[*]
21. Unknown ECFP_6 feature: 220735655: [*]:[c](:[*])F

Feature Contribution

Top features for positive contribution

Fingerprint	Bit/Smiles	Feature Structure	Score
ECFP_6	1559650422	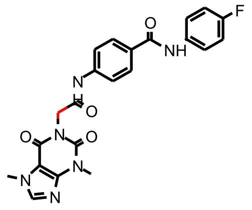 [*]C[*]	0.129
FCFP_6	32	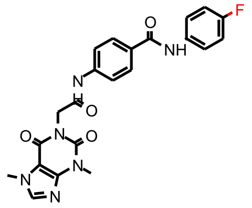 [*]F	0.101
FCFP_6	3	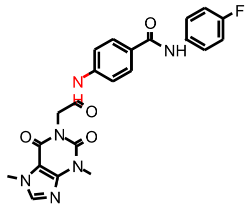 [*]N[*]	0.0924

Top Features for negative contribution

Fingerprint	Bit/Smiles	Feature Structure	Score
ECFP_6	2106656448	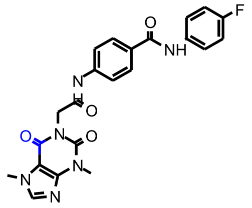 [*]C(=O)[*]	-0.11

FCFP_6	1	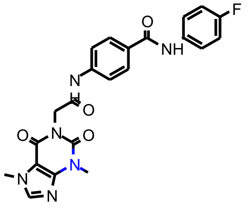 [*]N([*])[*]	-0.102
FCFP_6	-453677277	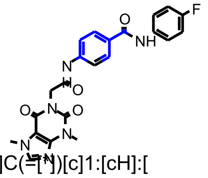 [*]C(=[*])[c]1:[cH]:[*]:[c]([*]):[cH]:[cH]:1	-0.0906

Sorafenib

TOPKAT_Chronic_LOAEL

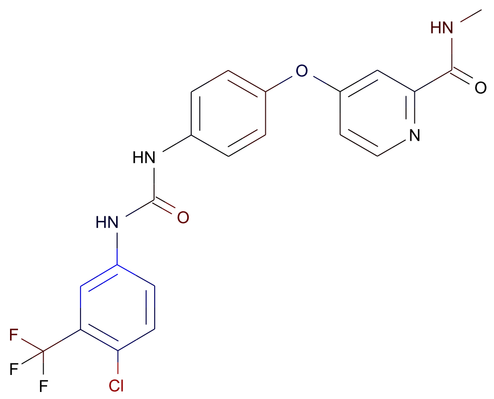

$C_{21}H_{16}ClF_3N_4O_3$
Molecular Weight: 464.82494
ALogP: 4.175
Rotatable Bonds: 6
Acceptors: 4
Donors: 3

Model Prediction

Prediction: 0.00483
Unit: g/kg_body_weight
Mahalanobis Distance: 30
Mahalanobis Distance p-value: 1.21e-024

Mahalanobis Distance: The Mahalanobis distance (MD) is a generalization of the Euclidean distance that accounts for correlations among the X properties. It is calculated as the distance to the center of the training data. The larger the MD, the less trustworthy the prediction.
Mahalanobis Distance p-value: The p-value gives the fraction of training data with an MD greater than or equal to the one for the given sample, assuming normally distributed data. The smaller the p-value, the less trustworthy the prediciton. For highly non-normal X properties (e.g., fingerprints), the MD p-value is wildly inaccurate.

Structural Similar Compounds

Name	GLYBURIDE	D & C RED 9	SODIUM ACIFLUORFEN
Structure	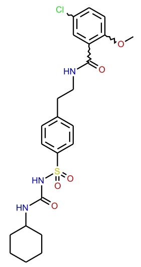	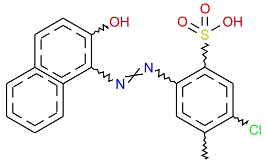	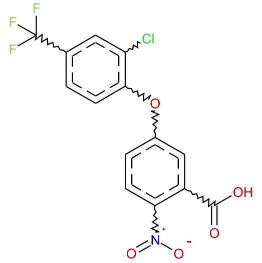
Actual Endpoint (-log C)	4.21661	3.87715	4.16036
Predicted Endpoint (-log C)	4.21035	3.6546	4.65915
Distance	0.636	0.722	0.736
Reference	UPJ-26452	NTP REPORT # 225	EPA COVER SHEET 0192;891101;(1)

Model Applicability

Unknown features are fingerprint features in the query molecule, but not found or appearing too infrequently in the training set.

1. All properties and OPS components are within expected ranges.
2. Unknown ECFP_6 feature: -1046436026: [*]F
3. Unknown ECFP_6 feature: 99947387: [*]:[c](:[*])Cl
4. Unknown ECFP_6 feature: 226796801: [*]C([*])([*])F
5. Unknown ECFP_6 feature: 1305253718: [*]:[c](:[*])O[c](:[*]):[*]
6. Unknown ECFP_6 feature: -677309799: [*][c]1:[*]:[*]:[cH]:n:1
7. Unknown ECFP_6 feature: 1338334141: [*]C(=[*])NC
8. Unknown ECFP_6 feature: -177077903: [*]N[c](:[cH]:[*]):[cH]:[*]
9. Unknown ECFP_6 feature: 1336678434: [*][c](:[*]):[c](:[cH]:[*])C([*])([*])[*]
10. Unknown ECFP_6 feature: -649580166: [*]NC(=O)N[*]
11. Unknown ECFP_6 feature: -1952889961: [*]:[c](:[*])C(F)(F)F
12. Unknown ECFP_6 feature: 1413420509: [*]C(=[*])[c](:[cH]:[*]):n:[*]
13. Unknown ECFP_6 feature: 1996163143: [*]:[cH]:[cH]:n:[*]
14. Unknown ECFP_6 feature: 1430169877: [*]NC(=O)[c](:[*]):[*]
15. Unknown ECFP_6 feature: 864287155: [*]NC

Feature Contribution

Top features for positive contribution

Fingerprint	Bit/Smiles	Feature Structure	Score
ECFP_6	-176455838	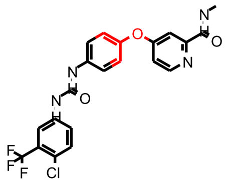 [*]O[c](:[cH]:[*]):[cH]:[*]	0.106
FCFP_6	32	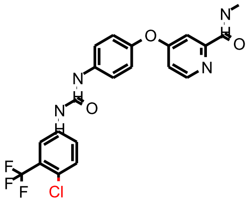 [*]F	0.101
FCFP_6	3	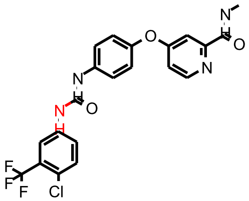 [*]N[*]	0.0924
**Top Features for negative contribution**			
**Fingerprint**	**Bit/Smiles**	**Feature Structure**	**Score**
FCFP_6	1	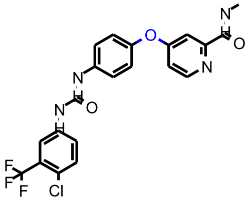 [*]N([*])[*]	-0.102

ECFP_6	-1236483485	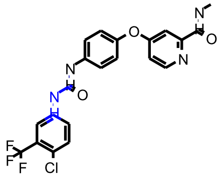<br>[*]C(=[*])N[c](:[*]):[*]	-0.0747
FCFP_6	203677720	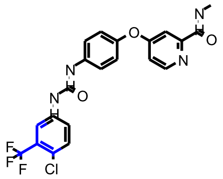<br>[*]C(=[*])[c](:[cH]:[*]):[cH]:[*]	-0.0713

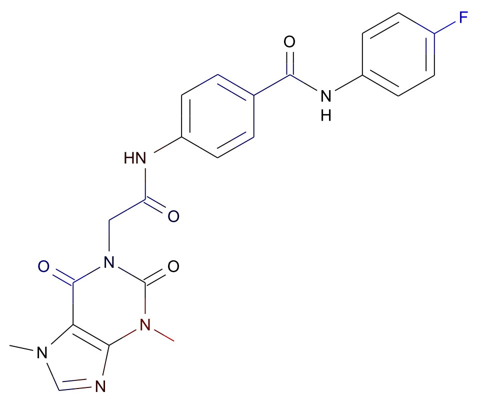

$C_{22}H_{19}FN_6O_4$
Molecular Weight: 450.42246
ALogP: 1.526
Rotatable Bonds: 5
Acceptors: 5
Donors: 2

Model Prediction

Prediction: 0.0467
Unit: g/kg_body_weight
Mahalanobis Distance: 10.4
Mahalanobis Distance p-value: 8.69e-006

Mahalanobis Distance: The Mahalanobis distance (MD) is a generalization of the Euclidean distance that accounts for correlations among the X properties. It is calculated as the distance to the center of the training data. The larger the MD, the less trustworthy the prediction.
Mahalanobis Distance p-value: The p-value gives the fraction of training data with an MD greater than or equal to the one for the given sample, assuming normally distributed data. The smaller the p-value, the less trustworthy the prediciton. For highly non-normal X properties (e.g., fingerprints), the MD p-value is wildly inaccurate.

Structural Similar Compounds

Name	FUROSEMIDE	SALICYLAZOSULFAPYRIDINE	DAPSONE
Structure	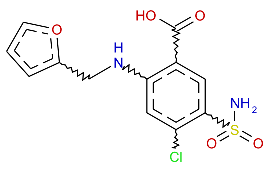	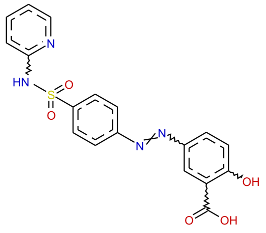	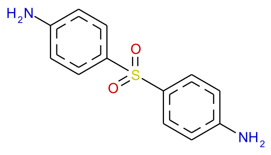
Actual Endpoint (-log C)	4.04236	3.375	3.66258
Predicted Endpoint (-log C)	2.8614	2.80292	3.26993
Distance	0.643	0.770	0.812
Reference	NCI/NTP TR-356	NCI/NTP TR-457	NCI/NTP TR-20

Model Applicability

Unknown features are fingerprint features in the query molecule, but not found or appearing too infrequently in the training set.

1. All properties and OPS components are within expected ranges.
2. Unknown FCFP_2 feature: -124685461: [*]n1:[*]:[*]:n:[cH]:1
3. Unknown FCFP_2 feature: -306856457: [*][c]1:[*]:[*]:[cH]:n:1C
4. Unknown FCFP_2 feature: 136150461: [*]:n(:[*])C

Feature Contribution

Top features for positive contribution

Fingerprint	Bit/Smiles	Feature Structure	Score
FCFP_2	136627117	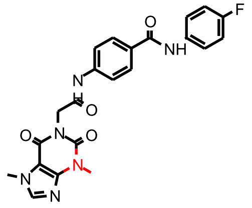 [*]N([*])C	0.173

FCFP_2	3	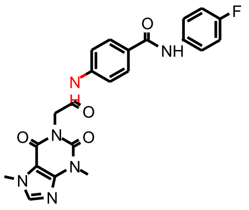 [*]N[*]	0.0737
FCFP_2	17	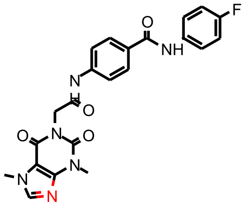 [*]:n:[*]	0.0441

Top Features for negative contribution

Fingerprint	Bit/Smiles	Feature Structure	Score
FCFP_2	71476542	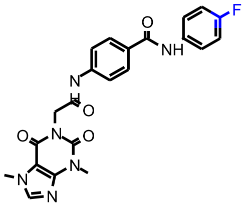 [*]:[c](:[*])F	-0.134
FCFP_2	1872154524	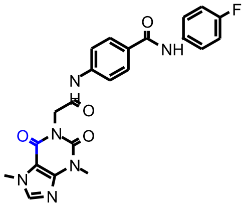 [*]C(=O)[*]	-0.105
FCFP_2	203677720	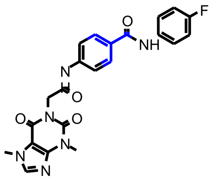 [*]C(=[*])[c](:[cH]:[*]):[cH]:[*]	-0.0829

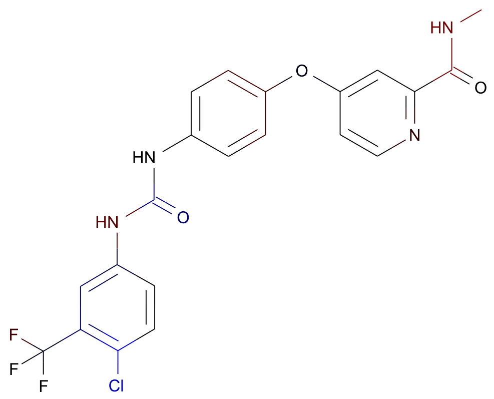

$C_{21}H_{16}ClF_3N_4O_3$
Molecular Weight: 464.82494
ALogP: 4.175
Rotatable Bonds: 6
Acceptors: 4
Donors: 3

Model Prediction

Prediction: 0.0885
Unit: g/kg_body_weight
Mahalanobis Distance: 12.4
Mahalanobis Distance p-value: 1.76e-009

Mahalanobis Distance: The Mahalanobis distance (MD) is a generalization of the Euclidean distance that accounts for correlations among the X properties. It is calculated as the distance to the center of the training data. The larger the MD, the less trustworthy the prediction.
Mahalanobis Distance p-value: The p-value gives the fraction of training data with an MD greater than or equal to the one for the given sample, assuming normally distributed data. The smaller the p-value, the less trustworthy the prediciton. For highly non-normal X properties (e.g., fingerprints), the MD p-value is wildly inaccurate.

Structural Similar Compounds

Name	FUROSEMIDE	PHENOLPHTHALEIN	DISPERSE YELLOW 3
Structure	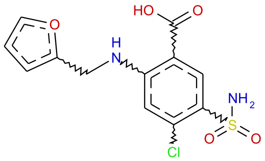	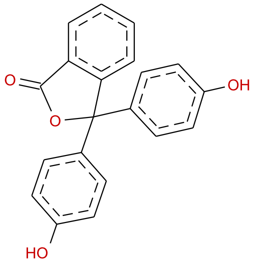	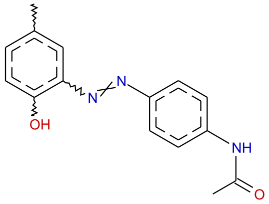
Actual Endpoint (-log C)	4.04236	2.20184	2.77703
Predicted Endpoint (-log C)	2.8614	2.8857	2.80195
Distance	0.741	0.780	0.799
Reference	NCI/NTP TR-356	NCI/NTP TR-465	NCI/NTP TR-222

Model Applicability

Unknown features are fingerprint features in the query molecule, but not found or appearing too infrequently in the training set.

1. All properties and OPS components are within expected ranges.

Feature Contribution

Top features for positive contribution

Fingerprint	Bit/Smiles	Feature Structure	Score
FCFP_2	-885550502	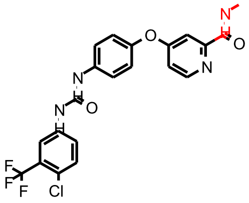 [*]C(=[*])NC	0.115

FCFP_2	3	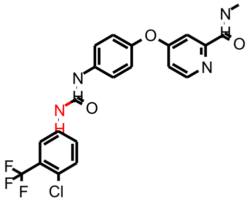 [*]N[*]	0.0737
FCFP_2	332760439	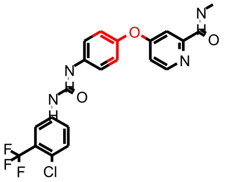 [*]O[c](:[cH]:[*]):[cH]:[*]	0.0611

Top Features for negative contribution

Fingerprint	Bit/Smiles	Feature Structure	Score
FCFP_2	71476542	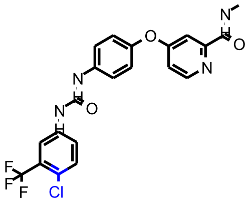 [*]:[c](:[*])F	-0.134
FCFP_2	1872154524	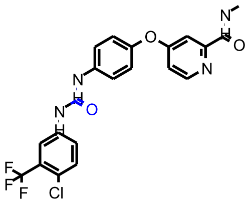 [*]C(=O)[*]	-0.105
FCFP_2	203677720	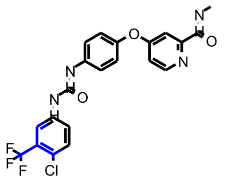 [*]C(=[*])[c](:[cH]:[*]):[cH]:[*]	-0.0829

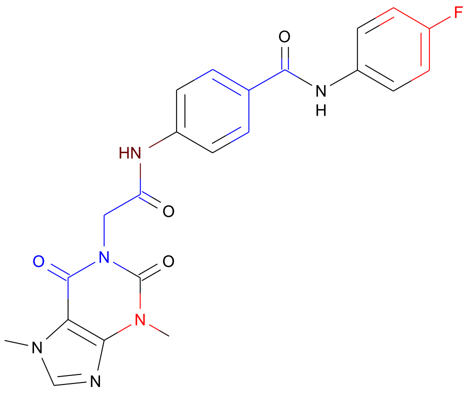

$C_{22}H_{19}FN_6O_4$
Molecular Weight: 450.42246
ALogP: 1.526
Rotatable Bonds: 5
Acceptors: 5
Donors: 2

Structural Similar Compounds

Name	OCHRATOXIN	SULFISOOXAZOLE	PENICILLIN VK
Structure	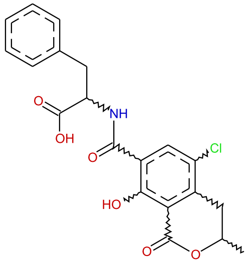	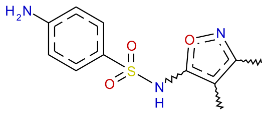	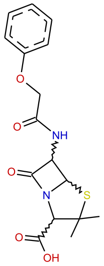
Actual Endpoint (-log C)	6.28396	2.82494	2.54455
Predicted Endpoint (-log C)	5.12358	3.0705	3.9702
Distance	0.757	0.826	0.956
Reference	NCI/NTP TR-358	NCI/NTP TR-138	NCI/NTP TR-336

Model Prediction

Prediction: 0.0163
Unit: g/kg_body_weight
Mahalanobis Distance: 11.5
Mahalanobis Distance p-value: 4.88e-008

Mahalanobis Distance: The Mahalanobis distance (MD) is a generalization of the Euclidean distance that accounts for correlations among the X properties. It is calculated as the distance to the center of the training data. The larger the MD, the less trustworthy the prediction.
Mahalanobis Distance p-value: The p-value gives the fraction of training data with an MD greater than or equal to the one for the given sample, assuming normally distributed data. The smaller the p-value, the less trustworthy the prediciton. For highly non-normal X properties (e.g., fingerprints), the MD p-value is wildly inaccurate.

Model Applicability

Unknown features are fingerprint features in the query molecule, but not found or appearing too infrequently in the training set.

1. Molecular_Weight out of range. Value: 450.42. Training min, max, mean, SD: 68.074, 434.63, 171.13, 85.06.
2. Num_AromaticRings out of range. Value: 3. Training min, max, mean, SD: 0, 2, 0.5625, 0.693.
3. OPS PC5 out of range. Value: -4.7949. Training min, max, SD, explained variance: -3.4, 4.1587, 1.489, 0.0686.
4. OPS PC7 out of range. Value: -3.1412. Training min, max, SD, explained variance: -2.8003, 2.9332, 1.16, 0.0416.
5. Unknown FCFP_2 feature: -124685461: [*]n1:[*]:[*]:n:[cH]:1
6. Unknown FCFP_2 feature: -306856457: [*][c]1:[*]:[*]:[cH]:n:1C
7. Unknown FCFP_2 feature: -1410049896: [*]N([*])[c]1:n:[*]:[*]:[c]:1[*]
8. Unknown FCFP_2 feature: -1986158408: [*]N([*])C(=O)N([*])[*]
9. Unknown FCFP_2 feature: 136150461: [*]:n(:[*])C

Feature Contribution

Top features for positive contribution

Fingerprint	Bit/Smiles	Feature Structure	Score

FCFP_2	32	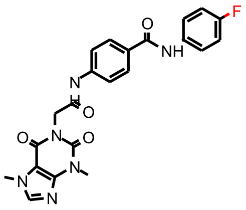 [*]F	0.526
FCFP_2	1	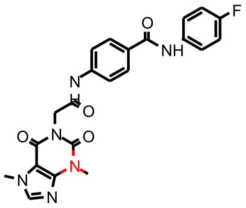 [*]N([*])[*]	0.511
FCFP_2	367998008	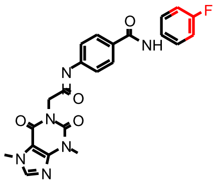 [*]:[cH]:[c](F):[cH]: [*]	0.413

Top Features for negative contribution

Fingerprint	Bit/Smiles	Feature Structure	Score
FCFP_2	203677720	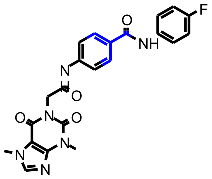 [*]C(=[*])[c](:[cH]:[*]):[cH]:[*]	-0.406
FCFP_2	1872154524	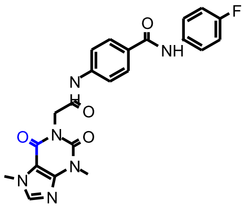 [*]C(=O)[*]	-0.307

FCFP_2	0	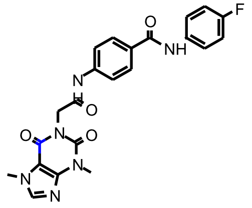 [*]C(=[*])[*]	-0.29

Sorafenib

TOPKAT_Rat_Maximum_Tolerated_Dose_Gavage

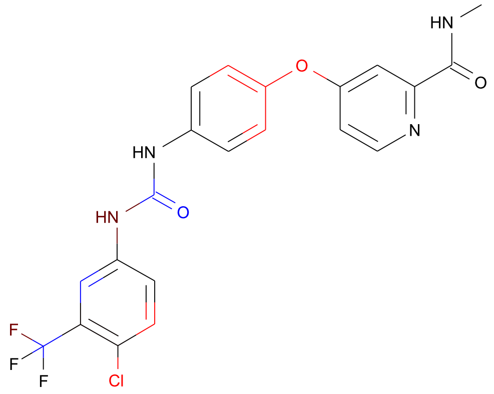

$C_{21}H_{16}ClF_3N_4O_3$

Molecular Weight: 464.82494

ALogP: 4.175

Rotatable Bonds: 6

Acceptors: 4

Donors: 3

Model Prediction

Prediction: 0.000918

Unit: g/kg_body_weight

Mahalanobis Distance: 12.2

Mahalanobis Distance p-value: 4.69e-009

Mahalanobis Distance: The Mahalanobis distance (MD) is a generalization of the Euclidean distance that accounts for correlations among the X properties. It is calculated as the distance to the center of the training data. The larger the MD, the less trustworthy the prediction.
Mahalanobis Distance p-value: The p-value gives the fraction of training data with an MD greater than or equal to the one for the given sample, assuming normally distributed data. The smaller the p-value, the less trustworthy the prediciton. For highly non-normal X properties (e.g., fingerprints), the MD p-value is wildly inaccurate.

Structural Similar Compounds

Name	OCHRATOXIN	SULFISOOXAZOLE	PENICILLIN VK
Structure	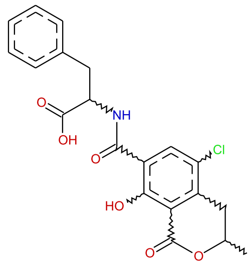	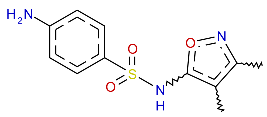	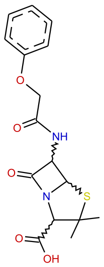
Actual Endpoint (-log C)	6.28396	2.82494	2.54455
Predicted Endpoint (-log C)	5.12358	3.0705	3.9702
Distance	0.758	0.997	1.159
Reference	NCI/NTP TR-358	NCI/NTP TR-138	NCI/NTP TR-336

Model Applicability

Unknown features are fingerprint features in the query molecule, but not found or appearing too infrequently in the training set.

1. Molecular_Weight out of range. Value: 464.82. Training min, max, mean, SD: 68.074, 434.63, 171.13, 85.06.
2. Num_AromaticRings out of range. Value: 3. Training min, max, mean, SD: 0, 2, 0.5625, 0.693.
3. OPS PC5 out of range. Value: -3.5737. Training min, max, SD, explained variance: -3.4, 4.1587, 1.489, 0.0686.
4. OPS PC7 out of range. Value: -3.8342. Training min, max, SD, explained variance: -2.8003, 2.9332, 1.16, 0.0416.
5. Unknown FCFP_2 feature: 1499521844: [*]NC(=O)N[*]
6. Unknown FCFP_2 feature: -1029533685: [*]:[c](:[*])C(F)(F)F
7. Unknown FCFP_2 feature: 136686699: [*]NC

Feature Contribution

Top features for positive contribution			
Fingerprint	Bit/Smiles	Feature Structure	Score

FCFP_2	332760439	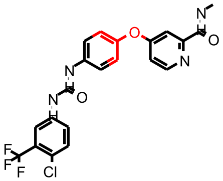 [*]O[c](:[cH]:[*]):[cH]:[*]	0.672
FCFP_2	32	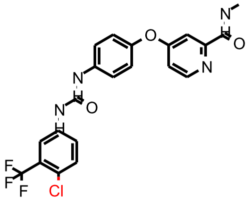 [*]F	0.526
FCFP_2	1	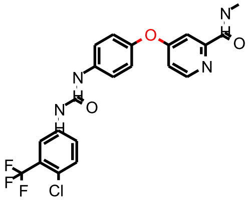 [*]N([*])[*]	0.511

Top Features for negative contribution

Fingerprint	Bit/Smiles	Feature Structure	Score
FCFP_2	203677720	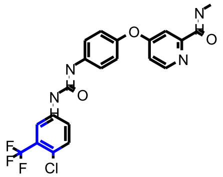 [*]C(=[*])[c](:[cH]:[*]):[cH]:[*]	-0.406
FCFP_2	1872154524	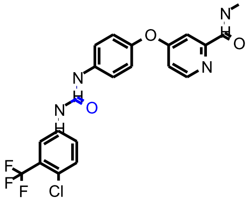 [*]C(=O)[*]	-0.307

FCFP_2	0	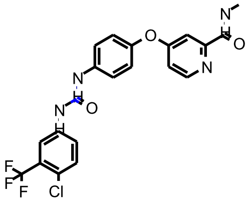<br>[*]C(=[*])[*]	-0.29

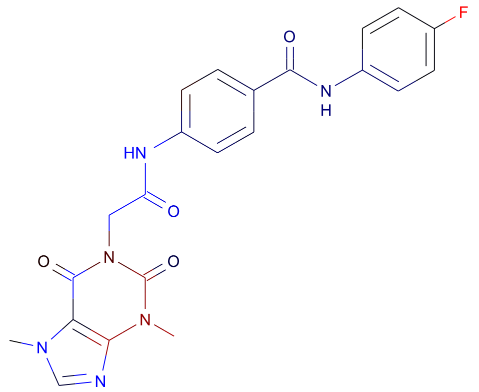

$C_{22}H_{19}FN_6O_4$
Molecular Weight: 450.42246
ALogP: 1.526
Rotatable Bonds: 5
Acceptors: 5
Donors: 2

Structural Similar Compounds

Name	PRASOZIN .HCl (HCl STRIPPED)	SULFAQUINOXALINE	PIRETANIDE
Structure	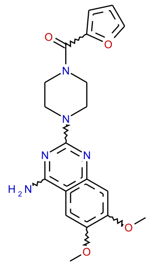	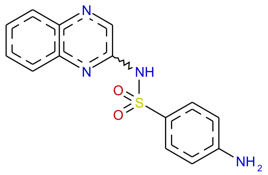	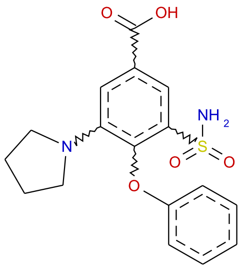
Actual Endpoint (-log C)	2.294	2.341	1.811
Predicted Endpoint (-log C)	3.00765	2.42674	1.83976
Distance	0.665	0.696	0.708
Reference	NIIRDN 6;688;82	MahWM# 16NOV82	DRFUD4 2;393;77

Model Prediction

Prediction: 2.05
Unit: g/kg_body_weight
Mahalanobis Distance: 21.2
Mahalanobis Distance p-value: 3.34e-013

Mahalanobis Distance: The Mahalanobis distance (MD) is a generalization of the Euclidean distance that accounts for correlations among the X properties. It is calculated as the distance to the center of the training data. The larger the MD, the less trustworthy the prediction.
Mahalanobis Distance p-value: The p-value gives the fraction of training data with an MD greater than or equal to the one for the given sample, assuming normally distributed data. The smaller the p-value, the less trustworthy the prediciton. For highly non-normal X properties (e.g., fingerprints), the MD p-value is wildly inaccurate.

Model Applicability

Unknown features are fingerprint features in the query molecule, but not found or appearing too infrequently in the training set.

1. All properties and OPS components are within expected ranges.
2. Unknown FCFP_6 feature: 16: [*]:[cH]:[*]
3. Unknown FCFP_6 feature: 1747237384: [*][c]1:[*]:[*]:[cH]:n:1
4. Unknown FCFP_6 feature: -124685461: [*]n1:[*]:[*]:n:[cH]:1
5. Unknown FCFP_6 feature: -306856457: [*][c]1:[*]:[*]:[cH]:n:1C
6. Unknown FCFP_6 feature: -1410049896: [*]N([*])[c]1:n:[*]:[*]:[c]:1[*]
7. Unknown FCFP_6 feature: 136150461: [*]:n(:[*])C
8. Unknown FCFP_6 feature: 1618154665: [*][c](:[*]):[cH]:[cH]:[*]
9. Unknown FCFP_6 feature: 71476542: [*]:[c](:[*])F

Feature Contribution

Top features for positive contribution			
Fingerprint	Bit/Smiles	Feature Structure	Score

ECFP_6	-1046436026	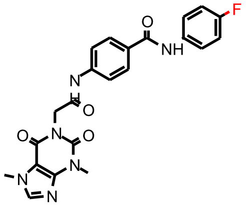 [*]F	0.349
ECFP_6	642810091	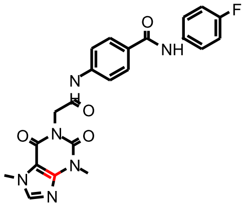 [*][c](:[*]):[*]	0.281
ECFP_6	-1897341097	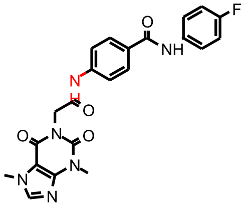 [*]N[*]	0.216
**Top Features for negative contribution**			
**Fingerprint**	**Bit/Smiles**	**Feature Structure**	**Score**
ECFP_6	2106656448	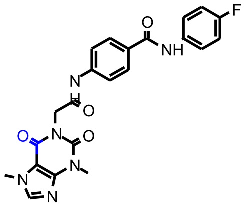 [*]C(=O)[*]	-0.352
ECFP_6	655739385	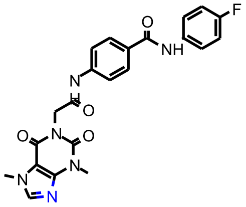 [*]:n:[*]	-0.239

FCFP_6	566058135	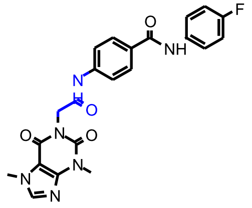 [*]CC(=O)N[*]	-0.216

Sorafenib

TOPKAT_Rat_Oral_LD50

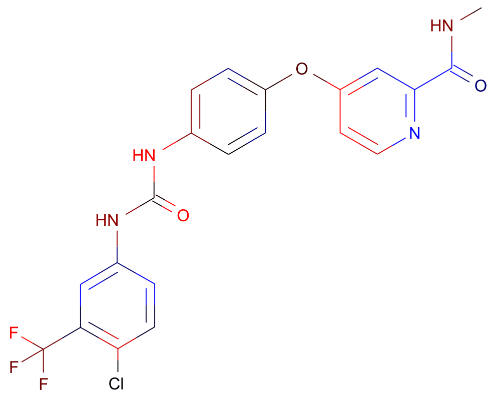

$C_{21}H_{16}ClF_3N_4O_3$

Molecular Weight: 464.82494

ALogP: 4.175

Rotatable Bonds: 6

Acceptors: 4

Donors: 3

Structural Similar Compounds

Name	FLUBENDAZOLE	PHOSPHORAMIDOTHIOIC ACID; ACETIMIDOYL-; O;O-bis-(p-CHLOROPHENYL)ESTER	BEZAFIBRATE
Structure	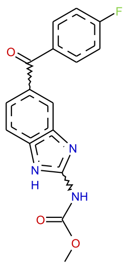	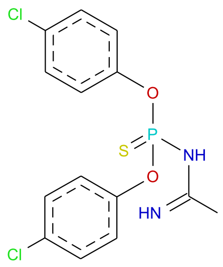	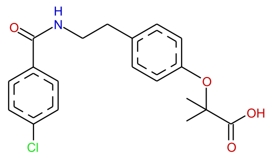
Actual Endpoint (-log C)	2.088	5.006	1.946
Predicted Endpoint (-log C)	2.69288	3.23989	2.54395
Distance	0.697	0.703	0.721
Reference	YRTMA6 9;11;78	FMCHA2 -;C149;89	ARZNAD 30;2023;80

Model Prediction

Prediction: 0.823

Unit: g/kg_body_weight

Mahalanobis Distance: 21

Mahalanobis Distance p-value: 1.93e-012

Mahalanobis Distance: The Mahalanobis distance (MD) is a generalization of the Euclidean distance that accounts for correlations among the X properties. It is calculated as the distance to the center of the training data. The larger the MD, the less trustworthy the prediction.
Mahalanobis Distance p-value: The p-value gives the fraction of training data with an MD greater than or equal to the one for the given sample, assuming normally distributed data. The smaller the p-value, the less trustworthy the prediciton. For highly non-normal X properties (e.g., fingerprints), the MD p-value is wildly inaccurate.

Model Applicability

Unknown features are fingerprint features in the query molecule, but not found or appearing too infrequently in the training set.

1. All properties and OPS components are within expected ranges.
2. Unknown FCFP_6 feature: 16: [*]:[cH]:[*]
3. Unknown FCFP_6 feature: 71476542: [*]:[c](:[*])F
4. Unknown FCFP_6 feature: 1747237384: [*][c]1:[*]:[*]:[cH]:n:1
5. Unknown FCFP_6 feature: 1618154665: [*][c](:[*]):[cH]:[cH]:[*]
6. Unknown FCFP_6 feature: 136686699: [*]NC

Feature Contribution

Top features for positive contribution			
**Fingerprint**	**Bit/Smiles**	**Feature Structure**	**Score**

FCFP_6	71953198	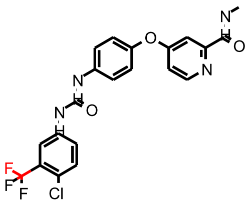 [*]C([*])([*])F	0.392
ECFP_6	-1046436026	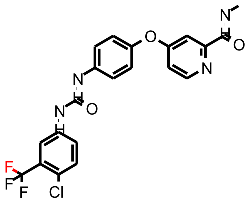 [*]F	0.349
ECFP_6	642810091	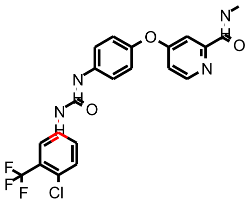 [*][c](:[*]):[*]	0.281

Top Features for negative contribution

Fingerprint	Bit/Smiles	Feature Structure	Score
ECFP_6	226796801	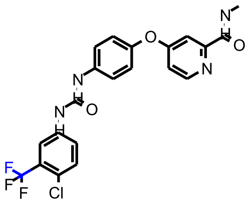 [*]C([*])([*])F	-0.32
ECFP_6	-817402818	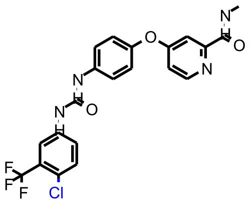 [*]Cl	-0.263

ECFP_6	-176455838	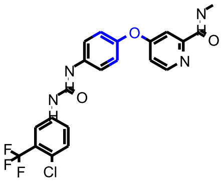<br>[*]O[c](:[cH]:[*]):[cH]:[*]	-0.257
